# Roles of Intragenic and Intergenic L1s in Mouse and Human

**DOI:** 10.1371/journal.pone.0113434

**Published:** 2014-11-19

**Authors:** Chumpol Ngamphiw, Sissades Tongsima, Apiwat Mutirangura

**Affiliations:** 1 Inter-Department Program of Biomedical Sciences, Faculty of Graduate School, Chulalongkorn University, Bangkok, Thailand; 2 Genome Technology Research Unit, National Center for Genetic Engineering and Biotechnology (BIOTEC), National Science and Technology Development Agency (NSTDA), Pathum Thani, Thailand; 3 Center of Excellence in Molecular Genetics of Cancer and Human Diseases, Department of Anatomy, Faculty of Medicine, Chulalongkorn University, Bangkok, Thailand; CNRS, France

## Abstract

Long INterspersed Element-1 (LINE-1 or L1) is a retrotransposable element that has shaped the evolution of mammalian genomes. There is increasing evidence that transcriptionally active L1 could have been co-opted through evolution to play various roles including X-inactivation, homologous recombination and gene regulation. Here, we compare putatively active L1 distributions in the mouse with human. L1 density is higher in the mouse except for the Y-chromosome. L1 density is the highest in X-chromosome, implying an X-inactivation role. L1 is more common outside genes (intergenic) except for the Y-chromosome in both species. The structure of mouse L1 is distinguished from human L1 by the presence of a 200 bp repeat in the 5′ UTR of the former. We found that mouse intragenic L1 has significantly higher repeat copy numbers than intergenic L1, suggesting that this is important for control of L1 expression. Furthermore, a significant association between the presence of intragenic L1s and down-regulated genes in early embryogenesis was found in both species. In conclusion, the distribution of L1 in the mouse genome points to biological roles of L1 in mouse similar to human.

## Introduction

The Long INterspersed Element-1 (LINE-1 or L1) is a retrotransposable element, which constitutes 18–20% of mammalian genomes [1], [2]. The frequencies of L1s differ among closely related mammalian lineages [3], [4] and thus active L1s may still be a major driver of mammalian genome evolution. There are about 500,000 copies of L1 in the human genome, whereas the mouse genome has almost 600,000 copies [1], [2]. A full-length human L1 (∼6,000 nucleotides) is shorter than that of mouse (∼7,000 nucleotides). Full-length L1s contain two open reading frames encoding proteins essential for retrotransposition, a smaller ORF1 and a larger ORF2 separated by ∼60 bp. The RNA transcribed from active full-length L1s comprises both ORFs flanked by 5′ and 3′ UTRs with a poly-A tail [5], [6].

Although L1s are abundant in human and mouse genomes, most of them do not have retrotranposition activity owing to truncations in 5′ regions, rearrangements, or mutations [7], [8]. According to L1base [9], there are almost 12,000 full-length (>4,500 nucleotides) human L1s, but only 145 of these are considered as potentially active. In contrast, full-length (>5,000 nucleotides) L1s are more numerous in mouse, and the fraction of potentially active elements is considerably higher (16,000 and 2,382, respectively). Although most L1s are inactive, active L1 retrotranposition is an important evolutionary driver of mammalian genome complexity, and is responsible for heritable disorders [10].

Until recently L1s were thought to be selfish DNA elements in which their only function was to replicate [10]. However, L1s may acquire other functions through an evolutionary process depending on the genomic context where they have inserted. Examples of such functions include spreading of X-inactivation [11], [12], [13], control of gene expression (acting as a *cis*-regulatory element in embryogenesis), cell differentiation and DNA repair [14], [15]. Furthermore, intragenic L1s are transcriptionally active during embryogenesis [16] and in cancer cells [17] as a result of hypomethylation. The L1-RNAs act as antisense RNAs that can pair with complementary L1 sequences on the corresponding gene pre mRNA and form a complex with AGO2 in the nucleus to repress gene expression [17], [18]. Transcriptionally active L1s located within genes (intragenic) may thus have been evolutionarily co-opted for roles in gene regulation. These new functions provide a basis for purifying selection to maintain L1 integrity and transcriptional activity.

Mouse and human L1s share similar ORFs but differ markedly in 5′ UTR sequence, which may be responsible for differences in transcriptional activities between the two species [8]. In particular, the 5′ UTRs of full-length human L1s house two internal promoters, sense and antisense [7]. In contrast, the 5′ UTRs of full-length mouse L1s contain a ∼200 bp sequence called monomer that can be tandemly repeated [8]. The number of these monomer repeats vary among mouse L1 families, in which copy number is associated with L1 transcriptional activity [8], [19]. Furthermore, the diversity of L1 elements between the two species means that global comparison is difficult such that simple phylogenetic-based analysis is not informative as discussed in [6], [20].

In order to further explore other possible functions of L1 in mammals, this paper presents an in-depth comparative study between human and mouse L1s. First, the distribution of L1s within and outside of gene bodies (intragenic and intergenic, respectively) were mapped between the two species. Intragenic and intergenic L1s were compared in terms of conservation of L1 structural features in mouse instead of performing straightforward L1 sequence comparison. Finally, statistical tests were performed of intragenic L1 association with gene expression profiles during the early stages of human and mouse embryogenesis.

## Material and Methods

Mouse and human L1 information were downloaded from the L1Base, which is a public database containing L1 elements residing in human and mouse reference genomes [9]. These sequences include full-length intact L1s (putatively active with all functional elements necessary for retrotransposition present), full-length non-intact L1s (lacking some or mutated in functional moieties, which reduce likelihood of mobilization), and intact ORF2 L1s (lacking ORF1 but may assist retrotransposition of *Alu*).

### Distribution of LINE-1 sequences

**Figure 1:**
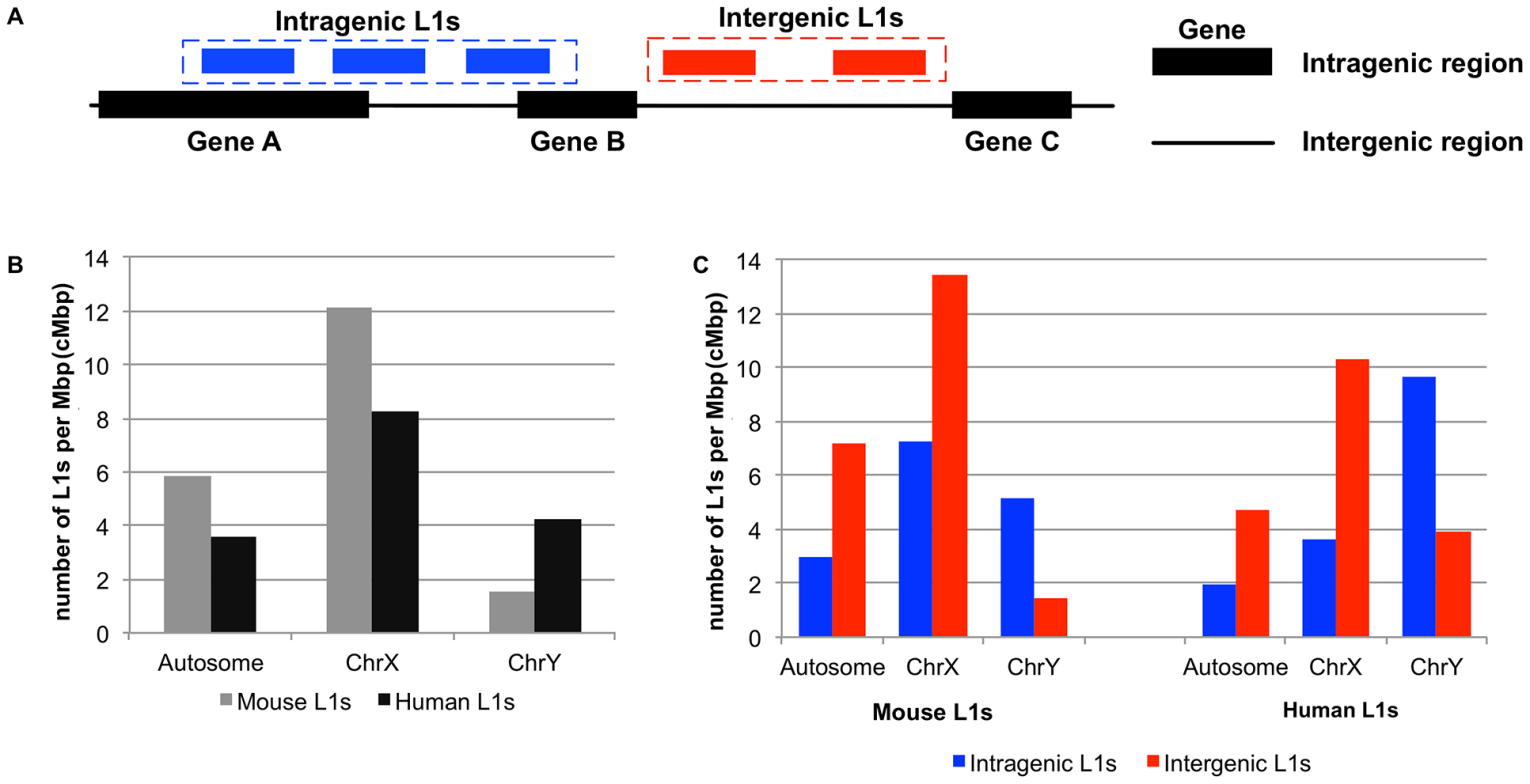
Distribution of mouse and human L1s over their genomes. (A) Graphical definition of intragenic and intergenic L1s. An intragenic L1 is represented by a blue box, while the intergenic one is represented in a red box. The black box represents a gene (intragenic region) and the black line represents an area outside (intergenic region) the gene bodies. (B) A bar graph shows the distribution of mouse (gray columns) and human (black columns) L1s residing on autosome, X, and Y-chromosomes. (C) Two side-by-side bar graphs comparing intragenic (blue columns) vs. intergenic (red columns) L1s on mouse and human genomes.

We categorized L1s into two groups, intragenic and intergenic, based on their genomic locations in NCBI *Homo sapiens* reference sequence (Refseq) build 36.3 and *Mus musculus* mouse Refseq build 35. The intragenic L1 group (Figure 1A) comprises L1s that are *totally* or *partially* located within the gene definition—from the first to the last annotated exon of the largest transcript isoform. All other L1s are defined as intergenic L1 (Figure 1A). There are a total of 11,897 and 16,508 full-length human and mouse L1s, respectively. In human, 2,547 (21.41%) of the total human L1 elements are intragenic, which located in 1,454 human genes. While in mouse, 2,594 elements or 15.71% of the total mouse L1s are intragenic L1s distributed over 1,066 genes. L1s mapped to human and mouse genomes were classified into three classes, namely L1s in autosomes (chromosome 1 to 22 in human and chromosome 1 to 19 in mouse), the X chromosome and the Y chromosome, respectively. L1 density was calculated as L1 counts per million base pairs (cMbp) of the host chromosomal regions. The genome-wide distributions and densities of intragenic and intergenic L1s were calculated separately for the two species.

### Statistical analysis of LINE-1 characteristics and their locations in host genome

We hypothesized that intragenic mouse L1s should more conserved than intergenic, as was found earlier for human [17]. L1 conservation was assessed by analysis of L1 sequence and functional annotations, called feature or characteristic from L1Base. These putative L1 sequences were compared with the full-length L1s, i.e., L1.2 (gi:M80343) for human and L1MdA2 (gi:M13002) for mouse. The intactness (conservation) from each of these characteristics was calculated by comparing it with the corresponding locus on the reference L1s. These characteristics can be used to predict the status of L1 activity [9], [21]. From that definition, conserved means conservation of protein functional motifs and RNA structural elements that altogether are necessary and sufficient for retrotransposition [9]. These functional motifs include ORF boundaries, promoter motifs, poly A terminator, and important amino acid residues [9], [21]. Mouse L1s can be classified into subfamilies according to their monomer signatures located at 5′ UTR of mouse L1s [21]. These subfamilies are F (2,602 elements), A (6,336 elements), T_F_ (4,940 elements), and G_F_ (1,622 elements). Human L1s can be grouped into two major subfamilies according to their sequences in the 3′ end of ORF2 [22], namely L1PA (primate L1s with 10,668 elements) and L1M (mammalian L1s with 969 elements). Such subfamily information are thought to reflect L1 age by using the assumption that sequence divergence increases with age [23], [24].

Two statistical tests, namely chi-square test for categorical characteristics and Student's *t*-test for non-categorical characteristics, were conducted to test the null hypothesis that for a given feature of L1, there should not be much different between intragenic and intergenic L1s. Statistical tests were conducted separately on both mouse and human L1s. In mouse, there are 42 categorical functional characteristics and 11 non-categorical characteristics of L1s (Table S1). In human, there are 33 categorical and 18 non-categorical characteristics of L1s (Table S2). For chi-square tests, 2×2 contingency tables were constructed for every categorical feature, describing relationship between *groups* related to the host genome (intragenic/intergenic) and *condition*, e.g., conserved, CpG islands, and L1 functional feature. Since age of L1s may confound the relative contributions of young and old elements to the intragenic/intergenic regions, we adopted Mantel-Haenszel (MH) chi-square testing model [25] to adjust the confounding effect. MH chi-square operates by combining the chi-square tests performed separately on each L1 stratum (grouped by the aforementioned L1 subfamilies). MH p-values and MH odds ratios (OR) between L1s located in the intragenic and intergenic region were then calculated for each feature. An OR greater than one indicates that the L1 status tested (conserved, etc.) has a higher probability to be intragenic than intergenic.

For non-categorical (quantitative) features, unpaired Student's *t*-tests with unequal variances [26] were performed between intragenic and intergenic L1s. The threshold for significance was p-value  = 1.0E-03. The quantitative features tested for mouse L1s include GC content and intactness score, i.e. global score for the entire L1 sequence, number of monomer and number of monomer splice sites (specific to mouse L1), and numbers of ORF (ORF1 and ORF2) specific features, i.e., number of ORF gaps, ORF stop codons and ORF frameshifts.

### Analysis of intragenic L1s regulating gene expression during embryogenesis

To test the hypothesis that intragenic L1s regulate genes in other physiological cellular processes such as embryogenesis in mammalian species, we analyzed publicly available microarray data from different stages of preimplantation embryonic development, namely one-cell, two-cell, four-cell, eight-cell, sixteen-cell, morula and blastocyst stages (GEO accession number GSE18290 [27]). The gene regulation profiles of one-cell stage were compared with all other stages for human and mouse. Differentially expressed genes between each developmental stage and the one-cell stage were identified using paired Student's *t*-test [28]. Paired *t*-statistics were calculated from the average and standard deviation of differences between paired samples of each developmental stage and the one-cell stage. Genes with p-values less than 0.05 were considered as differentially expressed. Chi-square analysis was then performed to test if genes containing L1 sequences are associated with up regulation with respect to the one-cell stage. The 2×2 contingency tables were constructed with rows of number of genes with L1 present and L1 absent, and columns of number of up-regulated genes and the rest. Similar 2×2 contingency tables were also constructed for testing L1 association with down-regulated genes in which columns were constructed as down-regulated genes and the rest. Chi-square tests were performed for both human and mouse between each pair of time-points using CU-DREAM (http://pioneer.netserv.chula.ac.th/~achatcha/cu-dream/) [29]. Thresholds for significance were p-value <1.0E-03 and OR >1.0.

## Results

### Comparison of L1 chromosomal distributions

First, we determine densities of the intragenic L1 group and intergenic L1 (Figure 1A) on autosome, X and Y chromosomes. Except for the Y chromosome, L1 density is much greater in mouse than that of human (Figure 1B). Intragenic L1 density is lower than intergenic for autosomes and X chromosome of both species, whereas the density of intragenic L1s is greater in the Y chromosome of both species (Figure 1C). The denseness of intragenic L1s in Y-chromosome (Chr. Y) cannot be explained by the compactness of Chr. Y. The percentage of intergenic region is always larger than intragenic region on all chromosomes and is largest for the Y chromosome. In the human genome on average, 58.95% of autosomes are intergenic whereas the intergenic contents of sex chromosomes are higher (68.68% and 94.26% for X and Y respectively). Intergenic contents in mouse are similar (68.30% average of autosomes, 78.54% for X and 96.35% for Y).

### Conservation of intragenic L1s

**Figure 2:**
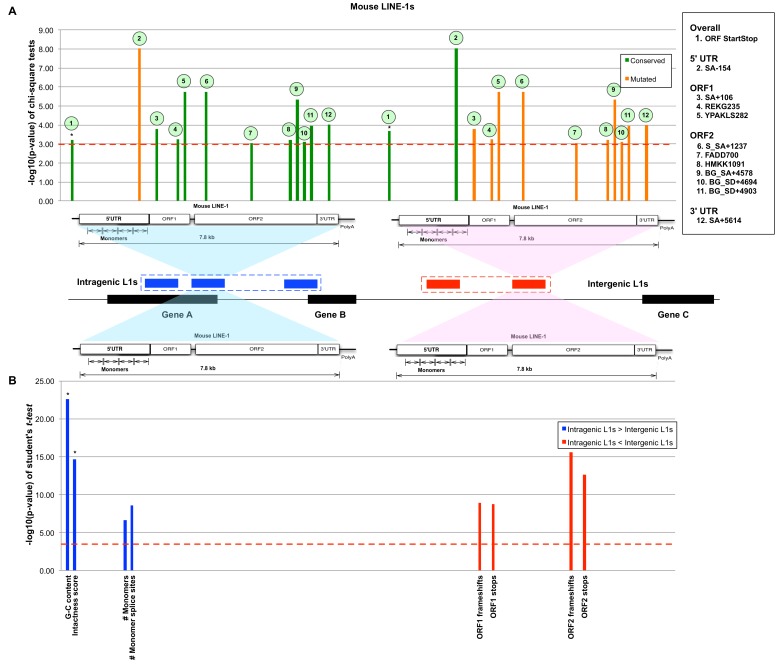
The comparison between intragenic and intergenic mouse L1s. (A) Bar graph of conserved (green columns) and mutated (orange columns) features from Mantel-Haenszel chi-square tests with cutoff of p-value <1.0E-03 (dashed line). The structure of mouse L1 is shown under the bar graph to indicate the relative location of the feature in L1 sequence. The bars marked with an asterisk (*) indicate the features calculated for the entire L1 sequence. (B) A bar graph shows significant non-categorical features with p-value <1.0E-03, using the Student's *t*-test. The blue columns indicate that more of these features appear in the intragenic L1s than that of intergenic ones. The red columns indicate that there are more of such features in the intergenic L1s than that of intragenic ones.

**Figure 3:**
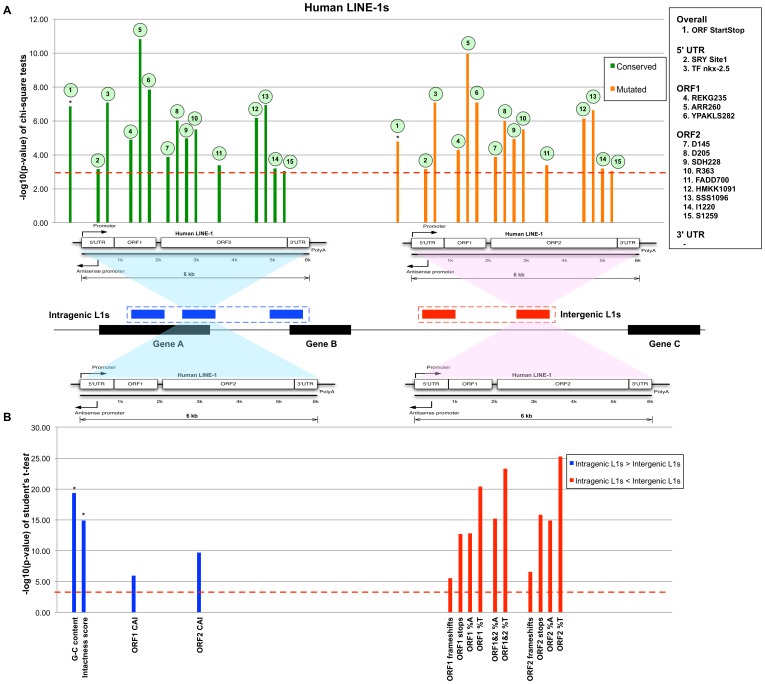
The comparison between intragenic and intergenic human L1s. (A) A bar graph shows 15 significant features passing the significance p-value 1.0E-03 (dashed line) from Mantel-Haenszel chi-square tests. The green and orange bars represent conserved and mutated features, respectively. These colored bars are aligned with L1 structure shown below the graphs. The bars marked with an asterisk (*) indicate the features calculated for the entire L1 sequence. (B) A bar graph shows non-categorical features whose significance p-value pass 1.0E-03 (dashed line). The blue columns indicate that more of these features appear in the intragenic L1s than that of intergenic ones. The red columns indicate that there are more of such features in the intergenic L1s than that of intragenic ones.

Previous study showed that intragenic human L1s are more conserved than intergenic ones [17]. In particular, intragenic L1s have greater GC and CpG island contents. Conversely, sporadic frameshifts, gaps, and stop codons are more common in intergenic L1s. The greater conservation of human intragenic L1 sequences may reflect functions dependent on L1 transcription [17]. The conservation and distinction of intragenic and intergenic L1 sequences in mouse and human were tested by Mantel-Haenszel chi-square and unequal variance Student's *t*-tests (Figure 2 and 3). The Mantel-Haenszel p-value measurement was presented in –log_10_(p-value), where the higher number represents more significant value. From these tests, it was found that intragenic L1s are significantly more conserved in mouse as well as human. In mouse intragenic L1s, conserved features are distributed along the structure of L1 except for the 5′ UTR. Only one functional feature, the SA-154 acceptor splice site on antisense mouse L1 sequences, is poorly conserved among intragenic mouse L1. There are three conserved features in ORF1, six in ORF2 and one in the 3′ UTR. Unlike mouse intragenic L1s, the 5′ UTR of human intragenic L1 contains two conserved features. There are three and nine conserved features in the ORF1 and ORF2 of human intragenic L1, respectively. For both mouse and human, intragenic L1s have significantly higher intactness score and GC contents than that of intergenic L1s. For human L1s, ORF1 and ORF2 codon adaptation indexes (CAI) are significantly higher for intragenic L1s, whereas for mouse intragenic L1s, the monomer features, namely mean number of monomer repeats and monomer splice sites, are significantly greater. The complete listing of functional features and their statistical values are in Table S1 and S2, respectively. Our analyses indicate that many important features of mouse L1 sequences are well conserved in intragenic L1s. Furthermore, the significantly higher number of monomer repeats (>3 copies on average) in mouse intragenic L1s suggests their main roles in regulating transcriptional activities as reported in [8], [19]. Therefore, like human intragenic L1s, the conservation of structural features could suggest a similar transcriptional role.

### Intragenic L1s regulate genes in early embryogenesis

**Figure 4:**
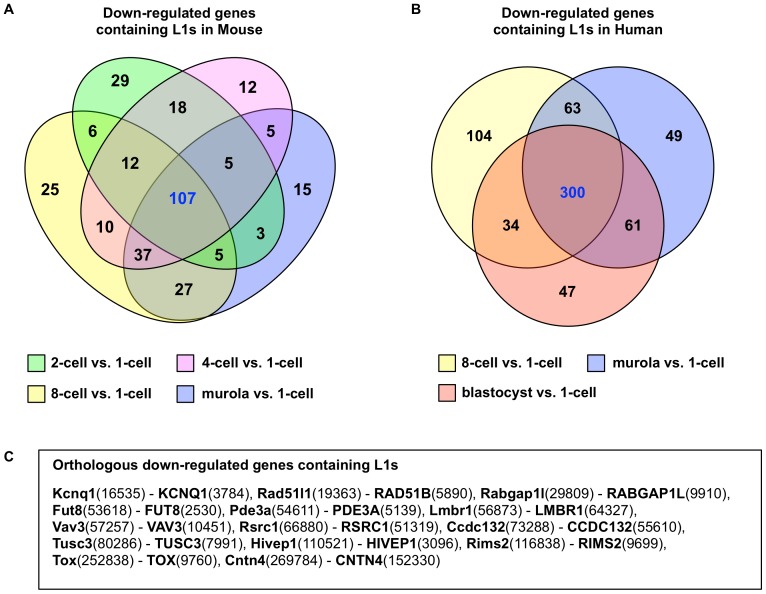
The down-regulated gene sets at differential gene expression stages in early embryogenesis that pass the chi-square tests. (A) Intersection of 4 gene sets in mouse genome. Each gene set is represented by a colored oval. The numbers in green, pink, yellow and blue ovals indicate the numbers of associated mouse genes in “2-cell vs. 1-cell”, “4-cell vs. 1-cell”, “8-cell vs. 1-cell”, and “morula vs. 1-cell” differential expressions stages, respectively. (B) Intersection of 3 gene sets in human genome. A colored circle represents each gene set. The numbers in yellow, blue, and red circles indicate the numbers of associated human genes in “8-cell vs. 1-cell”, “morula vs. 1-cell”, and “blastocyst vs. 1-cell” differential expression stages, respectively. (C) Name listing of mouse-human orthologous genes found in both mouse and human intersection gene sets. Each orthologous gene pair indicates the mouse gene name followed by the human gene name. The numbers in parentheses present the corresponding gene ids.

L1s are expressed in early embryogenesis [16], and L1 products are essential for development [30]. It is not known, however, if expression of intragenic L1 regulates expression of gene pre-mRNA in embryogenesis similar to what was reported in cancer [17]. We analyzed microarray expression data of mouse and human early embryonic stages and tested whether changes in expression are associated with intragenic L1s. In mouse, the observed numbers of genes with intragenic L1 and down-regulated relative to the one-cell stage are significantly higher than expected for all stages except blastocyst. In contrast, no significant association was found for up-regulated genes and intragenic L1s (Table 1). Significantly higher than expected numbers of down-regulated genes with intragenic L1s were also found for human embryonic stages, albeit only the latter three stages, i.e. 8-cell, morula and blastocyst (Table 2). Among the stages with significant association of intragenic L1 and down-regulation, 107 genes are commonly down regulated among mouse stages whereas 300 are common among human stages (Figure 4). Among the genes in these two intersection sets, 14 are orthologous between mouse and human, according to the mouse genome database [31]. Using Gene Ontology [32] and GeneCards [33], the molecular functions of these orthologous genes are listed in Table S3.

**Table 1:** Intragenic L1s control gene expression in mouse early embryogenesis.

Chi-square test of association between mouse intragenic L1s and differential embryo gene expression stage (between cell division stage)						
		2-cell vs.	4-cell vs.	8-cell vs.	Morula vs.	Blastocyst vs.
		1-cell	1-cell	1-cell	1-cell	1-cell
Up	p-value	9.0033E-20	7.0438E-18	1.0780E-21	1.7495E-13	1.1192E-04
	OR	0.17	0.32	0.29	0.42	0.48
	95%CI	0.11-0.26	0.24-0.42	0.23-0.38	0.33-0.53	0.32-0.70
**Down**	p-value	**6.7540E-07**	**9.3628E-09**	**1.7002E-10**	**6.5540E-07**	1.2723E-02
	OR	**1.57**	**1.65**	**1.73**	**1.55**	1.27
	95%CI	**1.31-1.87**	**1.39-1.96**	**1.46-2.05**	**1.30-1.85**	1.05-1.53

Bold items indicate differential stages that pass the threshold (OR >1.0 and p-value <1.0E-03).

**Table 2:** Intragenic L1s control gene expression in human early embryogenesis.

Chi-square test of association between human intragenic L1s and differential embryo gene expression stage (between cell division stage)						
		2-cell vs.	4-cell vs.	8-cell vs.	Morula vs.	Blastocyst vs.
		1-cell	1-cell	1-cell	1-cell	1-cell
Up	p-value	1.5217E-01	3.6563E-03	1.9267E-03	3.4991E-06	1.7847E-04
	OR	1.29	1.33	0.75	0.70	0.76
	95%CI	0.94-1.51	1.10-1.60	0.63-0.90	0.60-0.81	0.66-0.88
**Down**	p-value	8.9550E-01	8.1226E-01	**3.2387E-24**	**1.7497E-24**	**1.4097E-18**
	OR	0.98	1.02	**1.80**	**1.83**	**1.70**
	95%CI	0.72-1.34	0.85-1.24	**1.61-2.02**	**1.63-2.05**	**1.51-1.91**

Bold items indicate differential stages that pass the threshold (OR >1.0 and p-value <1.0E-03).

## Discussion

In this study, we tested for association of L1 location and sequence with respect to genes and L1 functions in two mammal species. Four main observations were made. First, L1 density is greater in mouse than human, including L1s within genes. Second, intergenic L1s density is greater in autosome and X-chromosome but less in Y-chromosome. Third, mouse intragenic L1s are less conserved than human and contain significantly more monomer repeats than that of intergenic ones. Finally, mouse and human intragenic L1s are associated with down-regulation of gene expression during early embryogenesis.

On the X-chromosome, L1 density is higher than all other chromosomes combined in mouse and human. This is consistent with the role of L1s in X-inactivation activity, where L1s are thought to act as boosters of X-inactivation chromosome spreading from a center of inactivation [12], [13]. For autosomal and X-chromosomes, intergenic L1 densities are much higher than intragenic ones. The lower density of intragenic versus intergenic L1 in both species suggests that L1 retrotransposition into genes is likely to be deleterious and would selected against in evolution [34]. This purifying selection in the X and autosomes could be facilitated by recombination of homologous chromosomes or homologous recombination DNA break repair. Y-chromosome is hemizygote and majority of the chromosome lacks homologous recombination. If the role of intergenic L1s is related to homologous recombination or homologous chromosome, intergenic L1s in Y-chromosome may have no function and can be considered as junk DNA. Rearrangements and deletion mutations of intergenic L1s in Y-chromosome should not affect fitness and the L1s should be continuously lost during evolution. In contrast to intergenic L1s, intragenic L1s possess gene regulatory function and should be conserved [14], [16], [17]. As a result, in Y-chromosome, intragenic L1 density is higher than intergenic for both mouse and human.

Intragenic L1s are more conserved than intergenic L1 for the mouse and human. Interestingly, mouse intragenic L1s are overall less conserved than human. The lower conservation of mouse L1 is particularly marked in the 5′ UTR, in which variation in monomer repeats was shown previously to control L1 promoter activity [8], [19]. The significantly higher mean number of monomer repeats in intragenic compared with intergenic L1s suggests that intragenic L1s are more transcriptionally active. The difference in mechanism of transcriptional control in human and mouse L1 may suggest that the transcriptionally active L1s have acquired biologically important functions independently in different mammalian lineages, i.e., convergent evolution [3].

The greater conservation and possible activity of intragenic L1 in mouse is suggestive of function. We investigated whether intragenic L1 might play a role in gene regulation in early embryogenesis. Significant associations were found for down regulated genes with intragenic L1 and down regulation of the genes, starting from the 2-cell to the morula stage in mouse, whereas associations were significant for 8-cell to blastocyst in human. The different “L1 associated with down regulation” (LaD) profiles align well with the varying zygotic activations and the levels of global hypomethylation among mammals [35]. In particular, mouse zygotic activation starts from 2-cell division, whereas activation starts during the 4 to 8 cell divisions in human. Furthermore, mouse embryos undergo demethylation after fertilization to become hypomethylated, and establish new methylation patterns at the blastocyst stage [36]. The mouse LaD pattern thus agrees with the global hypomethylation profile during zygotic activation (Table 1). Human embryogenesis differs from mouse in the timing of zygotic activation [35], [37] and the human LaD pattern aligns with the slower onset of activation in human (Table 2).

Although the timing of zygotic activation differs between mouse and human, intragenic L1 appears to be important for controlling gene expression in both species. Among the orthologous genes obtained from intersecting the mouse and human LaD gene sets (Table S3), two genes have previously been reported with roles in embryogenesis. *Kcnq1* was reported to be a paternally imprinted gene that is down regulated during embryogenesis development [38]. The Cyclic GMP-Inhibited Phosphodiesterase 3A (*PDE3A*) gene functions in the cGMP-PKG signaling pathway [39]. *PDE3A* must be inhibited to allow expression of other important genes during physiological development. Hence, under the global hypomethylation state during zygotic activity, intragenic L1 may be expressed which down-regulates these genes, perhaps by the same AGO2-dependent mechanism as described in cancer cells [17].

Although these tests are suggestive for possible function of intragenic L1s in mouse and human such as X-inactivation and embryogenesis, there are alternative explanations that do not require L1s to have functions. For example, some rodent species thought to lack potentially mobile L1 still have X-inactivation [40]. In addition, accumulation of L1 elements in X still continues even when X-inactivation is not needed in *Tokudaia osimensis*, an XO species [41]. The reason for conservation of intragenic L1s could stem from the genomic context that these elements are located, i.e., genic regions are likely to be more constrained by background selection, hence conservation of intragenic L1s does not necessarily imply function. Therefore, apart from direct testing for function, e.g., L1 ablation by genome editing tool, comparison among a greater range of mammalian species could provide insights into putative functions of conserved L1s. This is because a recent intragenic L1 element is unlikely to have a function and is tolerated because it has minor phenotypic consequence. On the other hand, if an intragenic L1 element has persisted for a long evolutionary time, it may have acquired a new function which can be constrained by purifying selection.

## Conclusions

We reanalyzed both mouse and human L1 data from L1base. Statistical analyses showed that mouse and human L1s are distributed similarly over their host genomes with greater density of intergenic L1s in X and autosomal chromosomes but greater density of intragenic L1s in Y chromosome. Intragenic L1s are more conserved than intergenic, and mouse intragenic L1 are more likely to be transcriptionally active owing to higher monomer repeat copy number in the 5′ UTR. Furthermore, mouse and human intragenic L1s could play a role in gene regulation during early embryogenesis as they are associated with genes down regulated during zygotic activation. Therefore, distributions of L1 in other mammalian species need to be studied to fully comprehend the functional repertoire of L1.

## Supporting Information

Table S1 **List of mouse L1 characteristics.**
(PDF)

Table S2 **List of human L1 characteristics.**
(PDF)

Table S3 **Molecular functions of 14 orthologous down-regulated genes during early embryogenesis in human and mouse.**
(PDF)
