# Supplementary material for: Roles of Intragenic and Intergenic L1s in Mouse and Human
Source: PLoS One. 2014 Nov 19;9(11):e113434. doi: 10.1371/journal.pone.0113434 (PMC4237456; doi:10.1371/journal.pone.0113434)
Supplement: Table S1 — List of mouse L1 characteristics. (PDF) [file pone.0113434.s001.pdf]

Table 1.1 Mantel-Haenszel chi-square tests for mouse L1 characteristics

Mantel-Haenszel test is calculated by performing chi-square tests on different L1 strata (grouped by mouse L1 subfamilies, F, A, TF and GF, respectively). The total number of intragenic vs. intergenic L1s are shown in each column header. The Mantel-Haenszel correction results of each mouse L1 characteristic are shown in the last column, namely MH Odds Ratio, MH sum chisq, MH p-value, and MH 95% CI, respectively.

F subfamily					A subfamily					TF subfamily					GF subfamily					Mantel-Haenszel (MH) chi-square test	
Total number of intragenic L1s = 332					Total number of intragenic L1s = 969					Total number of intragenic L1s = 909					Total number of intragenic L1s = 219						
Total number of intergenic L1s = 2270					Total number of intragenic L1s = 5367					Total number of intragenic L1s = 4031					Total number of intragenic L1s = 1403						
**Overall**																					
**ORF StartStop**																					
Case	Intragenic L1s		Intergenic L1s		Case	Intragenic L1s		Intergenic L1s		Case	Intragenic L1s		Intergenic L1s		Case	Intragenic L1s		Intergenic L1s			
	Observed	Expected	Observed	Expected		Observed	Expected	Observed	Expected		Observed	Expected	Observed	Expected		Observed	Expected	Observed	Expected		
ORF1 Conserved	74	82.3	571	562.7	ORF1 Conserved	125	119.44	656	661.56	ORF1 Conserved	13	26.13	129	115.87	ORF1 Conserved	15	15.39	99	98.61	MH Odds Ratio	0.91
other	258	249.7	1699	1707.3	other	844	849.56	4711	4705.44	other	896	882.87	3902	3915.13	other	204	203.61	1304	1304.39	MH sum chisq	1.50
Odds Ratio = 0.85, 95% CI = 0.65 - 1.12					Odds Ratio = 1.06, 95% CI = 0.87 - 1.31					Odds Ratio = 0.44, 95% CI = 0.25 - 0.78					Odds Ratio = 0.97, 95% CI = 0.55 - 1.70					MH p-value	2.20E-01
Sum chisq = 1.27520, p-value = 2.59E-01					Sum chisq = 0.34816, p-value = 5.55E-01					Sum chisq = 8.32394, p-value = 3.91E-03					Sum chisq = 0.01242, p-value = 9.11E-01					MH 95% CI	0.78-1.06
ORF2 Conserved	61	63.29	435	432.71	ORF2 Conserved	43	68.67	406	380.33	ORF2 Conserved	54	70.11	327	310.89	ORF2 Conserved	21	25.38	167	162.62	MH Odds Ratio	0.73
other	271	268.71	1835	1837.29	other	926	900.33	4961	4986.67	other	855	838.89	3704	3720.11	other	198	193.62	1236	1240.38	MH sum chisq	13.73
Odds Ratio = 0.95, 95% CI = 0.71 - 1.28					Odds Ratio = 0.57, 95% CI = 0.41 - 0.78					Odds Ratio = 0.72, 95% CI = 0.53 - 0.96					Odds Ratio = 0.78, 95% CI = 0.49 - 1.27					MH p-value	2.11E-04
Sum chisq = 0.11701, p-value = 7.32E-01					Sum chisq = 12.19093, p-value = 4.80E-04					Sum chisq = 4.91409, p-value = 2.66E-02					Sum chisq = 0.98988, p-value = 3.20E-01					MH 95% CI	0.62-0.87
Conserved	130	127.21	867	869.79	Conserved	728	711.76	3926	3942.24	Conserved	841	804.3	3530	3566.7	Conserved	181	174.17	1109	1115.83	MH Odds Ratio	1.21
other	202	204.79	1403	1400.21	other	241	257.24	1441	1424.76	other	68	104.7	501	464.3	other	38	44.83	294	287.17	MH sum chisq	11.68
Odds Ratio = 1.04, 95% CI = 0.82 - 1.32					Odds Ratio = 1.11, 95% CI = 0.95 - 1.30					Odds Ratio = 1.76, 95% CI = 1.35 - 2.29					Odds Ratio = 1.26, 95% CI = 0.87 - 1.83					MH p-value	6.31E-04
Sum chisq = 0.11360, p-value = 7.36E-01					Sum chisq = 1.64735, p-value = 1.99E-01					Sum chisq = 17.81794, p-value = 2.43E-05					Sum chisq = 1.51103, p-value = 2.19E-01					MH 95% CI	1.09-1.36
Mutated	67	59.2	397	404.8	Mutated	73	69.13	379	382.87	Mutated	1	8.46	45	37.54	Mutated	2	4.05	28	25.95	MH Odds Ratio	1.02
other	265	272.8	1873	1865.2	other	896	899.87	4988	4984.13	other	908	900.54	3986	3993.46	other	217	214.95	1375	1377.05	MH sum chisq	0.04
Odds Ratio = 1.19, 95% CI = 0.89 - 1.59					Odds Ratio = 1.07, 95% CI = 0.83 - 1.39					Odds Ratio = 0.10, 95% CI = 0.01 - 0.71					Odds Ratio = 0.45, 95% CI = 0.11 - 1.91					MH p-value	8.35E-01
Sum chisq = 1.43222, p-value = 2.31E-01					Sum chisq = 0.27587, p-value = 5.99E-01					Sum chisq = 8.14271, p-value = 4.32E-03					Sum chisq = 0.69913, p-value = 4.03E-01					MH 95% CI	0.81-1.29
**CPG Islands**																					
Case	Intragenic L1s		Intergenic L1s		Case	Intragenic L1s		Intergenic L1s		Case	Intragenic L1s		Intergenic L1s		Case	Intragenic L1s		Intergenic L1s			
	Observed	Expected	Observed	Expected		Observed	Expected	Observed	Expected		Observed	Expected	Observed	Expected		Observed	Expected	Observed	Expected		
0 island	327	325.62	2225	2226.38	0 island	956	951.41	5265	5269.59	0 island	814	823.07	3659	3649.93	0 island	185	193.21	1246	1237.79	MH Odds Ratio	0.90
other	5	6.38	45	43.62	other	13	17.59	102	97.41	other	95	85.93	372	381.07	other	34	25.79	157	165.21	MH sum chisq	1.24
Odds Ratio = 1.32, 95% CI = 0.52 - 3.36					Odds Ratio = 1.42, 95% CI = 0.80 - 2.55					Odds Ratio = 0.87, 95% CI = 0.69 - 1.10					Odds Ratio = 0.69, 95% CI = 0.46 - 1.02					MH p-value	2.66E-01
Sum chisq = 0.34872, p-value = 5.55E-01					Sum chisq = 1.43881, p-value = 2.30E-01					Sum chisq = 1.29519, p-value = 2.55E-01					Sum chisq = 3.42625, p-value = 6.42E-02					MH 95% CI	0.74-1.09
>=1 island(s)	5	6.38	45	43.62	>=1 island(s)	13	17.59	102	97.41	>=1 island(s)	95	85.93	372	381.07	>=1 island(s)	34	25.79	157	165.21	MH Odds Ratio	1.11
other	327	325.62	2225	2226.38	other	956	951.41	5265	5269.59	other	814	823.07	3659	3649.93	other	185	193.21	1246	1237.79	MH sum chisq	1.24
Odds Ratio = 0.76, 95% CI = 0.30 - 1.92					Odds Ratio = 0.70, 95% CI = 0.39 - 1.26					Odds Ratio = 1.15, 95% CI = 0.91 - 1.46					Odds Ratio = 1.46, 95% CI = 0.98 - 2.18					MH p-value	2.66E-01
Sum chisq = 0.34872, p-value = 5.55E-01					Sum chisq = 1.43881, p-value = 2.30E-01					Sum chisq = 1.29519, p-value = 2.55E-01					Sum chisq = 3.42625, p-value = 6.42E-02					MH 95% CI	0.92-1.35
**5' UTR**																					
**SA-154**																					
Case	Intragenic L1s		Intergenic L1s		Case	Intragenic L1s		Intergenic L1s		Case	Intragenic L1s		Intergenic L1s		Case	Intragenic L1s		Intergenic L1s			
	Observed	Expected	Observed	Expected		Observed	Expected	Observed	Expected		Observed	Expected	Observed	Expected		Observed	Expected	Observed	Expected		
Conserved	283	285.43	1954	1951.57	Conserved	458	548.27	3127	3036.73	Conserved	906	904.03	4007	4008.97	Conserved	199	200.1	1283	1281.9	MH Odds Ratio	0.70
other	49	46.57	316	318.43	other	511	420.73	2240	2330.27	other	3	4.97	24	22.03	other	20	18.9	120	121.1	MH sum chisq	32.99
Odds Ratio = 0.93, 95% CI = 0.67 - 1.29					Odds Ratio = 0.64, 95% CI = 0.56 - 0.74					Odds Ratio = 1.81, 95% CI = 0.54 - 6.02					Odds Ratio = 0.93, 95% CI = 0.57 - 1.53					MH p-value	9.26E-09
Sum chisq = 0.16879, p-value = 6.81E-01					Sum chisq = 40.41450, p-value = 2.05E-10					Sum chisq = 0.53466, p-value = 4.65E-01					Sum chisq = 0.08061, p-value = 7.76E-01					MH 95% CI	0.62-0.79
Mutated	49	46.57	316	318.43	Mutated	511	420.73	2240	2330.27	Mutated	3	4.97	24	22.03	Mutated	20	18.9	120	121.1	MH Odds Ratio	1.43
other	283	285.43	1954	1951.57	other	458	548.27	3127	3036.73	other	906	904.03	4007	4008.97	other	199	200.1	1283	1281.9	MH sum chisq	32.99
Odds Ratio = 1.07, 95% CI = 0.77 - 1.48					Odds Ratio = 1.56, 95% CI = 1.36 - 1.79					Odds Ratio = 0.55, 95% CI = 0.17 - 1.84					Odds Ratio = 1.07, 95% CI = 0.65 - 1.77					MH p-value	9.26E-09
Sum chisq = 0.16879, p-value = 6.81E-01					Sum chisq = 40.41450, p-value = 2.05E-10					Sum chisq = 0.53466, p-value = 4.65E-01					Sum chisq = 0.08061, p-value = 7.76E-01					MH 95% CI	1.26-1.61
**ORF1**																					
**ORF1 Conserved**																					
Case	Intragenic L1s		Intergenic L1s		Case	Intragenic L1s		Intergenic L1s		Case	Intragenic L1s		Intergenic L1s		Case	Intragenic L1s		Intergenic L1s			
	Observed	Expected	Observed	Expected		Observed	Expected	Observed	Expected		Observed	Expected	Observed	Expected		Observed	Expected	Observed	Expected		
Conserved	332	332	2270	2270	Conserved	969	969	5367	5367	Conserved	909	909	4031	4031	Conserved	219	219	1403	1403	MH Odds Ratio	-
other	0	0	0	0	other	0	0	0	0	other	0	0	0	0	other	0	0	0	0	MH sum chisq	-
Odds Ratio = -, 95% CI = 0.00 - inf					Odds Ratio = -, 95% CI = 0.00 - inf					Odds Ratio = -, 95% CI = 0.00 - inf					Odds Ratio = -, 95% CI = 0.00 - inf					MH p-value	-
Sum chisq = -, p-value = -					Sum chisq = -, p-value = -					Sum chisq = -, p-value = -					Sum chisq = -, p-value = -					MH 95% CI	-
**66/42 Monomers**																					
Case	Intragenic L1s		Intergenic L1s		Case	Intragenic L1s		Intergenic L1s		Case	Intragenic L1s		Intergenic L1s		Case	Intragenic L1s		Intergenic L1s			
	Observed	Expected	Observed	Expected		Observed	Expected	Observed	Expected		Observed	Expected	Observed	Expected		Observed	Expected	Observed	Expected		
66-42	129	141.12	977	964.88	66-42	414	364.29	1968	2017.71	66-42	0	2.39	13	10.61	66-42	11	12.96	85	83.04	MH Odds Ratio	1.13
other	203	190.88	1293	1305.12	other	555	604.71	3399	3349.29	other	909	906.61	4018	4020.39	other	208	206.04	1318	1319.96	MH sum chisq	4.00
Odds Ratio = 0.84, 95% CI = 0.66 - 1.06					Odds Ratio = 1.29, 95% CI = 1.12 - 1.48					Odds Ratio = 0.00, 95% CI = -					Odds Ratio = 0.82, 95% CI = 0.43 - 1.56					MH p-value	4.54E-02
Sum chisq = 2.07498, p-value = 1.50E-01					Sum chisq = 12.83080, p-value = 3.41E-04					Sum chisq = 1.83895, p-value = 1.75E-01					Sum chisq = 0.36486, p-value = 5.46E-01					MH 95% CI	-
66-42-42	141	130.27	880	890.73	66-42-42	337	386.93	2193	2143.07	66-42-42	907	903.66	4004	4007.34	66-42-42	84	116.66	780	747.34	MH Odds Ratio	0.80
other	191	201.73	1390	1379.27	other	632	582.07	3174	3223.93	other	2	5.34	27	23.66	other	135	102.34	623	655.66	MH sum chisq	14.79
Odds Ratio = 1.17, 95% CI = 0.92 - 1.47					Odds Ratio = 0.77, 95% CI = 0.67 - 0.89					Odds Ratio = 3.06, 95% CI = 0.73 - 12.88					Odds Ratio = 0.50, 95% CI = 0.37 - 0.67					MH p-value	1.20E-04
Sum chisq = 1.66612, p-value = 1.97E-01					Sum chisq = 12.66115, p-value = 3.73E-04					Sum chisq = 2.57128, p-value = 1.09E-01					Sum chisq = 22.61483, p-value = 1.98E-06					MH 95% CI	0.72-0.90
66-42-42-42	2	3.83	28	26.17	66-42-42-42	93	97.42	544	539.58	66-42-42-42	1	0.37	1	1.63	66-42-42-42	116	76.42	450	489.58	MH Odds Ratio	1.32
other	330	328.17	2242	2243.83	other	876	871.58	4823	4827.42	other	908	908.63	4030	4029.37	other	103	142.58	953	913.42	MH sum chisq	9.54
Odds Ratio = 0.49, 95% CI = 0.12 - 2.05					Odds Ratio = 0.94, 95% CI = 0.75 - 1.19					Odds Ratio = 4.44, 95% CI = 0.28 - 71.03					Odds Ratio = 2.39, 95% CI = 1.79 - 3.18					MH p-value	2.01E-03
Sum chisq = 0.53413, p-value = 4.65E-01					Sum chisq = 0.26320, p-value = 6.08E-01					Sum chisq = 0.05803, p-value = 8.10E-01					Sum chisq = 36.40087, p-value = 1.61E-09					MH 95% CI	1.09-1.58
**SD+29**																					
Case	Intragenic L1s		Intergenic L1s		Case	Intragenic L1s		Intergenic L1s		Case	Intragenic L1s		Intergenic L1s		Case	Intragenic L1s		Intergenic L1s			
	Observed	Expected	Observed	Expected		Observed	Expected	Observed	Expected		Observed	Expected	Observed	Expected		Observed	Expected	Observed	Expected		
Conserved	294	292.32	1997	1998.68	Conserved	879	879.23	4870	4869.77	Conserved	902	896.67	3971	3976.33	Conserved	217	214.27	1370	1372.73	MH Odds Ratio	1.09
other	38	39.68	273	271.32	other	90	89.77	497	497.23	other	7	12.33	60	54.67	other	2	4.73	33	30.27	MH sum chisq	0.80
Odds Ratio = 1.06, 95% CI = 0.74 - 1.52					Odds Ratio = 1.00, 95% CI = 0.79 - 1.26					Odds Ratio = 1.95, 95% CI = 0.89 - 4.27					Odds Ratio = 2.61, 95% CI = 0.62 - 10.97					MH p-value	3.72E-01
Sum chisq = 0.09279, p-value = 7.61E-01					Sum chisq = 0.00075, p-value = 9.78E-01					Sum chisq = 2.86121, p-value = 9.07E-02					Sum chisq = 1.23856, p-value = 2.66E-01					MH 95% CI	1.32-1.37
Mutated	38	39.68	273	271.32	Mutated	90	89.77	497	497.23	Mutated	7	12.33	60	54.67	Mutated	2	4.73	33	30.27	MH Odds Ratio	0.92
other	294	292.32	1997	1998.68	other	879	879.23	4870	4869.77	other	902	896.67	3971	3976.33	other	217	214.27	1370	1372.73	MH sum chisq	0.80
Odds Ratio = 0.95, 95% CI = 0.66 - 1.36					Odds Ratio = 1.00, 95% CI = 0.79 - 1.27					Odds Ratio = 0.51, 95% CI = 0.23 - 1.13					Odds Ratio = 0.38, 95% CI = 0.09 - 1.61					MH p-value	3.72E-01
Sum chisq = 0.09279, p-value = 7.61E-01					Sum chisq = 0.00075, p-value = 9.78E-01					Sum chisq = 2.86121, p-value = 9.07E-02					Sum chisq = 1.23856, p-value = 2.66E-01					MH 95% CI	0.76-1.11
**SD+52**																					
Case	Intragenic L1s		Intergenic L1s		Case	Intragenic L1s		Intergenic L1s		Case	Intragenic L1s		Intergenic L1s		Case	Intragenic L1s		Intergenic L1s			
	Observed	Expected	Observed	Expected		Observed	Expected	Observed	Expected		Observed	Expected	Observed	Expected		Observed	Expected	Observed	Expected		
Conserved	275	283.13	1944	1935.87	Conserved	830	853.69	4752	4728.31	Conserved	907	902.74	3999	4003.26	Conserved	216	214.81	1375	1376.19	MH Odds Ratio	0.83
other	57	48.87	326	334.13	other	139	115.31	615	638.69	other	2	6.26	32	27.74	other	3	4.19	28	26.81	MH sum chisq	5.31
Odds Ratio = 0.81, 95% CI = 0.59 - 1.10					Odds Ratio = 0.77, 95% CI = 0.63 - 0.94					Odds Ratio = 3.63, 95% CI = 0.87 - 15.17					Odds Ratio = 1.47, 95% CI = 0.44 - 4.86					MH p-value	2.13E-02
Sum chisq = 1.81860, p-value = 1.77E-01					Sum chisq = 6.51978, p-value = 1.07E-02					Sum chisq = 3.57320, p-value = 5.87E-02					Sum chisq = 0.13235, p-value = 7.16E-01					MH 95% CI	0.70-0.97
Mutated	57	48.87	326	334.13	Mutated	139	115.31	615	638.69	Mutated	2	6.26	32	27.74	Mutated	3	4.19	28	26.81	MH Odds Ratio	1.21
other	275	283.13	1944	1935.87	other	830	853.69	4752	4728.31	other	907	902.74	3999	4003.26	other	216	214.81	1375	1376.19	MH sum chisq	5.31
Odds Ratio = 1.24, 95% CI = 0.91 - 1.68					Odds Ratio = 1.29, 95% CI = 1.06 - 1.58					Odds Ratio = 0.28, 95% CI = 0.07 - 1.15					Odds Ratio = 0.68, 95% CI = 0.21 - 2.26					MH p-value	2.13E-02
Sum chisq = 1.81860, p-value = 1.77E-01					Sum chisq = 6.51978, p-value = 1.07E-02					Sum chisq = 3.57320, p-value = 5.87E-02					Sum chisq = 0.13235, p-value = 7.16E-01					MH 95% CI	1.02-1.44
**SD+106**																					
Case	Intragenic L1s		Intergenic L1s		Case	Intragenic L1s		Intergenic L1s		Case	Intragenic L1s		Intergenic L1s		Case	Intragenic L1s		Intergenic L1s			
	Observed	Expected	Observed	Expected		Observed	Expected	Observed	Expected		Observed	Expected	Observed	Expected		Observed	Expected	Observed	Expected		
Conserved	269	265.27	1810	1813.73	Conserved	202	210.29	1173	1164.71	Conserved	905	899.06	3981	3986.94	Conserved	212	211.17	1352	1352.83	MH Odds Ratio	1.01
other	63	66.73	460	456.27	other	767	758.71	4194	4202.29	other	4	9.94	50	44.06	other	7	7.83	51	50.17	MH sum chisq	0.02
Odds Ratio = 1.09, 95% CI = 0.81 - 1.45					Odds Ratio = 0.94, 95% CI = 0.80 - 1.11					Odds Ratio = 2.84, 95% CI = 1.02 - 7.89					Odds Ratio = 1.14, 95% CI = 0.51 - 2.55					MH p-value	8.76E-01
Sum chisq = 0.29938, p-value = 5.84E-01					Sum chisq = 0.49233, p-value = 4.83E-01					Sum chisq = 4.39449, p-value = 3.61E-02					Sum chisq = 0.10575, p-value = 7.45E-01					MH 95% CI	0.88-1.17
Mutated	63	66.73	460	456.27	Mutated	767	758.71	4194	4202.29	Mutated	4	9.94	50	44.06	Mutated	7	7.83	51	50.17	MH Odds Ratio	0.99
other	269	265.27	1810	1813.73	other	202	210.29	1173	1164.71	other	905	899.06	3981	3986.94	other	212	211.17	1352	1352.83	MH sum chisq	0.02
Odds Ratio = 0.92, 95% CI = 0.69 - 1.24					Odds Ratio = 1.06, 95% CI = 0.90 - 1.26					Odds Ratio = 0.35, 95% CI = 0.13 - 0.98					Odds Ratio = 0.88, 95% CI = 0.39 - 1.95					MH p-value	8.76E-01
Sum chisq = 0.29938, p-value = 5.84E-01					Sum chisq = 0.49233, p-value = 4.83E-01					Sum chisq = 4.39449, p-value = 3.61E-02					Sum chisq = 0.10575, p-value = 7.45E-01					MH 95% CI	0.86-1.14
**SA+106**																					
Case	Intragenic L1s		Intergenic L1s		Case	Intragenic L1s		Intergenic L1s		Case	Intragenic L1s		Intergenic L1s		Case	Intragenic L1s		Intergenic L1s			
	Observed	Expected	Observed	Expected		Observed	Expected	Observed	Expected		Observed	Expected	Observed	Expected		Observed	Expected	Observed	Expected		
Conserved	70	71.58	491	489.42	Conserved	71	80.14	453	443.86	Conserved	648	578.52	2496	2565.48	Conserved	216	210.76	1345	1350.24	MH Odds Ratio	1.26
other	262	260.42	1779	1780.58	other	898	888.86	4914	4923.14	other	261	330.48	1535	1465.52	other	3	8.24	58	52.76	MH sum chisq	14.13
Odds Ratio = 0.97, 95% CI = 0.73 - 1.28					Odds Ratio = 0.86, 95% CI = 0.66 - 1.11					Odds Ratio = 1.53, 95% CI = 1.30 - 1.79					Odds Ratio = 3.10, 95% CI = 0.96 - 10.00					MH p-value	1.70E-04
Sum chisq = 0.05099, p-value = 8.21E-01					Sum chisq = 1.34110, p-value = 2.47E-01					Sum chisq = 28.12656, p-value = 1.14E-07					Sum chisq = 3.99888, p-value = 4.55E-02					MH 95% CI	1.11-1.42
Mutated	262	260.42	1779	1780.58	Mutated	898	888.86	4914	4923.14	Mutated	261	330.48	1535	1465.52	Mutated	3	8.24	58	52.76	MH Odds Ratio	0.79
other	70	71.58	491	489.42	other	71	80.14	453	443.86	other	648	578.52	2496	2565.48	other	216	210.76	1345	1350.24	MH sum chisq	14.13
Odds Ratio = 1.03, 95% CI = 0.78 - 1.37					Odds Ratio = 1.17, 95% CI = 0.90 - 1.51					Odds Ratio = 0.65, 95% CI = 0.56 - 0.77					Odds Ratio = 0.32, 95% CI = 0.10 - 1.04					MH p-value	1.70E-04
Sum chisq = 0.05099, p-value = 8.21E-01					Sum chisq = 1.34110, p-value = 2.47E-01					Sum chisq = 28.12656, p-value = 1.14E-07					Sum chisq = 3.99888, p-value = 4.55E-02					MH 95% CI	0.70-0.90
**SA+120**																					
Case	Intragenic L1s		Intergenic L1s		Case	Intragenic L1s		Intergenic L1s		Case	Intragenic L1s		Intergenic L1s		Case	Intragenic L1s		Intergenic L1s			
	Observed	Expected	Observed	Expected		Observed	Expected	Observed	Expected		Observed	Expected	Observed	Expected		Observed	Expected	Observed	Expected		
Conserved	63	68.77	476	470.23	Conserved	30	30.43	169	168.57	Conserved	873	847.54	3733	3758.46	Conserved	217	212.11	1354	1358.89	MH Odds Ratio	1.23
other	269	263.23	1794	1799.77	other	939	938.57	5198	5198.43	other	36	61.46	298	272.54	other	2	6.89	49	44.11	MH sum chisq	4.66
Odds Ratio = 0.88, 95% CI = 0.66 - 1.18					Odds Ratio = 0.98, 95% CI = 0.66 - 1.46					Odds Ratio = 1.94, 95% CI = 1.36 - 2.76					Odds Ratio = 3.93, 95% CI = 0.95 - 16.26					MH p-value	3.08E-02
Sum chisq = 0.70067, p-value = 4.03E-01					Sum chisq = 0.00755, p-value = 9.31E-01					Sum chisq = 13.86138, p-value = 1.97E-04					Sum chisq = 4.13810, p-value = 4.19E-02					MH 95% CI	1.02-1.50
Mutated	269	263.23	1794	1799.77	Mutated	939	938.57	5198	5198.43	Mutated	36	61.46	298	272.54	Mutated	2	6.89	49	44.11	MH Odds Ratio	0.81
other	63	68.77	476	470.23	other	30	30.43	169	168.57	other	873	847.54	3733	3758.46	other	217	212.11	1354	1358.89	MH sum chisq	4.66
Odds Ratio = 1.13, 95% CI = 0.85 - 1.52					Odds Ratio = 1.02, 95% CI = 0.69 - 1.51					Odds Ratio = 0.52, 95% CI = 0.36 - 0.74					Odds Ratio = 0.25, 95% CI = 0.06 - 1.05					MH p-value	3.08E-02
Sum chisq = 0.70067, p-value = 4.03E-01					Sum chisq = 0.00755, p-value = 9.31E-01					Sum chisq = 13.86138, p-value = 1.97E-04					Sum chisq = 4.13810, p-value = 4.19E-02					MH 95% CI	0.66-1.00
**REKG235**																					
Case	Intragenic L1s		Intergenic L1s		Case	Intragenic L1s		Intergenic L1s		Case	Intragenic L1s		Intergenic L1s		Case	Intragenic L1s		Intergenic L1s			
	Observed	Expected	Observed	Expected		Observed	Expected	Observed	Expected		Observed	Expected	Observed	Expected		Observed	Expected	Observed	Expected		
Conserved	216	213.08	1454	1456.92	Conserved	851	826.01	4550	4574.99	Conserved	897	883.42	3904	3917.58	Conserved	204	195.78	1246	1254.22	MH Odds Ratio	1.29
other	116	118.92	816	813.08	other	118	142.99	817	792.01	other	12	25.58	127	113.42	other	15	23.22	157	148.78	MH sum chisq	11.87
Odds Ratio = 1.05, 95% CI = 0.82 - 1.33					Odds Ratio = 1.29, 95% CI = 1.05 - 1.59					Odds Ratio = 2.43, 95% CI = 1.34 - 4.41					Odds Ratio = 1.71, 95% CI = 0.99 - 2.97					MH p-value	5.70E-04
Sum chisq = 0.12786, p-value = 7.21E-01					Sum chisq = 6.05067, p-value = 1.39E-02					Sum chisq = 9.08810, p-value = 2.57E-03					Sum chisq = 3.76560, p-value = 5.23E-02					MH 95% CI	1.12-1.49
Mutated	116	118.92	816	813.08	Mutated	118	142.99	817	792.01	Mutated	12	25.58	127	113.42	Mutated	15	23.22	157	148.78	MH Odds Ratio	0.77
other	216	213.08	1454	1456.92	other	851	826.01	4550	4574.99	other	897	883.42	3904	3917.58	other	204	195.78	1246	1254.22	MH sum chisq	11.87
Odds Ratio = 0.96, 95% CI = 0.75 - 1.22					Odds Ratio = 0.77, 95% CI = 0.63 - 0.95					Odds Ratio = 0.41, 95% CI = 0.23 - 0.75					Odds Ratio = 0.58, 95% CI = 0.34 - 1.01					MH p-value	5.70E-04
Sum chisq = 0.12786, p-value = 7.21E-01					Sum chisq = 6.05067, p-value = 1.39E-02					Sum chisq = 3.76560, p-value = 5.23E-02					Sum chisq = 3.76560, p-value = 5.23E-02					MH 95% CI	0.67-0.90

ARR260

Case	Intragenic L1s		Intergenic L1s		Case	Intragenic L1s		Intergenic L1s		Case	Intragenic L1s		Intergenic L1s		Case	Intragenic L1s		Intergenic L1s			
	Observed	Expected	Observed	Expected		Observed	Expected	Observed	Expected		Observed	Expected	Observed	Expected		Observed	Expected	Observed	Expected		
Conserved	260	256.59	1751	1754.41	Conserved	885	871.27	4812	4825.73	Conserved	896	891.89	3951	3955.11	Conserved	211	208.06	1330	1332.94	MH Odds Ratio	1.19
other	72	75.41	519	515.59	other	84	97.73	555	541.27	other	13	17.11	80	75.89	other	8	10.94	73	70.06	MH sum chisq	3.95
Odds Ratio = 1.07, 95% CI = 0.81 - 1.41					Odds Ratio = 1.22, 95% CI = 0.96 - 1.55					Odds Ratio = 1.40, 95% CI = 0.77 - 2.52					Odds Ratio = 1.45, 95% CI = 0.69 - 3.05					MH p-value	4.68E-02
Sum chisq = 0.22845, p-value = 6.33E-01					Sum chisq = 2.53117, p-value = 1.12E-01					Sum chisq = 1.23456, p-value = 2.67E-01					Sum chisq = 0.95945, p-value = 3.27E-01					MH 95% CI	1.0-1.41
Mutated	72	75.41	519	515.59	Mutated	84	97.73	555	541.27	Mutated	13	17.11	80	75.89	Mutated	8	10.94	73	70.06	MH Odds Ratio	0.84
other	260	256.59	1751	1754.41	other	885	871.27	4812	4825.73	other	896	891.89	3951	3955.11	other	211	208.06	1330	1332.94	MH sum chisq	3.95
Odds Ratio = 0.93, 95% CI = 0.71 - 1.23					Odds Ratio = 0.82, 95% CI = 0.65 - 1.05					Odds Ratio = 0.72, 95% CI = 0.40 - 1.29					Odds Ratio = 0.69, 95% CI = 0.33 - 1.45					MH p-value	4.68E-02
Sum chisq = 0.22845, p-value = 6.33E-01					Sum chisq = 2.53117, p-value = 1.12E-01					Sum chisq = 1.23456, p-value = 2.67E-01					Sum chisq = 0.95945, p-value = 3.27E-01					MH 95% CI	0.71-1.00

YPAKLS282

Case	Intragenic L1s		Intergenic L1s		Case	Intragenic L1s		Intergenic L1s		Case	Intragenic L1s		Intergenic L1s		Case	Intragenic L1s		Intergenic L1s			
	Observed	Expected	Observed	Expected		Observed	Expected	Observed	Expected		Observed	Expected	Observed	Expected		Observed	Expected	Observed	Expected		
Conserved	206	195.86	1329	1339.14	Conserved	796	761.47	4183	4217.53	Conserved	893	869.62	3833	3856.38	Conserved	202	193.35	1230	1238.65	MH Odds Ratio	1.38
other	126	136.14	941	930.86	other	173	207.53	1184	1149.47	other	16	39.38	198	174.62	other	17	25.65	173	164.35	MH sum chisq	22.75
Odds Ratio = 1.16, 95% CI = 0.91 - 1.47					Odds Ratio = 1.30, 95% CI = 1.09 - 1.55					Odds Ratio = 2.88, 95% CI = 1.72 - 4.82					Odds Ratio = 1.67, 95% CI = 0.99 - 2.81					MH p-value	1.85E-06
Sum chisq = 1.46830, p-value = 2.26E-01					Sum chisq = 8.63281, p-value = 3.30E-03					Sum chisq = 17.77876, p-value = 2.48E-05					Sum chisq = 3.82243, p-value = 5.06E-02					MH 95% CI	1.21-1.57
Mutated	126	136.14	941	930.86	Mutated	173	207.53	1184	1149.47	Mutated	16	39.38	198	174.62	Mutated	17	25.65	173	164.35	MH Odds Ratio	0.73
other	206	195.86	1329	1339.14	other	796	761.47	4183	4217.53	other	893	869.62	3833	3856.38	other	202	193.35	1230	1238.65	MH sum chisq	22.75
Odds Ratio = 0.86, 95% CI = 0.68 - 1.09					Odds Ratio = 0.77, 95% CI = 0.64 - 0.92					Odds Ratio = 0.35, 95% CI = 0.21 - 0.58					Odds Ratio = 0.60, 95% CI = 0.36 - 1.01					MH p-value	1.85E-06
Sum chisq = 1.46830, p-value = 2.26E-01					Sum chisq = 8.63281, p-value = 3.30E-03					Sum chisq = 17.77876, p-value = 2.48E-05					Sum chisq = 3.82243, p-value = 5.06E-02					MH 95% CI	0.63-0.83

SD+288

Case	Intragenic L1s		Intergenic L1s		Case	Intragenic L1s		Intergenic L1s		Case	Intragenic L1s		Intergenic L1s		Case	Intragenic L1s		Intergenic L1s			
	Observed	Expected	Observed	Expected		Observed	Expected	Observed	Expected		Observed	Expected	Observed	Expected		Observed	Expected	Observed	Expected		
Conserved	272	279.43	1918	1910.57	Conserved	150	187.65	1077	1039.35	Conserved	899	895.75	3969	3972.25	Conserved	217	212.65	1358	1362.35	MH Odds Ratio	0.81
other	60	52.57	352	359.43	other	819	781.35	4290	4327.65	other	10	13.25	62	58.75	other	2	6.35	45	40.65	MH sum chisq	7.68
Odds Ratio = 0.83, 95% CI = 0.62 - 1.13					Odds Ratio = 0.73, 95% CI = 0.61 - 0.88					Odds Ratio = 1.40, 95% CI = 0.72 - 2.75					Odds Ratio = 3.60, 95% CI = 0.87 - 14.93					MH p-value	5.57E-03
Sum chisq = 1.43066, p-value = 2.32E-01					Sum chisq = 11.06077, p-value = 8.82E-04					Sum chisq = 0.99062, p-value = 3.20E-01					Sum chisq = 3.54344, p-value = 5.98E-02					MH 95% CI	0.69-0.94
Mutated	60	52.57	352	359.43	Mutated	819	781.35	4290	4327.65	Mutated	10	13.25	62	58.75	Mutated	2	6.35	45	40.65	MH Odds Ratio	1.24
other	272	279.43	1918	1910.57	other	150	187.65	1077	1039.35	other	899	895.75	3969	3972.25	other	217	212.65	1358	1362.35	MH sum chisq	7.68
Odds Ratio = 1.20, 95% CI = 0.89 - 1.63					Odds Ratio = 1.37, 95% CI = 1.14 - 1.65					Odds Ratio = 0.71, 95% CI = 0.36 - 1.39					Odds Ratio = 0.28, 95% CI = 0.07 - 1.15					MH p-value	5.57E-03
Sum chisq = 1.43066, p-value = 2.32E-01					Sum chisq = 11.06077, p-value = 8.82E-04					Sum chisq = 0.99062, p-value = 3.20E-01					Sum chisq = 3.54344, p-value = 5.98E-02					MH 95% CI	1.06-1.45

SD+350

Case	Intragenic L1s		Intergenic L1s		Case	Intragenic L1s		Intergenic L1s		Case	Intragenic L1s		Intergenic L1s		Case	Intragenic L1s		Intergenic L1s			
	Observed	Expected	Observed	Expected		Observed	Expected	Observed	Expected		Observed	Expected	Observed	Expected		Observed	Expected	Observed	Expected		
Conserved	265	262.97	1796	1798.03	Conserved	839	833.5	4611	4616.5	Conserved	900	893.36	3955	3961.64	Conserved	213	209.82	1341	1344.18	MH Odds Ratio	1.11
other	67	69.03	474	471.97	other	130	135.5	756	750.5	other	9	15.64	76	69.36	other	6	9.18	62	58.82	MH sum chisq	1.81
Odds Ratio = 1.04, 95% CI = 0.78 - 1.39					Odds Ratio = 1.06, 95% CI = 0.87 - 1.29					Odds Ratio = 1.92, 95% CI = 0.96 - 3.85					Odds Ratio = 1.64, 95% CI = 0.70 - 3.84					MH p-value	1.79E-01
Sum chisq = 0.08626, p-value = 7.69E-01					Sum chisq = 0.30650, p-value = 5.80E-01					Sum chisq = 3.51578, p-value = 6.08E-02					Sum chisq = 1.33011, p-value = 2.49E-01					MH 95% CI	0.95-1.30
Mutated	67	69.03	474	471.97	Mutated	130	135.5	756	750.5	Mutated	9	15.64	76	69.36	Mutated	6	9.18	62	58.82	MH Odds Ratio	0.90
other	265	262.97	1796	1798.03	other	839	833.5	4611	4616.5	other	900	893.36	3955	3961.64	other	213	209.82	1341	1344.18	MH sum chisq	1.81
Odds Ratio = 0.96, 95% CI = 0.72 - 1.28					Odds Ratio = 0.95, 95% CI = 0.77 - 1.15					Odds Ratio = 0.52, 95% CI = 0.26 - 1.04					Odds Ratio = 0.61, 95% CI = 0.26 - 1.43					MH p-value	1.79E-01
Sum chisq = 0.08626, p-value = 7.69E-01					Sum chisq = 0.30650, p-value = 5.80E-01					Sum chisq = 3.51578, p-value = 6.08E-02					Sum chisq = 1.33011, p-value = 2.49E-01					MH 95% CI	0.77-1.05

ORF2

ORF2 Conserved

Case	Intragenic L1s		Intergenic L1s		Case	Intragenic L1s		Intergenic L1s		Case	Intragenic L1s		Intergenic L1s		Case	Intragenic L1s		Intergenic L1s			
	Observed	Expected	Observed	Expected		Observed	Expected	Observed	Expected		Observed	Expected	Observed	Expected		Observed	Expected	Observed	Expected		
Conserved	332	332	2270	2270	Conserved	969	969	5367	5367	Conserved	909	909	4031	4031	Conserved	219	219	1403	1403	MH Odds Ratio	-
other	0	0	0	0	other	0	0	0	0	other	0	0	0	0	other	0	0	0	0	MH sum chisq	-
Odds Ratio = -, 95% CI = 0.00 - inf					Odds Ratio = -, 95% CI = 0.00 - inf					Odds Ratio = -, 95% CI = 0.00 - inf					Odds Ratio = -, 95% CI = 0.00 - inf					MH p-value	-
Sum chisq = -, p-value = -					Sum chisq = -, p-value = -					Sum chisq = -, p-value = -					Sum chisq = -, p-value = -					MH 95% CI	-

N14

Case	Intragenic L1s		Intergenic L1s		Case	Intragenic L1s		Intergenic L1s		Case	Intragenic L1s		Intergenic L1s		Case	Intragenic L1s		Intergenic L1s			
	Observed	Expected	Observed	Expected		Observed	Expected	Observed	Expected		Observed	Expected	Observed	Expected		Observed	Expected	Observed	Expected		
Conserved	301	302.65	2071	2069.35	Conserved	927	930.77	5159	5155.23	Conserved	906	899.98	3985	3991.02	Conserved	217	212.52	1357	1361.48	MH Odds Ratio	1.08
other	31	29.35	199	200.65	other	42	38.23	208	211.77	other	3	9.02	46	39.98	other	2	6.48	46	41.52	MH sum chisq	0.38
Odds Ratio = 0.93, 95% CI = 0.63 - 1.39					Odds Ratio = 0.89, 95% CI = 0.63 - 1.25					Odds Ratio = 3.49, 95% CI = 1.08 - 11.23					Odds Ratio = 3.68, 95% CI = 0.89 - 15.26					MH p-value	5.35E-01
Sum chisq = 0.11712, p-value = 7.32E-01					Sum chisq = 0.45593, p-value = 5.00E-01					Sum chisq = 4.96916, p-value = 2.58E-02					Sum chisq = 3.69091, p-value = 5.47E-02					MH 95% CI	0.84-1.38
Mutated	31	29.35	199	200.65	Mutated	42	38.23	208	211.77	Mutated	3	9.02	46	39.98	Mutated	2	6.48	46	41.52	MH Odds Ratio	0.93
other	301	302.65	2071	2069.35	other	927	930.77	5159	5155.23	other	906	899.98	3985	3991.02	other	217	212.52	1357	1361.48	MH sum chisq	0.38
Odds Ratio = 1.07, 95% CI = 0.72 - 1.59					Odds Ratio = 1.12, 95% CI = 0.80 - 1.58					Odds Ratio = 0.29, 95% CI = 0.09 - 0.92					Odds Ratio = 0.27, 95% CI = 0.07 - 1.13					MH p-value	5.35E-01
Sum chisq = 0.11712, p-value = 7.32E-01					Sum chisq = 0.45593, p-value = 5.00E-01					Sum chisq = 4.96916, p-value = 2.58E-02					Sum chisq = 3.69091, p-value = 5.47E-02					MH 95% CI	0.70-1.22

E43

Case	Intragenic L1s		Intergenic L1s		Case	Intragenic L1s		Intergenic L1s		Case	Intragenic L1s		Intergenic L1s		Case	Intragenic L1s		Intergenic L1s			
	Observed	Expected	Observed	Expected		Observed	Expected	Observed	Expected		Observed	Expected	Observed	Expected		Observed	Expected	Observed	Expected		
Conserved	301	303.67	2079	2076.33	Conserved	929	931.99	5165	5162.01	Conserved	905	902.74	4001	4003.26	Conserved	216	215.22	1378	1378.78	MH Odds Ratio	0.96
other	31	28.33	191	193.67	other	40	37.01	202	204.99	other	4	6.26	30	27.74	other	3	3.78	25	24.22	MH sum chisq	0.11
Odds Ratio = 0.89, 95% CI = 0.60 - 1.33					Odds Ratio = 0.91, 95% CI = 0.64 - 1.28					Odds Ratio = 1.70, 95% CI = 0.60 - 4.83					Odds Ratio = 1.31, 95% CI = 0.39 - 4.36					MH p-value	7.37E-01
Sum chisq = 0.31636, p-value = 5.74E-01					Sum chisq = 0.29641, p-value = 5.86E-01					Sum chisq = 1.00411, p-value = 3.16E-01					Sum chisq = 0.02449, p-value = 8.76E-01					MH 95% CI	0.75-1.23
Mutated	31	28.33	191	193.67	Mutated	40	37.01	202	204.99	Mutated	4	6.26	30	27.74	Mutated	3	3.78	25	24.22	MH Odds Ratio	1.04
other	301	303.67	2079	2076.33	other	929	931.99	5165	5162.01	other	905	902.74	4001	4003.26	other	216	215.22	1378	1378.78	MH sum chisq	0.11
Odds Ratio = 1.12, 95% CI = 0.75 - 1.67					Odds Ratio = 1.10, 95% CI = 0.78 - 1.56					Odds Ratio = 0.59, 95% CI = 0.21 - 1.68					Odds Ratio = 0.77, 95% CI = 0.23 - 2.56					MH p-value	7.37E-01
Sum chisq = 0.31636, p-value = 5.74E-01					Sum chisq = 0.29641, p-value = 5.86E-01					Sum chisq = 1.00411, p-value = 3.16E-01					Sum chisq = 0.02449, p-value = 8.76E-01					MH 95% CI	0.81-1.34

Y115

Case	Intragenic L1s		Intergenic L1s		Case	Intragenic L1s		Intergenic L1s		Case	Intragenic L1s		Intergenic L1s		Case	Intragenic L1s		Intergenic L1s			
	Observed	Expected	Observed	Expected		Observed	Expected	Observed	Expected		Observed	Expected	Observed	Expected		Observed	Expected	Observed	Expected		
Conserved	306	300.87	2052	2057.13	Conserved	939	935.66	5179	5182.34	Conserved	906	902.38	3998	4001.62	Conserved	217	215.08	1376	1377.92	MH Odds Ratio	1.29
other	26	31.13	218	212.87	other	30	33.34	188	184.66	other	3	6.62	33	29.38	other	2	3.92	27	25.08	MH sum chisq	3.24
Odds Ratio = 1.25, 95% CI = 0.82 - 1.91					Odds Ratio = 1.14, 95% CI = 0.77 - 1.68					Odds Ratio = 2.49, 95% CI = 0.76 - 8.15					Odds Ratio = 2.13, 95% CI = 0.50 - 9.02					MH p-value	7.19E-02
Sum chisq = 1.07044, p-value = 3.01E-01					Sum chisq = 0.40908, p-value = 5.22E-01					Sum chisq = 2.44792, p-value = 1.18E-01					Sum chisq = 0.60239, p-value = 4.38E-01					MH 95% CI	0.98-1.69
Mutated	26	31.13	218	212.87	Mutated	30	33.34	188	184.66	Mutated	3	6.62	33	29.38	Mutated	2	3.92	27	25.08	MH Odds Ratio	0.78
other	306	300.87	2052	2057.13	other	939	935.66	5179	5182.34	other	906	902.38	3998	4001.62	other	217	215.08	1376	1377.92	MH sum chisq	3.24
Odds Ratio = 0.80, 95% CI = 0.52 - 1.22					Odds Ratio = 0.88, 95% CI = 0.59 - 1.30					Odds Ratio = 0.40, 95% CI = 0.12 - 1.31					Odds Ratio = 0.47, 95% CI = 0.11 - 1.99					MH p-value	7.19E-02
Sum chisq = 1.07044, p-value = 3.01E-01					Sum chisq = 0.40908, p-value = 5.22E-01					Sum chisq = 2.44792, p-value = 1.18E-01					Sum chisq = 0.60239, p-value = 4.38E-01					MH 95% CI	0.59-1.03

D145

Case	Intragenic L1s		Intergenic L1s		Case	Intragenic L1s		Intergenic L1s		Case	Intragenic L1s		Intergenic L1s		Case	Intragenic L1s		Intergenic L1s			
	Observed	Expected	Observed	Expected		Observed	Expected	Observed	Expected		Observed	Expected	Observed	Expected		Observed	Expected	Observed	Expected		
Conserved	297	298.19	2040	2038.81	Conserved	930	925.11	5119	5123.89	Conserved	907	898.7	3977	3985.3	Conserved	216	213.6	1366	1368.4	MH Odds Ratio	1.23
other	35	33.81	230	231.19	other	39	43.89	248	243.11	other	2	10.3	54	45.7	other	3	5.4	37	34.6	MH sum chisq	2.77
Odds Ratio = 0.96, 95% CI = 0.66 - 1.39					Odds Ratio = 1.16, 95% CI = 0.82 - 1.63					Odds Ratio = 6.16, 95% CI = 1.50 - 25.30					Odds Ratio = 1.95, 95% CI = 0.60 - 6.38					MH p-value	9.61E-02
Sum chisq = 0.05323, p-value = 8.18E-01					Sum chisq = 0.67436, p-value = 4.12E-01					Sum chisq = 8.29588, p-value = 3.97E-03					Sum chisq = 1.26495, p-value = 2.61E-01					MH 95% CI	0.96-1.57
Mutated	35	33.81	230	231.19	Mutated	39	43.89	248	243.11	Mutated	2	10.3	54	45.7	Mutated	3	5.4	37	34.6	MH Odds Ratio	0.81
other	297	298.19	2040	2038.81	other	930	925.11	5119	5123.89	other	907	898.7	3977	3985.3	other	216	213.6	1366	1368.4	MH sum chisq	2.77
Odds Ratio = 1.05, 95% CI = 0.72 - 1.52					Odds Ratio = 0.87, 95% CI = 0.61 - 1.22					Odds Ratio = 0.16, 95% CI = 0.04 - 0.67					Odds Ratio = 0.51, 95% CI = 0.16 - 1.68					MH p-value	9.61E-02
Sum chisq = 0.05323, p-value = 8.18E-01					Sum chisq = 0.67436, p-value = 4.12E-01					Sum chisq = 8.29588, p-value = 3.97E-03					Sum chisq = 1.26495, p-value = 2.61E-01					MH 95% CI	0.61-1.08

N147

Case	Intragenic L1s		Intergenic L1s		Case	Intragenic L1s		Intergenic L1s		Case	Intragenic L1s		Intergenic L1s		Case	Intragenic L1s		Intergenic L1s			
	Observed	Expected	Observed	Expected		Observed	Expected	Observed	Expected		Observed	Expected	Observed	Expected		Observed	Expected	Observed	Expected		
Conserved	307	305.97	2091	2092.03	Conserved	946	935.05	5168	5178.95	Conserved	908	901.46	3991	3997.54	Conserved	218	213.87	1366	1370.13	MH Odds Ratio	1.56
other	25	26.03	179	177.97	other	23	33.95	199	188.05	other	1	7.54	40	33.46	other	1	5.13	37	32.87	MH sum chisq	8.68
Odds Ratio = 1.05, 95% CI = 0.68 - 1.62					Odds Ratio = 1.58, 95% CI = 1.02 - 2.45					Odds Ratio = 9.10, 95% CI = 1.25 - 66.29					Odds Ratio = 5.90, 95% CI = 0.81 - 43.26					MH p-value	3.23E-03
Sum chisq = 0.05062, p-value = 8.22E-01					Sum chisq = 4.32190, p-value = 3.76E-02					Sum chisq = 7.01526, p-value = 8.08E-03					Sum chisq = 3.93695, p-value = 4.72E-02					MH 95% CI	1.15-2.11
Mutated	25	26.03	179	177.97	Mutated	23	33.95	199	188.05	Mutated	1	7.54	40	33.46	Mutated	1	5.13	37	32.87	MH Odds Ratio	0.64
other	307	305.97	2091	2092.03	other	946	935.05	5168	5178.95	other	908	901.46	3991	3997.54	other	218	213.87	1366	1370.13	MH sum chisq	8.68
Odds Ratio = 0.95, 95% CI = 0.62 - 1.47					Odds Ratio = 0.63, 95% CI = 0.41 - 0.98					Odds Ratio = 0.11, 95% CI = 0.02 - 0.80					Odds Ratio = 0.17, 95% CI = 0.02 - 1.24					MH p-value	3.23E-03
Sum chisq = 0.05062, p-value = 8.22E-01					Sum chisq = 4.32190, p-value = 3.76E-02					Sum chisq = 7.01526, p-value = 8.08E-03					Sum chisq = 3.93695, p-value = 4.72E-02					MH 95% CI	0.44-0.93

T192

Case	Intragenic L1s		Intergenic L1s		Case	Intragenic L1s		Intergenic L1s		Case	Intragenic L1s		Intergenic L1s		Case	Intragenic L1s		Intergenic L1s			
	Observed	Expected	Observed	Expected		Observed	Expected	Observed	Expected		Observed	Expected	Observed	Expected		Observed	Expected	Observed	Expected		
Conserved	286	277.9	1892	1900.1	Conserved	897	897.88	4974	4973.12	Conserved	902	896.3	3969	3974.7	Conserved	208	209.68	1345	1343.32	MH Odds Ratio	1.11
other	46	54.1	378	369.9	other	72	71.12	393	393.88	other	7	12.7	62	56.3	other	11	9.32	58	59.68	MH sum chisq	1.12
Odds Ratio = 1.24, 95% CI = 0.89 - 1.73					Odds Ratio = 0.98, 95% CI = 0.76 - 1.28					Odds Ratio = 2.01, 95% CI = 0.92 - 4.41					Odds Ratio = 0.82, 95% CI = 0.42 - 1.58					MH p-value	2.91E-01
Sum chisq = 1.66072, p-value = 1.98E-01					Sum chisq = 0.01403, p-value = 9.06E-01					Sum chisq = 3.17660, p-value = 7.47E-02					Sum chisq = 0.36743, p-value = 5.44E-01					MH 95% CI	0.92-1.34
Mutated	46	54.1	378	369.9	Mutated	72	71.12	393	393.88	Mutated	7	12.7	62	56.3	Mutated	11	9.32	58	59.68	MH Odds Ratio	0.90
other	286	277.9	1892	1900.1	other	897	897.88	4974	4973.12	other	902	896.3	3969	3974.7	other	208	209.68	1345	1343.32	MH sum chisq	1.12
Odds Ratio = 0.81, 95% CI = 0.58 - 1.12					Odds Ratio = 1.02, 95% CI = 0.78 - 1.32					Odds Ratio = 0.50, 95% CI = 0.23 - 1.09					Odds Ratio = 1.23, 95% CI = 0.63 - 2.37					MH p-value	2.91E-01
Sum chisq = 1.66072, p-value = 1.98E-01					Sum chisq = 0.01403, p-value = 9.06E-01					Sum chisq = 3.17660, p-value = 7.47E-02					Sum chisq = 0.36743, p-value = 5.44E-01					MH 95% CI	0.74-1.10

D205

Case	Intragenic L1s		Intergenic L1s		Case	Intragenic L1s		Intergenic L1s		Case	Intragenic L1s		Intergenic L1s		Case	Intragenic L1s		Intergenic L1s			
	Observed	Expected	Observed	Expected		Observed	Expected	Observed	Expected		Observed	Expected	Observed	Expected		Observed	Expected	Observed	Expected		
Conserved	291	294.74	2019	2015.26	Conserved	924	924.65	5122	5121.35	Conserved	906	901.82	3995	3999.18	Conserved	214	212.11	1357	1358.89	MH Odds Ratio	1.02
other	41	37.26	251	254.74	other	45	44.35	245	245.65	other	3	7.18	36	31.82	other	5	6.89	46	44.11	MH sum chisq	0.04
Odds Ratio = 0.88, 95% CI = 0.62 - 1.25					Odds Ratio = 0.98, 95% CI = 0.71 - 1.36					Odds Ratio = 2.72, 95% CI = 0.84 - 8.86					Odds Ratio = 1.45, 95% CI = 0.57 - 3.69					MH p-value	8.47E-01
Sum chisq = 0.48539, p-value = 4.86E-01					Sum chisq = 0.01174, p-value = 9.14E-01					Sum chisq = 3.00221, p-value = 8.32E-02					Sum chisq = 0.61654, p-value = 4.32E-01					MH 95% CI	0.81-1.28
Mutated	41	37.26	251	254.74	Mutated	45	44.35	245	245.65	Mutated	3	7.18	36	31.82	Mutated	5	6.89	46	44.11	MH Odds Ratio	0.98
other	291	294.74	2019	2015.26	other	924	924.65	5122	5121.35	other	906	901.82	3995	3999.18	other	214	212.11	1357	1358.89	MH sum chisq	0.04
Odds Ratio = 1.13, 95% CI = 0.80 - 1.61					Odds Ratio = 1.02, 95% CI = 0.74 - 1.41					Odds Ratio = 0.37, 95% CI = 0.11 - 1.20					Odds Ratio = 0.69, 95% CI = 0.27 - 1.75					MH p-value	8.47E-01
Sum chisq = 0.48539, p-value = 4.86E-01					Sum chisq = 0.01174, p-value = 9.14E-01					Sum chisq = 3.00221, p-value = 8.32E-02					Sum chisq = 0.61654, p-value = 4.32E-01					MH 95% CI	0.77-1.24

SDH288

Case	Intragenic L1s		Intergenic L1s		Case	Intragenic L1s		Intergenic L1s		Case	Intragenic L1s		Intergenic L1s		Case	Intragenic L1s		Intergenic L1s			
	Observed	Expected	Observed	Expected		Observed	Expected	Observed	Expected		Observed	Expected	Observed	Expected		Observed	Expected	Observed	Expected		
Conserved	262	248.43	1685	1698.57	Conserved	880	861.03	4750	4768.97	Conserved	895	890.78	3946	3950.22	Conserved	208	205.09	1311	1313.91	MH Odds Ratio	1.30
other	70	83.57	585	571.43	other	89	107.97	617	598.03	other	14	18.22	85	80.78	other	11	13.91	92	89.09	MH sum chisq	9.73
Odds Ratio = 1.30, 95% CI = 0.98 - 1.72					Odds Ratio = 1.28, 95% CI = 1.02 - 1.62					Odds Ratio = 1.38, 95% CI = 0.78 - 2.43					Odds Ratio = 1.33, 95% CI = 0.70 - 2.52					MH p-value	1.81E-03
Sum chisq = 3.37736, p-value = 6.61E-02					Sum chisq = 4.42923, p-value = 3.53E-02					Sum chisq = 1.22068, p-value = 2.69E-01					Sum chisq = 0.75010, p-value = 3.86E-01					MH 95% CI	1.10-1.53
Mutated	70	83.57	585	571.43	Mutated	89	107.97	617	598.03	Mutated	14	18.22	85	80.78	Mutated	11	13.91	92	89.09	MH Odds Ratio	0.77
other	262	248.43	1685	1698.57	other	880	861.03	4750	4768.97	other	895	890.78	3946	3950.22	other	208	205.09	1311	1313.91	MH sum chisq	9.73
Odds Ratio = 0.77, 95% CI = 0.58 - 1.02					Odds Ratio = 0.78, 95% CI = 0.62 - 0.98					Odds Ratio = 0.73, 95% CI = 0.41 - 1.28					Odds Ratio = 0.75, 95% CI = 0.40 - 1.43					MH p-value	1.81E-03
Sum chisq = 3.37736, p-value = 6.61E-02					Sum chisq = 4.42923, p-value = 3.53E-02					Sum chisq = 1.22068, p-value = 2.69E-01					Sum chisq = 0.75010, p-value = 3.86E-01					MH 95% CI	0.65-0.91

S_SA+1237

Case	Intragenic L1s		Intergenic L1s		Case	Intragenic L1s		Intergenic L1s		Case	Intragenic L1s		Intergenic L1s		Case	Intragenic L1s		Intergenic L1s			
	Observed	Expected	Observed	Expected		Observed	Expected	Observed	Expected		Observed	Expected	Observed	Expected		Observed	Expected	Observed	Expected		
Conserved	283	266.54	1806	1822.46	Conserved	932	922.81	5102	5111.19	Conserved	3	7.18	36	31.82	Conserved	173	139.47	860	893.53	MH Odds Ratio	1.58
other	49	65.46	464	447.54	other	37	46.19	265	255.81	other	906	901.82	3995	3999.18	other	46	79.53	543	509.47	MH sum chisq	22.78
Odds Ratio = 1.48, 95% CI = 1.08 - 2.04					Odds Ratio = 1.31, 95% CI = 0.92 - 1.86					Odds Ratio = 0.37, 95% CI = 0.11 - 1.20					Odds Ratio = 2.37, 95% CI = 1.69 - 3.34					MH p-value	1.81E-06
Sum chisq = 5.90664, p-value = 1.51E-02					Sum chisq = 2.26508, p-value = 1.32E-01					Sum chisq = 3.00221, p-value = 8.32E-02					Sum chisq = 25.65641, p-value = 4.08E-07					MH 95% CI	1.29-1.93
Mutated	49	65.46	464	447.54	Mutated	37	46.19	265	255.81	Mutated	906	901.82	3995	3999.18	Mutated	46	79.53	543	509.47	MH Odds Ratio	0.63
other	283	266.54	1806	1822.46	other	932	922.81	5102	5111.19	other	3	7.18	36	31.82	other	173	139.47	860	893.53	MH sum chisq	22.78
Odds Ratio = 0.67, 95% CI = 0.49 - 0.93					Odds Ratio = 0.76, 95% CI = 0.54 - 1.09					Odds Ratio = 2.72, 95% CI = 0.84 - 8.86					Odds Ratio = 0.42, 95% CI = 0.30 - 0.59					MH p-value	1.81E-06
Sum chisq = 5.90664, p-value = 1.51E-02					Sum chisq = 2.26508, p-value = 1.32E-01					Sum chisq = 3.00221, p-value = 8.32E-02					Sum chisq = 25.65641, p-value = 4.08E-07					MH 95% CI	0.52-0.77

SD+1881

Case	Intragenic L1s		Intergenic L1s		Case	Intragenic L1s		Intergenic L1s		Case	Intragenic L1s		Intergenic L1s		Case	Intragenic L1s		Intergenic L1s			
	Observed	Expected	Observed	Expected		Observed	Expected	Observed	Expected		Observed	Expected	Observed	Expected		Observed	Expected	Observed	Expected		
Conserved	232	240.77	1655	1646.23	Conserved	875	864.54	4778	4788.46	Conserved	899	888.58	3930	3940.42	Conserved	207	202.8	1295	1299.2	MH Odds Ratio	1.11
other	100	91.23	615	623.77	other	94	104.46	589	578.54	other	10	20.42	101	90.58	other	12	16.2	108	103.8	MH sum chisq	1.60
Odds Ratio = 0.86, 95% CI = 0.67 - 1.11					Odds Ratio = 1.15, 95% CI = 0.91 - 1.44					Odds Ratio = 2.31, 95% CI = 1.20 - 4.44					Odds Ratio = 1.44, 95% CI = 0.78 - 2.66					MH p-value	2.06E-01
Sum chisq = 1.33259, p-value = 2.48E-01					Sum chisq = 1.38465, p-value = 2.39E-01					Sum chisq = 6.67064, p-value = 9.80E-03					Sum chisq = 1.36068, p-value = 2.43E-01					MH 95% CI	0.94-1.30
Mutated	100	91.23	615	623.77	Mutated	94	104.46	589	578.54	Mutated	10	20.42	101	90.58	Mutated	12	16.2	108	103.8	MH Odds Ratio	0.90
other	232	240.77	1655	1646.23	other	875	864.54	4778	4788.46	other	899	888.58	3930	3940.42	other	207	202.8	1295	1299.2	MH sum chisq	1.60
Odds Ratio = 1.16, 95% CI = 0.90 - 1.49					Odds Ratio = 0.87, 95% CI = 0.69 - 1.10					Odds Ratio = 0.43, 95% CI = 0.23 - 0.83					Odds Ratio = 0.70, 95% CI = 0.38 - 1.28					MH p-value	2.06E-01
Sum chisq = 1.33259, p-value = 2.48E-01					Sum chisq = 1.38465, p-value = 2.39E-01					Sum chisq = 6.67064, p-value = 9.80E-03					Sum chisq = 1.36068, p-value = 2.43E-01					MH 95% CI	0.77-1.06

SA+1930

Case	Intragenic L1s		Intergenic L1s		Case	Intragenic L1s		Intergenic L1s		Case	Intragenic L1s		Intergenic L1s		Case	Intragenic L1s		Intergenic L1s			
	Observed	Expected	Observed	Expected		Observed	Expected	Observed	Expected		Observed	Expected	Observed	Expected		Observed	Expected	Observed	Expected		
Conserved	2	2.68	19	18.32	Conserved	2	2.45	14	13.55											MH Odds Ratio	0.75
other	330	329.32	2251	2251.68	other	967	966.55	5353	5353.45											MH sum chisq	0.29
Odds Ratio = 0.72, 95% CI = 0.17 - 3.10					Odds Ratio = 0.79, 95% CI = 0.18 - 3.49															MH p-value	5.90E-01
Sum chisq = 0.01389, p-value = 9.06E-01					Sum chisq = 0.00136, p-value = 9.71E-01															MH 95% CI	0.27-2.13
Mutated	330	329.32	2251	2251.68	Mutated	967	966.55	5353	5353.45	Mutated	909	909	4031	4031	Mutated	219	219	1403	1403	MH Odds Ratio	-
other	2	2.68	19	18.32	other	2	2.45	14	13.55	other	0	0	0	0	other	0	0	0	0	MH sum chisq	-
Odds Ratio = 1.39, 95% CI = 0.32 - 6.01					Odds Ratio = 1.26, 95% CI = 0.29 - 5.57					Odds Ratio = -, 95% CI = 0.00 - inf					Odds Ratio = -, 95% CI = 0.00 - inf					MH p-value	-
Sum chisq = 0.01389, p-value = 9.06E-01					Sum chisq = 0.00136, p-value = 9.71E-01					Sum chisq = -, p-value = -					Sum chisq = -, p-value = -					MH 95% CI	-

SD+2036

Case	Intragenic L1s		Intergenic L1s		Case	Intragenic L1s		Intergenic L1s		Case	Intragenic L1s		Intergenic L1s		Case	Intragenic L1s		Intergenic L1s			
	Observed	Expected	Observed	Expected		Observed	Expected	Observed	Expected		Observed	Expected	Observed	Expected		Observed	Expected	Observed	Expected		
Conserved	7	6.76	46	46.24	Conserved	7	10.09	59	55.91	Conserved	2	1.29	5	5.71	Conserved	1	0.68	4	4.32	MH Odds Ratio	0.89
other	325	325.24	2224	2223.76	other	962	958.91	5308	5311.09	other	907	907.71	4026	4025.29	other	218	218.32	1399	1398.68	MH sum chisq	0.21
Odds Ratio = 1.04, 95% CI = 0.47 - 2.33					Odds Ratio = 0.65, 95% CI = 0.30 - 1.44					Odds Ratio = 1.78, 95% CI = 0.34 - 9.17					Odds Ratio = 1.60, 95% CI = 0.18 - 14.42					MH p-value	6.48E-01
Sum chisq = 0.00976, p-value = 9.21E-01					Sum chisq = 1.13123, p-value = 2.88E-01					Sum chisq = 0.04280, p-value = 8.36E-01					Sum chisq = 0.05266, p-value = 8.18E-01					MH 95% CI	0.52-1.51
Mutated	325	325.24	2224	2223.76	Mutated	962	958.91	5308	5311.09	Mutated	907	907.71	4026	4025.29	Mutated	218	218.32	1399	1398.68	MH Odds Ratio	1.13
other	7	6.76	46	46.24	other	7	10.09	59	55.91	other	2	1.29	5	5.71	other	1	0.68	4	4.32	MH sum chisq	0.21
Odds Ratio = 0.96, 95% CI = 0.43 - 2.15					Odds Ratio = 1.53, 95% CI = 0.70 - 3.35					Odds Ratio = 0.56, 95% CI = 0.11 - 2.91					Odds Ratio = 0.62, 95% CI = 0.07 - 5.60					MH p-value	6.48E-01
Sum chisq = 0.00976, p-value = 9.21E-01					Sum chisq = 1.13123, p-value = 2.88E-01					Sum chisq = 0.04280, p-value = 8.36E-01					Sum chisq = 0.05266, p-value = 8.18E-01					MH 95% CI	0.67-1.89

R363

Case	Intragenic L1s		Intergenic L1s		Case	Intragenic L1s		Intergenic L1s		Case	Intragenic L1s		Intergenic L1s		Case	Intragenic L1s		Intergenic L1s			
	Observed	Expected	Observed	Expected		Observed	Expected	Observed	Expected		Observed	Expected	Observed	Expected		Observed	Expected	Observed	Expected		
Conserved	293	294.49	2015	2013.51	Conserved	928	922.97	5107	5112.03	Conserved	897	896.86	3977	3977.14	Conserved	214	211.98	1356	1358.02	MH Odds Ratio	1.07
other	39	37.51	255	256.49	other	41	46.03	260	254.97	other	12	12.14	54	53.86	other	5	7.02	47	44.98	MH sum chisq	0.40
Odds Ratio = 0.95, 95% CI = 0.66 - 1.36					Odds Ratio = 1.15, 95% CI = 0.82 - 1.61					Odds Ratio = 1.01, 95% CI = 0.54 - 1.91					Odds Ratio = 1.48, 95% CI = 0.58 - 3.77					MH p-value	5.29E-01
Sum chisq = 0.07620, p-value = 7.83E-01					Sum chisq = 0.68219, p-value = 4.09E-01					Sum chisq = 0.00214, p-value = 9.63E-01					Sum chisq = 0.69481, p-value = 4.05E-01					MH 95% CI	0.86-1.34
Mutated	39	37.51	255	256.49	Mutated	41	46.03	260	254.97	Mutated	12	12.14	54	53.86	Mutated	5	7.02	47	44.98	MH Odds Ratio	0.93
other	293	294.49	2015	2013.51	other	928	922.97	5107	5112.03	other	897	896.86	3977	3977.14	other	214	211.98	1356	1358.02	MH sum chisq	0.40
Odds Ratio = 1.05, 95% CI = 0.73 - 1.51					Odds Ratio = 0.87, 95% CI = 0.62 - 1.22					Odds Ratio = 0.99, 95% CI = 0.52 - 1.85					Odds Ratio = 0.67, 95% CI = 0.27 - 1.71					MH p-value	5.29E-01
Sum chisq = 0.07620, p-value = 7.83E-01					Sum chisq = 0.68219, p-value = 4.09E-01					Sum chisq = 0.00214, p-value = 9.63E-01					Sum chisq = 0.69481, p-value = 4.05E-01					MH 95% CI	0.74-1.16

FADD700

Case	Intragenic L1s		Intergenic L1s		Case	Intragenic L1s		Intergenic L1s		Case	Intragenic L1s		Intergenic L1s		Case	Intragenic L1s		Intergenic L1s			
	Observed	Expected	Observed	Expected		Observed	Expected	Observed	Expected		Observed	Expected	Observed	Expected		Observed	Expected	Observed	Expected		
Conserved	232	232.35	1589	1588.65	Conserved	868	851.7	4701	4717.3	Conserved	902	885.45	3910	3926.55	Conserved	211	198.48	1259	1271.52	MH Odds Ratio	1.30
other	100	99.65	681	681.35	other	101	117.3	666	649.7	other	7	23.55	121	104.45	other	8	20.52	144	131.48	MH sum chisq	11.07
Odds Ratio = 0.99, 95% CI = 0.77 - 1.28					Odds Ratio = 1.22, 95% CI = 0.98 - 1.52					Odds Ratio = 3.99, 95% CI = 1.85 - 8.57					Odds Ratio = 3.02, 95% CI = 1.46 - 6.24					MH p-value	8.76E-04
Sum chisq = 0.00200, p-value = 9.64E-01					Sum chisq = 3.04284, p-value = 8.11E-02					Sum chisq = 14.63605, p-value = 1.30E-04					Sum chisq = 9.74751, p-value = 1.80E-03					MH 95% CI	1.11-1.53
Mutated	100	99.65	681	681.35	Mutated	101	117.3	666	649.7	Mutated	7	23.55	121	104.45	Mutated	8	20.52	144	131.48	MH Odds Ratio	0.77
other	232	232.35	1589	1588.65	other	868	851.7	4701	4717.3	other	902	885.45	3910	3926.55	other	211	198.48	1259	1271.52	MH sum chisq	11.07
Odds Ratio = 1.01, 95% CI = 0.78 - 1.29					Odds Ratio = 0.82, 95% CI = 0.66 - 1.02					Odds Ratio = 0.25, 95% CI = 0.12 - 0.54					Odds Ratio = 0.33, 95% CI = 0.16 - 0.69					MH p-value	8.76E-04
Sum chisq = 0.00200, p-value = 9.64E-01					Sum chisq = 3.04284, p-value = 8.11E-02					Sum chisq = 14.63605, p-value = 1.30E-04					Sum chisq = 9.74751, p-value = 1.80E-03					MH 95% CI	0.64-0.91

HMKK1091

Case	Intragenic L1s		Intergenic L1s		Case	Intragenic L1s		Intergenic L1s		Case	Intragenic L1s		Intergenic L1s		Case	Intragenic L1s		Intergenic L1s			
	Observed	Expected	Observed	Expected		Observed	Expected	Observed	Expected		Observed	Expected	Observed	Expected		Observed	Expected	Observed	Expected		
Conserved	274	254.42	1720	1739.58	Conserved	874	870.05	4815	4818.95	Conserved	894	880.85	3893	3906.15	Conserved	212	205.36	1309	1315.64	MH Odds Ratio	1.34
other	58	77.58	550	530.42	other	95	98.95	552	548.05	other	15	28.15	138	124.85	other	7	13.64	94	87.36	MH sum chisq	11.69
Odds Ratio = 1.51, 95% CI = 1.12 - 2.04					Odds Ratio = 1.05, 95% CI = 0.84 - 1.33					Odds Ratio = 2.11, 95% CI = 1.23 - 3.62					Odds Ratio = 2.17, 95% CI = 1.00 - 4.75					MH p-value	6.27E-04
Sum chisq = 7.38980, p-value = 6.56E-03					Sum chisq = 0.20725, p-value = 6.49E-01					Sum chisq = 7.77168, p-value = 5.31E-03					Sum chisq = 3.98223, p-value = 4.60E-02					MH 95% CI	1.13-1.58
Mutated	58	77.58	550	530.42	Mutated	95	98.95	552	548.05	Mutated	15	28.15	138	124.85	Mutated	7	13.64	94	87.36	MH Odds Ratio	0.75
other	274	254.42	1720	1739.58	other	874	870.05	4815	4818.95	other	894	880.85	3893	3906.15	other	212	205.36	1309	1315.64	MH sum chisq	11.69
Odds Ratio = 0.66, 95% CI = 0.49 - 0.89					Odds Ratio = 0.95, 95% CI = 0.75 - 1.19					Odds Ratio = 0.47, 95% CI = 0.28 - 0.81					Odds Ratio = 0.46, 95% CI = 0.21 - 1.00					MH p-value	6.27E-04
Sum chisq = 7.38980, p-value = 6.56E-03					Sum chisq = 0.20725, p-value = 6.49E-01					Sum chisq = 7.77168, p-value = 5.31E-03					Sum chisq = 3.98223, p-value = 4.60E-02					MH 95% CI	0.63-0.89

SA+4117

Case	Intragenic L1s		Intergenic L1s		Case	Intragenic L1s		Intergenic L1s		Case	Intragenic L1s		Intergenic L1s		Case	Intragenic L1s		Intergenic L1s			
	Observed	Expected	Observed	Expected		Observed	Expected	Observed	Expected		Observed	Expected	Observed	Expected		Observed	Expected	Observed	Expected		
Conserved	310	308.65	2109	2110.35	Conserved	945	938.41	5191	5197.59	Conserved	903	901.46	3996	3997.54	Conserved	216	214.81	1375	1376.19	MH Odds Ratio	1.24
other	22	23.35	161	159.65	other	24	30.59	176	169.41	other	6	7.54	35	33.46	other	3	4.19	28	26.81	MH sum chisq	2.12
Odds Ratio = 1.08, 95% CI = 0.68 - 1.71					Odds Ratio = 1.34, 95% CI = 0.87 - 2.06					Odds Ratio = 1.32, 95% CI = 0.55 - 3.14					Odds Ratio = 1.47, 95% CI = 0.44 - 4.86					MH p-value	1.45E-01
Sum chisq = 0.09620, p-value = 7.56E-01					Sum chisq = 1.72928, p-value = 1.89E-01					Sum chisq = 0.39066, p-value = 5.32E-01					Sum chisq = 0.13235, p-value = 7.16E-01					MH 95% CI	0.93-1.65
Mutated	22	23.35	161	159.65	Mutated	24	30.59	176	169.41	Mutated	6	7.54	35	33.46	Mutated	3	4.19	28	26.81	MH Odds Ratio	0.81
other	310	308.65	2109	2110.35	other	945	938.41	5191	5197.59	other	903	901.46	3996	3997.54	other	216	214.81	1375	1376.19	MH sum chisq	2.12
Odds Ratio = 0.93, 95% CI = 0.59 - 1.47					Odds Ratio = 0.75, 95% CI = 0.49 - 1.15					Odds Ratio = 0.76, 95% CI = 0.32 - 1.81					Odds Ratio = 0.68, 95% CI = 0.21 - 2.26					MH p-value	1.45E-01
Sum chisq = 0.09620, p-value = 7.56E-01					Sum chisq = 1.72928, p-value = 1.89E-01					Sum chisq = 0.39066, p-value = 5.32E-01					Sum chisq = 0.13235, p-value = 7.16E-01					MH 95% CI	0.61-1.08

STS1096

Case	Intragenic L1s		Intergenic L1s		Case	Intragenic L1s		Intergenic L1s		Case	Intragenic L1s		Intergenic L1s		Case	Intragenic L1s		Intergenic L1s			
	Observed	Expected	Observed	Expected		Observed	Expected	Observed	Expected		Observed	Expected	Observed	Expected		Observed	Expected	Observed	Expected		
Conserved	170	178.76	1231	1222.24	Conserved	792	768.81	4235	4258.19	Conserved	900	887.66	3924	3936.34	Conserved	207	201.45	1285	1290.55	MH Odds Ratio	1.15
other	162	153.24	1039	1047.76	other	177	200.19	1132	1108.81	other	9	21.34	107	94.66	other	12	17.55	118	112.45	MH sum chisq	4.40
Odds Ratio = 0.89, 95% CI = 0.70 - 1.12					Odds Ratio = 1.20, 95% CI = 1.00 - 1.43					Odds Ratio = 2.73, 95% CI = 1.38 - 5.40					Odds Ratio = 1.58, 95% CI = 0.86 - 2.92					MH p-value	3.60E-02
Sum chisq = 1.06593, p-value = 3.02E-01					Sum chisq = 3.99802, p-value = 4.56E-02					Sum chisq = 8.96018, p-value = 2.76E-03					Sum chisq = 2.20750, p-value = 1.37E-01					MH 95% CI	1.01-1.32
Mutated	162	153.24	1039	1047.76	Mutated	177	200.19	1132	1108.81	Mutated	9	21.34	107	94.66	Mutated	12	17.55	118	112.45	MH Odds Ratio	0.87
other	170	178.76	1231	1222.24	other	792	768.81	4235	4258.19	other	900	887.66	3924	3936.34	other	207	201.45	1285	1290.55	MH sum chisq	4.40
Odds Ratio = 1.13, 95% CI = 0.90 - 1.42					Odds Ratio = 0.84, 95% CI = 0.70 - 1.00					Odds Ratio = 0.37, 95% CI = 0.19 - 0.73					Odds Ratio = 0.63, 95% CI = 0.34 - 1.16					MH p-value	3.60E-02
Sum chisq = 1.06593, p-value = 3.02E-01					Sum chisq = 3.99802, p-value = 4.56E-02					Sum chisq = 8.96018, p-value = 2.76E-03					Sum chisq = 2.20750, p-value = 1.37E-01					MH 95% CI	0.76-1.00

I1220

Case	Intragenic L1s		Intergenic L1s		Case	Intragenic L1s		Intergenic L1s		Case	Intragenic L1s		Intergenic L1s		Case	Intragenic L1s		Intergenic L1s			
	Observed	Expected	Observed	Expected		Observed	Expected	Observed	Expected		Observed	Expected	Observed	Expected		Observed	Expected	Observed	Expected		
Conserved	311	304.44	2075	2081.56	Conserved	936	924.65	5110	5121.35	Conserved	902	894.65	3960	3967.35	Conserved	213	212.65	1362	1362.35	MH Odds Ratio	1.47
other	21	27.56	195	188.44	other	33	44.35	257	245.65	other	7	14.35	71	63.65	other	6	6.35	41	40.65	MH sum chisq	8.77
Odds Ratio = 1.39, 95% CI = 0.87 - 2.22					Odds Ratio = 1.43, 95% CI = 0.99 - 2.06					Odds Ratio = 2.31, 95% CI = 1.06 - 5.04					Odds Ratio = 1.07, 95% CI = 0.45 - 2.55					MH p-value	3.06E-03
Sum chisq = 1.95203, p-value = 1.62E-01					Sum chisq = 3.59432, p-value = 5.80E-02					Sum chisq = 4.69008, p-value = 3.03E-02					Sum chisq = 0.02244, p-value = 8.81E-01					MH 95% CI	1.14-1.91
Mutated	21	27.56	195	188.44	Mutated	33	44.35	257	245.65	Mutated	7	14.35	71	63.65	Mutated	6	6.35	41	40.65	MH Odds Ratio	0.68
other	311	304.44	2075	2081.56	other	936	924.65	5110	5121.35	other	902	894.65	3960	3967.35	other	213	212.65	1362	1362.35	MH sum chisq	8.77
Odds Ratio = 0.72, 95% CI = 0.45 - 1.14					Odds Ratio = 0.70, 95% CI = 0.48 - 1.01					Odds Ratio = 0.43, 95% CI = 0.20 - 0.94					Odds Ratio = 0.94, 95% CI = 0.39 - 2.23					MH p-value	3.06E-03
Sum chisq = 1.95203, p-value = 1.62E-01					Sum chisq = 3.59432, p-value = 5.80E-02					Sum chisq = 4.69008, p-value = 3.03E-02					Sum chisq = 0.02244, p-value = 8.81E-01					MH 95% CI	0.52-0.88

T1259

Case	Intragenic L1s		Intergenic L1s		Case	Intragenic L1s		Intergenic L1s		Case	Intragenic L1s		Intergenic L1s		Case	Intragenic L1s		Intergenic L1s			
	Observed	Expected	Observed	Expected		Observed	Expected	Observed	Expected		Observed	Expected	Observed	Expected		Observed	Expected	Observed	Expected		
Conserved	299	290.15	1975	1983.85	Conserved	914	915.32	5071	5069.68	Conserved	898	891.89	3949	3955.11	Conserved	208	207.79	1331	1331.21	MH Odds Ratio	1.16
other	33	41.85	295	286.15	other	55	53.68	296	297.32	other	11	17.11	82	75.89	other	11	11.21	72	71.79	MH sum chisq	1.96
Odds Ratio = 1.35, 95% CI = 0.93 - 1.98					Odds Ratio = 0.97, 95% CI = 0.72 - 1.30					Odds Ratio = 1.70, 95% CI = 0.90 - 3.19					Odds Ratio = 1.02, 95% CI = 0.53 - 1.96					MH p-value	1.61E-01
Sum chisq = 2.45509, p-value = 1.17E-01					Sum chisq = 0.04054, p-value = 8.40E-01					Sum chisq = 2.72723, p-value = 9.87E-02					Sum chisq = 0.00464, p-value = 9.46E-01					MH 95% CI	0.94-1.43
Mutated	33	41.85	295	286.15	Mutated	55	53.68	296	297.32	Mutated	11	17.11	82	75.89	Mutated	11	11.21	72	71.79	MH Odds Ratio	0.86
other	299	290.15	1975	1983.85	other	914	915.32	5071	5069.68	other	898	891.89	3949	3955.11	other	208	207.79	1331	1331.21	MH sum chisq	1.96
Odds Ratio = 0.74, 95% CI = 0.51 - 1.08					Odds Ratio = 1.03, 95% CI = 0.77 - 1.39					Odds Ratio = 0.59, 95% CI = 0.31 - 1.11					Odds Ratio = 0.98, 95% CI = 0.51 - 1.87					MH p-value	1.61E-01
Sum chisq = 2.45509, p-value = 1.17E-01					Sum chisq = 0.04054, p-value = 8.40E-01					Sum chisq = 2.72723, p-value = 9.87E-02					Sum chisq = 0.00464, p-value = 9.46E-01					MH 95% CI	0.70-1.06

BG_SA+4578

Case	Intragenic L1s		Intergenic L1s		Case	Intragenic L1s		Intergenic L1s		Case	Intragenic L1s		Intergenic L1s		Case	Intragenic L1s		Intergenic L1s			
	Observed	Expected	Observed	Expected		Observed	Expected	Observed	Expected		Observed	Expected	Observed	Expected		Observed	Expected	Observed	Expected		
Conserved	34	37	256	253	Conserved	690	672.92	3710	3727.08	Conserved	885	852.69	3749	3781.31	Conserved	172	140.55	869	900.45	MH Odds Ratio	1.33
other	298	295	2014	2017	other	279	296.08	1657	1639.92	other	24	56.31	282	249.69	other	47	78.45	534	502.55	MH sum chisq	20.92
Odds Ratio = 0.90, 95% CI = 0.62 - 1.31					Odds Ratio = 1.10, 95% CI = 0.95 - 1.28					Odds Ratio = 2.77, 95% CI = 1.82 - 4.23					Odds Ratio = 2.25, 95% CI = 1.60 - 3.16					MH p-value	4.78E-06
Sum chisq = 0.31425, p-value = 5.75E-01					Sum chisq = 1.67563, p-value = 1.96E-01					Sum chisq = 24.21620, p-value = 8.61E-07					Sum chisq = 22.70637, p-value = 1.89E-06					MH 95% CI	1.17-1.51
Mutated	298	295	2014	2017	Mutated	279	296.08	1657	1639.92	Mutated	24	56.31	282	249.69	Mutated	47	78.45	534	502.55	MH Odds Ratio	0.75
other	34	37	256	253	other	690	672.92	3710	3727.08	other	885	852.69	3749	3781.31	other	172	140.55	869	900.45	MH sum chisq	20.92
Odds Ratio = 1.11, 95% CI = 0.76 - 1.63					Odds Ratio = 0.91, 95% CI = 0.78 - 1.05					Odds Ratio = 0.36, 95% CI = 0.24 - 0.55					Odds Ratio = 0.44, 95% CI = 0.32 - 0.62					MH p-value	4.78E-06
Sum chisq = 0.31425, p-value = 5.75E-01					Sum chisq = 1.67563, p-value = 1.96E-01					Sum chisq = 24.21620, p-value = 8.61E-07					Sum chisq = 22.70637, p-value = 1.89E-06					MH 95% CI	0.66-0.86

BG_SD+4694

Case	Intragenic L1s		Intergenic L1s		Case	Intragenic L1s		Intergenic L1s		Case	Intragenic L1s		Intergenic L1s		Case	Intragenic L1s		Intergenic L1s			
	Observed	Expected	Observed	Expected		Observed	Expected	Observed	Expected		Observed	Expected	Observed	Expected		Observed	Expected	Observed	Expected		
Conserved	287	286.32	1957	1957.68	Conserved	921	906.6	5007	5021.4	Conserved	898	882.87	3900	3915.13	Conserved	213	207.39	1323	1328.61	MH Odds Ratio	1.42
other	45	45.68	313	312.32	other	48	62.4	360	345.6	other	11	26.13	131	115.87	other	6	11.61	80	74.39	MH sum chisq	11.25
Odds Ratio = 1.02, 95% CI = 0.73 - 1.43					Odds Ratio = 1.38, 95% CI = 1.01 - 1.88					Odds Ratio = 2.74, 95% CI = 1.48 - 5.10					Odds Ratio = 2.15, 95% CI = 0.92 - 4.98					MH p-value	7.97E-04
Sum chisq = 0.01340, p-value = 9.08E-01					Sum chisq = 4.19189, p-value = 4.06E-02					Sum chisq = 11.05311, p-value = 8.85E-04					Sum chisq = 3.31080, p-value = 6.88E-02					MH 95% CI	1.15-1.75

Mutated	45	45.68	313	312.32	Mutated	48	62.4	360	345.6	Mutated	11	26.13	131	115.87	Mutated	6	11.61	80	74.39	MH Odds Ratio	0.70
other	287	286.32	1957	1957.68	other	921	906.6	5007	5021.4	other	898	882.87	3900	3915.13	other	213	207.39	1323	1328.61	MH sum chisq	11.25
Odds Ratio = 0.98, 95% CI = 0.70 - 1.37					Odds Ratio = 0.72, 95% CI = 0.53 - 0.99					Odds Ratio = 0.36, 95% CI = 0.20 - 0.68					Odds Ratio = 0.47, 95% CI = 0.20 - 1.08					MH p-value	7.97E-04
Sum chisq = 0.01340, p-value = 9.08E-01					Sum chisq = 4.19189, p-value = 4.06E-02					Sum chisq = 11.05311, p-value = 8.85E-04					Sum chisq = 3.31080, p-value = 6.88E-02					MH 95% CI	0.57-0.88
**BG_SD+4903**																					
Case	Intragenic L1s		Intergenic L1s		Case	Intragenic L1s		Intergenic L1s		Case	Intragenic L1s		Intergenic L1s		Case	Intragenic L1s		Intergenic L1s			
	Observed	Expected	Observed	Expected		Observed	Expected	Observed	Expected		Observed	Expected	Observed	Expected		Observed	Expected	Observed	Expected		
Conserved	304	286.19	1939	1956.81	Conserved	916	902.47	4985	4998.53	Conserved	897	890.6	3943	3949.4	Conserved	213	210.36	1345	1347.64	MH Odds Ratio	1.52
other	28	45.81	331	313.19	other	53	66.53	382	368.47	other	12	18.4	88	81.6	other	6	8.64	58	55.36	MH sum chisq	14.98
Odds Ratio = 1.85, 95% CI = 1.24 - 2.78					Odds Ratio = 1.32, 95% CI = 0.99 - 1.78					Odds Ratio = 1.67, 95% CI = 0.91 - 3.06					Odds Ratio = 1.53, 95% CI = 0.65 - 3.59					MH p-value	1.09E-04
Sum chisq = 9.20411, p-value = 2.41E-03					Sum chisq = 3.48640, p-value = 6.19E-02					Sum chisq = 2.78502, p-value = 9.51E-02					Sum chisq = 0.97163, p-value = 3.24E-01					MH 95% CI	1.23-1.89
Mutated	28	45.81	331	313.19	Mutated	53	66.53	382	368.47	Mutated	12	18.4	88	81.6	Mutated	6	8.64	58	55.36	MH Odds Ratio	0.66
other	304	286.19	1939	1956.81	other	916	902.47	4985	4998.53	other	897	890.6	3943	3949.4	other	213	210.36	1345	1347.64	MH sum chisq	14.98
Odds Ratio = 0.54, 95% CI = 0.36 - 0.81					Odds Ratio = 0.76, 95% CI = 0.56 - 1.01					Odds Ratio = 0.60, 95% CI = 0.33 - 1.10					Odds Ratio = 0.65, 95% CI = 0.28 - 1.53					MH p-value	1.09E-04
Sum chisq = 9.20411, p-value = 2.41E-03					Sum chisq = 3.48640, p-value = 6.19E-02					Sum chisq = 2.78502, p-value = 9.51E-02					Sum chisq = 0.97163, p-value = 3.24E-01					MH 95% CI	0.53-0.82
**3' UTR**																					
**SD+5094**																					
Case	Intragenic L1s		Intergenic L1s		Case	Intragenic L1s		Intergenic L1s		Case	Intragenic L1s		Intergenic L1s		Case	Intragenic L1s		Intergenic L1s			
	Observed	Expected	Observed	Expected		Observed	Expected	Observed	Expected		Observed	Expected	Observed	Expected		Observed	Expected	Observed	Expected		
Conserved	3	3.83	27	26.17	Conserved	8	4.89	24	27.11	Conserved	1	1.47	7	6.53						MH Odds Ratio	1.22
other	329	328.17	2243	2243.83	other	961	964.11	5343	5339.89	other	908	907.53	4024	4024.47						MH sum chisq	0.38
Odds Ratio = 0.76, 95% CI = 0.23 - 2.51					Odds Ratio = 1.85, 95% CI = 0.83 - 4.14					Odds Ratio = 0.63, 95% CI = 0.08 - 5.15										MH p-value	5.38E-01
Sum chisq = 0.03256, p-value = 8.57E-01					Sum chisq = 1.64662, p-value = 1.99E-01					Sum chisq = 0.00065, p-value = 9.80E-01										MH 95% CI	0.61-2.41
Mutated	329	328.04	2242	2242.96	Mutated	961	964.11	5343	5339.89	Mutated	908	907.53	4024	4024.47	Mutated	219	219	1403	1403	MH Odds Ratio	-
other	3	3.96	28	27.04	other	8	4.89	24	27.11	other	1	1.47	7	6.53	other	0	0	0	0	MH sum chisq	-
Odds Ratio = 1.37, 95% CI = 0.41 - 4.53					Odds Ratio = 0.54, 95% CI = 0.24 - 1.20					Odds Ratio = 1.58, 95% CI = 0.19 - 12.85					Odds Ratio = -, 95% CI = 0.00 - inf					MH p-value	-
Sum chisq = 0.06083, p-value = 8.05E-01					Sum chisq = 1.64662, p-value = 1.99E-01					Sum chisq = 0.00065, p-value = 9.80E-01					Sum chisq = -, p-value = -					MH 95% CI	-
**SA+5260**																					
Case	Intragenic L1s		Intergenic L1s		Case	Intragenic L1s		Intergenic L1s		Case	Intragenic L1s		Intergenic L1s		Case	Intragenic L1s		Intergenic L1s			
	Observed	Expected	Observed	Expected		Observed	Expected	Observed	Expected		Observed	Expected	Observed	Expected		Observed	Expected	Observed	Expected		
Conserved	309	297.8	2025	2036.2	Conserved	608	651.96	3655	3611.04	Conserved	903	890.23	3935	3947.77	Conserved	207	209.68	1346	1343.32	MH Odds Ratio	0.91
other	23	34.2	245	233.8	other	361	317.04	1712	1755.96	other	6	18.77	96	83.23	other	12	9.32	57	59.68	MH sum chisq	2.23
Odds Ratio = 1.63, 95% CI = 1.04 - 2.53					Odds Ratio = 0.79, 95% CI = 0.68 - 0.91					Odds Ratio = 3.67, 95% CI = 1.60 - 8.40					Odds Ratio = 0.73, 95% CI = 0.39 - 1.38					MH p-value	1.35E-01
Sum chisq = 4.68370, p-value = 3.05E-02					Sum chisq = 10.69743, p-value = 1.07E-03					Sum chisq = 10.87026, p-value = 9.77E-04					Sum chisq = 0.93348, p-value = 3.34E-01					MH 95% CI	0.79-1.03
Mutated	23	34.07	244	232.93	Mutated	361	317.04	1712	1755.96	Mutated	6	18.77	96	83.23	Mutated	12	9.32	57	59.68	MH Odds Ratio	1.10
other	309	297.93	2026	2037.07	other	608	651.96	3655	3611.04	other	903	890.23	3935	3947.77	other	207	209.68	1346	1343.32	MH sum chisq	2.26
Odds Ratio = 0.62, 95% CI = 0.40 - 0.96					Odds Ratio = 1.27, 95% CI = 1.10 - 1.46					Odds Ratio = 0.27, 95% CI = 0.12 - 0.62					Odds Ratio = 1.37, 95% CI = 0.72 - 2.59					MH p-value	1.33E-01
Sum chisq = 4.59272, p-value = 3.21E-02					Sum chisq = 10.69743, p-value = 1.07E-03					Sum chisq = 10.87026, p-value = 9.77E-04					Sum chisq = 0.93348, p-value = 3.34E-01					MH 95% CI	0.96-1.27
**SA+5614**																					
Case	Intragenic L1s		Intergenic L1s		Case	Intragenic L1s		Intergenic L1s		Case	Intragenic L1s		Intergenic L1s		Case	Intragenic L1s		Intergenic L1s			
	Observed	Expected	Observed	Expected		Observed	Expected	Observed	Expected		Observed	Expected	Observed	Expected		Observed	Expected	Observed	Expected		
Conserved	241	236.43	1612	1616.57	Conserved	844	797.1	4368	4414.9	Conserved	883	882.69	3914	3914.31	Conserved	212	207.39	1324	1328.61	MH Odds Ratio	1.33
other	91	95.57	658	653.43	other	125	171.9	999	952.1	other	26	26.31	117	116.69	other	7	11.61	79	74.39	MH sum chisq	15.17
Odds Ratio = 1.08, 95% CI = 0.84 - 1.40					Odds Ratio = 1.54, 95% CI = 1.26 - 1.89					Odds Ratio = 1.02, 95% CI = 0.66 - 1.56					Odds Ratio = 1.81, 95% CI = 0.82 - 3.97					MH p-value	9.81E-05
Sum chisq = 0.35145, p-value = 5.53E-01					Sum chisq = 18.36361, p-value = 1.83E-05					Sum chisq = 0.00470, p-value = 9.45E-01					Sum chisq = 2.23595, p-value = 1.35E-01					MH 95% CI	1.15-1.54
Mutated	91	95.44	657	652.56	Mutated	125	171.9	999	952.1	Mutated	26	26.31	117	116.69	Mutated	7	11.61	79	74.39	MH Odds Ratio	0.75
other	241	236.56	1613	1617.44	other	844	797.1	4368	4414.9	other	883	882.69	3914	3914.31	other	212	207.39	1324	1328.61	MH sum chisq	15.11
Odds Ratio = 0.93, 95% CI = 0.72 - 1.20					Odds Ratio = 0.65, 95% CI = 0.53 - 0.79					Odds Ratio = 0.99, 95% CI = 0.64 - 1.52					Odds Ratio = 0.55, 95% CI = 0.25 - 1.21					MH p-value	1.02E-04
Sum chisq = 0.33235, p-value = 5.64E-01					Sum chisq = 18.36361, p-value = 1.83E-05					Sum chisq = 0.00470, p-value = 9.45E-01					Sum chisq = 2.23595, p-value = 1.35E-01					MH 95% CI	0.65-0.87
**PolyA Signal**																					
Case	Intragenic L1s		Intergenic L1s		Case	Intragenic L1s		Intergenic L1s		Case	Intragenic L1s		Intergenic L1s		Case	Intragenic L1s		Intergenic L1s			
	Observed	Expected	Observed	Expected		Observed	Expected	Observed	Expected		Observed	Expected	Observed	Expected		Observed	Expected	Observed	Expected		
Conserved	234	231.46	1580	1582.54	Conserved	857	846.5	4678	4688.5	Conserved	885	870.73	3847	3861.27	Conserved	204	192.27	1220	1231.73	MH Odds Ratio	1.23
other	98	100.54	690	687.46	other	112	122.5	689	678.5	other	24	38.27	184	169.73	other	15	26.73	183	171.27	MH sum chisq	7.54
Odds Ratio = 1.04, 95% CI = 0.81 - 1.34					Odds Ratio = 1.13, 95% CI = 0.91 - 1.39					Odds Ratio = 1.76, 95% CI = 1.15 - 2.72					Odds Ratio = 2.04, 95% CI = 1.18 - 3.52					MH p-value	6.03E-03
Sum chisq = 0.10585, p-value = 7.45E-01					Sum chisq = 1.21657, p-value = 2.70E-01					Sum chisq = 6.81033, p-value = 9.06E-03					Sum chisq = 6.78177, p-value = 9.21E-03					MH 95% CI	1.06-1.42
Mutated	98	100.42	689	686.58	Mutated	112	122.5	689	678.5	Mutated	24	38.27	184	169.73	Mutated	15	26.73	183	171.27	MH Odds Ratio	0.82
other	234	231.58	1581	1583.42	other	857	846.5	4678	4688.5	other	885	870.73	3847	3861.27	other	204	192.27	1220	1231.73	MH sum chisq	7.50
Odds Ratio = 0.96, 95% CI = 0.75 - 1.24					Odds Ratio = 0.89, 95% CI = 0.72 - 1.10					Odds Ratio = 0.57, 95% CI = 0.37 - 0.87					Odds Ratio = 0.49, 95% CI = 0.28 - 0.85					MH p-value	6.18E-03
Sum chisq = 0.09557, p-value = 7.57E-01					Sum chisq = 1.21657, p-value = 2.70E-01					Sum chisq = 6.81033, p-value = 9.06E-03					Sum chisq = 6.78177, p-value = 9.21E-03					MH 95% CI	0.70-0.95

Table 1.2 Student's *t-tests* of non-categorical mouse L1 characteristics.

Student's t-test of non-categorical mouse L1 characteristics. The total number of L1 sequences containing tested L1 characteristic (N) including the mean, SD, minimum, and maximum values of such characteristic were shown for compared L1 groups.

F subfamily

Total number of intragenic L1s = 332
Total number of intergenic L1s = 2270

Independent samples T-test

Section	Characteristic	Name	N	Mean	SD	Min	Max
Overall	Length	Intragenic L1s	332	10758.79	481.18	10000	12167
		Intergenic L1s	2270	10708.89	479.86	10001	12693
		t-statistic = 1.77, p-value = 7.70E-02					
	G-C Content	Intragenic L1s	332	39.29	1.49	34.58	43.62
		Intergenic L1s	2270	39.17	1.56	28.97	44.55
		t-statistic = 1.38, p-value = 1.66E-01					
	Intactness Score	Intragenic L1s	332	15.39	2.1	8	19
		Intergenic L1s	2270	15.21	2.22	5	19
		t-statistic = 1.44, p-value = 1.49E-01					
5' UTR	Number of monomers	Intragenic L1s	332	2.52	1.27	1	8
		Intergenic L1s	2270	2.59	1.32	1	23
		t-statistic = -0.83, p-value = 4.04E-01					
	Number of monomer splice sites	Intragenic L1s	332	10.74	4.67	0	27
		Intergenic L1s	2270	11.2	5.47	0	73
		t-statistic = -1.48, p-value = 1.40E-01					
ORF1	ORF1 gaps	Intragenic L1s	332	2.46	5.4	0	55
		Intergenic L1s	2270	1.97	5.28	0	71
		t-statistic = 1.58, p-value = 1.15E-01					
	ORF1 frameshifts	Intragenic L1s	332	2.06	1.52	0	8
		Intergenic L1s	2270	2.14	1.44	0	8
		t-statistic = -0.87, p-value = 3.83E-01					
	ORF1 stops	Intragenic L1s	332	2.02	1.83	0	14
		Intergenic L1s	2270	2.2	1.85	0	13
		t-statistic = -1.65, p-value = 9.84E-02					
ORF2	ORF2 gaps	Intragenic L1s	332	14.31	33.11	0	273
		Intergenic L1s	2270	14.46	34.78	0	376
		t-statistic = -0.07, p-value = 9.41E-01					
	ORF2 frameshifts	Intragenic L1s	332	7.89	3.67	0	22
		Intergenic L1s	2270	8.11	3.88	0	22
		t-statistic = -0.95, p-value = 3.43E-01					
	ORF2 stops	Intragenic L1s	332	8.89	5.38	0	32
		Intergenic L1s	2270	8.8	5.22	0	53
		t-statistic = 0.32, p-value = 7.49E-01					

A subfamily

Total number of intragenic L1s = 969
Total number of intergenic L1s = 5367

Independent samples T-test

Section	Characteristic	Name	N	Mean	SD	Min	Max
Overall	Length	Intragenic L1s	969	10818.01	586.18	9518	14423
		Intergenic L1s	5367	10792.27	555.88	9903	14893
		t-statistic = 1.32, p-value = 1.89E-01					
	G-C Content	Intragenic L1s	969	40.4	1.82	29.39	48.26
		Intergenic L1s	5367	40	1.82	20.37	49.93
		t-statistic = 6.28, p-value = 3.65E-10					
	Intactness Score	Intragenic L1s	969	17.51	2.21	8	19
		Intergenic L1s	5367	17.31	2.23	3	19
		t-statistic = 2.64, p-value = 8.35E-03					
5' UTR	Number of monomers	Intragenic L1s	969	3.18	1.98	1	23
		Intergenic L1s	5367	2.98	1.9	1	28
		t-statistic = 3.00, p-value = 2.75E-03					
	Number of monomer splice sites	Intragenic L1s	969	8.7	6.08	1	68
		Intergenic L1s	5367	8.07	5.83	1	83
		t-statistic = 3.05, p-value = 2.31E-03					
ORF1	ORF1 gaps	Intragenic L1s	969	0.87	3.15	0	37
		Intergenic L1s	5367	0.92	4.05	0	106
		t-statistic = -0.38, p-value = 7.03E-01					
	ORF1 frameshifts	Intragenic L1s	969	0.83	1.37	0	8
		Intergenic L1s	5367	0.9	1.32	0	8
		t-statistic = -1.54, p-value = 1.22E-01					
	ORF1 stops	Intragenic L1s	969	0.88	1.62	0	16
		Intergenic L1s	5367	0.95	1.59	0	20
		t-statistic = -1.27, p-value = 2.06E-01					
ORF2	ORF2 gaps	Intragenic L1s	969	6.73	26.88	0	358
		Intergenic L1s	5367	6.22	25.03	0	406
		t-statistic = 0.58, p-value = 5.63E-01					
	ORF2 frameshifts	Intragenic L1s	969	3.12	4.54	0	24
		Intergenic L1s	5367	3.59	4.47	0	24
		t-statistic = -2.97, p-value = 3.00E-03					
	ORF2 stops	Intragenic L1s	969	3.2	5.04	0	35
		Intergenic L1s	5367	3.82	5.28	0	63
		t-statistic = -3.35, p-value = 8.13E-04					

TF subfamily

Total number of intragenic L1s = 909
Total number of intergenic L1s = 4031

Independent samples T-test

Section	Characteristic	Name	N	Mean	SD	Min	Max
Overall	Length	Intragenic L1s	909	10812.04	571.06	10037	12898
		Intergenic L1s	4031	10818.59	570.35	9947	15111
		t-statistic = -0.31, p-value = 7.54E-01					
	G-C Content	Intragenic L1s	909	41.01	1.67	32.53	48.61
		Intergenic L1s	4031	40.72	1.7	27.94	47.94
		t-statistic = 4.63, p-value = 3.71E-06					
	Intactness Score	Intragenic L1s	909	18.82	0.59	14	19
		Intergenic L1s	4031	18.6	1	6	19
		t-statistic = 6.24, p-value = 4.68E-10					
5' UTR	Number of monomers	Intragenic L1s	909	3.73	1.7	1	11
		Intergenic L1s	4031	3.62	1.78	1	25
		t-statistic = 1.70, p-value = 8.86E-02					
	Number of monomer splice sites	Intragenic L1s	909	20.86	11.74	2	72
		Intergenic L1s	4031	19.92	12.12	0	170
		t-statistic = 2.13, p-value = 3.35E-02					
ORF1	ORF1 gaps	Intragenic L1s	909	0.2	2.84	0	59
		Intergenic L1s	4031	0.14	1.51	0	59
		t-statistic = 0.88, p-value = 3.80E-01					
	ORF1 frameshifts	Intragenic L1s	909	0.14	0.45	0	4
		Intergenic L1s	4031	0.24	0.58	0	6
		t-statistic = -4.78, p-value = 1.79E-06					
	ORF1 stops	Intragenic L1s	909	0.09	0.35	0	3
		Intergenic L1s	4031	0.19	0.52	0	6
		t-statistic = -5.09, p-value = 3.75E-07					
ORF2	ORF2 gaps	Intragenic L1s	909	0.78	7.34	0	125
		Intergenic L1s	4031	1.93	17.45	0	303
		t-statistic = -1.94, p-value = 5.22E-02					
	ORF2 frameshifts	Intragenic L1s	909	0.47	1.16	0	9
		Intergenic L1s	4031	0.86	1.71	0	15
		t-statistic = -6.55, p-value = 6.20E-11					
	ORF2 stops	Intragenic L1s	909	0.39	1.14	0	17
		Intergenic L1s	4031	0.74	1.67	0	27
		t-statistic = -5.87, p-value = 4.64E-09					

GF subfamily

Total number of intragenic L1s = 219
Total number of intergenic L1s = 1403

Independent samples T-test

Section	Characteristic	Name	N	Mean	SD	Min	Max
Overall	Length	Intragenic L1s	219	10806.82	544.05	10030	12450
		Intergenic L1s	1403	10742.63	526.29	10012	13508
		t-statistic = 1.67, p-value = 9.49E-02					
	G-C Content	Intragenic L1s	219	40.75	1.67	34.61	46.97
		Intergenic L1s	1403	40.29	1.78	27.42	46.84
		t-statistic = 3.58, p-value = 3.59E-04					
	Intactness Score	Intragenic L1s	219	18.34	1.19	11	19
		Intergenic L1s	1403	17.93	1.39	9	19
		t-statistic = 4.13, p-value = 3.73E-05					
5' UTR	Number of monomers	Intragenic L1s	219	3.69	1.93	1	11
		Intergenic L1s	1403	3.23	1.82	1	15
		t-statistic = 3.47, p-value = 5.38E-04					
	Number of monomer splice sites	Intragenic L1s	219	20.55	12.71	4	74
		Intergenic L1s	1403	16.97	11.9	1	97
		t-statistic = 4.11, p-value = 4.23E-05					
ORF1	ORF1 gaps	Intragenic L1s	219	0.26	1.11	0	8
		Intergenic L1s	1403	0.49	3.48	0	71
		t-statistic = -0.97, p-value = 3.30E-01					
	ORF1 frameshifts	Intragenic L1s	219	0.46	0.72	0	3
		Intergenic L1s	1403	0.6	0.85	0	6
		t-statistic = -2.35, p-value = 1.87E-02					
	ORF1 stops	Intragenic L1s	219	0.31	0.72	0	4
		Intergenic L1s	1403	0.55	0.9	0	6
		t-statistic = -3.80, p-value = 1.47E-04					
ORF2	ORF2 gaps	Intragenic L1s	219	1.66	9.56	0	127
		Intergenic L1s	1403	3.11	17.41	0	274
		t-statistic = -1.21, p-value = 2.28E-01					
	ORF2 frameshifts	Intragenic L1s	219	1.42	1.97	0	14
		Intergenic L1s	1403	2.35	2.45	0	19
		t-statistic = -5.35, p-value = 9.93E-08					
	ORF2 stops	Intragenic L1s	219	1.31	2.28	0	24
		Intergenic L1s	1403	2.27	2.63	0	20
		t-statistic = -5.11, p-value = 3.62E-07					

All L1 subfamilies

Total number of intragenic L1s = 2429
Total number of intergenic L1s = 13071

Independent samples T-test

Section	Characteristic	Name	N	Mean	SD	Min	Max
Overall	Length	Intragenic L1s	2429	10806.67	563.44	9518	14423
		Intergenic L1s	13071	10780.58	546.26	9903	15111
		t-statistic = 2.15, p-value = 3.15E-02					
	G-C Content	Intragenic L1s	2429	40.51	1.79	29.39	48.61
		Intergenic L1s	13071	40.11	1.81	20.37	49.93
		t-statistic = 9.96, p-value = 2.64E-23					
	Intactness Score	Intragenic L1s	2429	17.79	2.01	8	19
		Intergenic L1s	13071	17.41	2.17	3	19
		t-statistic = 7.94, p-value = 2.16E-15					
5' UTR	Number of monomers	Intragenic L1s	2429	3.34	1.83	1	23
		Intergenic L1s	13071	3.14	1.8	1	28
		t-statistic = 5.16, p-value = 2.45E-07					
	Number of monomer splice sites	Intragenic L1s	2429	14.6	10.84	0	74
		Intergenic L1s	13071	13.23	10.32	0	170
		t-statistic = 5.97, p-value = 2.45E-09					
ORF1	ORF1 gaps	Intragenic L1s	2429	0.78	3.41	0	59
		Intergenic L1s	13071	0.81	3.74	0	106
		t-statistic = -0.43, p-value = 6.66E-01					
	ORF1 frameshifts	Intragenic L1s	2429	0.7	1.25	0	8
		Intergenic L1s	13071	0.88	1.29	0	8
		t-statistic = -6.08, p-value = 1.24E-09					
	ORF1 stops	Intragenic L1s	2429	0.69	1.41	0	16
		Intergenic L1s	13071	0.89	1.51	0	20
		t-statistic = -6.03, p-value = 1.68E-09					
ORF2	ORF2 gaps	Intragenic L1s	2429	5.09	22.06	0	358
		Intergenic L1s	13071	6	24.74	0	406
		t-statistic = -1.69, p-value = 9.06E-02					
	ORF2 frameshifts	Intragenic L1s	2429	2.63	4.08	0	24
		Intergenic L1s	13071	3.4	4.28	0	24
		t-statistic = -8.19, p-value = 2.70E-16					
	ORF2 stops	Intragenic L1s	2429	2.76	4.75	0	35
		Intergenic L1s	13071	3.57	5.02	0	63
		t-statistic = -7.33, p-value = 2.46E-13					

Definitions of mouse L1 characteristics

Mouse L1 sequences were downloaded from L1Base [1]. These elements were annotated with important features for L1 activities. Some features in mouse L1 differ from that of human, e.g., monomer is only available in mouse. We group them according to where these features can be found, namely, 5' UTR, ORF1, ORF2 and 3' UTR. We put the overall features, e.g., G-C content and cannot be placed according to a specific location on L1 in a group called "Overall". Detailed information on finding of each feature can be found from the cited references. The measurement outputs are in two forms, categorical (e.g., conserved/mutated, for chi-square test) and non-categorical (e.g., %A, %T for student's *t*-test).

Overall

- ORF StartStop: check the presence of valid methionine start and stop codons in both ORF1 and ORF2 in the form of (M*, M*). This feature is reported as conserved, ORF1 conserved, ORF2 conserved, or mutated.
- Monomer Family [2]: classify mouse L1 families to F, A, T_F, and G_F, using the last monomer.
- CpG Islands: count the number of annotated CpG islands.
- G-C Content: calculate the percentage of G-C content of the L1 element in a 50nt-window.
- Intactness Score: calculate the overall score of categorical (conserved/mutated) features. Every intact feature (conserved) awards one point.

5' UTR

- SA-154 [3]: check the conservation of this splice site (see note below).
- Number of Monomers [2]: count the number of mouse L1 promoter monomers.
- Number of Monomer Splice Sites: count the number of monomer splice sites.

ORF1

- ORF1 conserved: check for the conservation of ORF1.
- 66/42 Monomers repeat [2]: check the pattern of the monomers in ORF1, such as 66-42-42 monomers.
- REKG235, ARR260, YPAKLS282 [4]: check for the intactness of amino acid residues at these particular loci (235, 260 and 282 positions, respectively, on the mouse L1 ORF1, see note below).
- SA+106, SA+120, SD+29, SD+52, SD+106, SD+288, SD+350 [3]: check for the conservation of these splice-sites at the respective positions (see note below).
- ORF1 gaps, ORF1 frameshifts, ORF1 stops: count the number of gaps, frameshifts and stop codons (TAA, TAG, TGA) in ORF1, respectively.

ORF2

- ORF2 conserved: check for the conservation of ORF2.

- N14, E43, Y115, D145, N147, T192, D205, SDH288 [5], R363 [6], FADD700 [5], HLKK1091, STS1096 [4], I1220, T1259 [6]: check for the intactness of amino acid residues at these particular loci (see note below).
- SA+1930, SA+4117, SD+1881, SD+2036, S_SA+1237, BG_SA+4578, BG_SD+4694, BG_SD+4903 [3]: check for the conservation of these splice-sites (see note below).
- ORF2 gaps, ORF2 frameshifts, ORF2 stops: count the number of gaps, frameshifts and stop codons (TAA, TAG, TGA) in ORF2.

3' UTR

- Poly A Signal [7]: check for the conservation of two poly-A patterns, namely 'AATAAA' or 'AATTAAA'.
- SD+5094, SA+5260, SA+5614 [3]: check for the conservation of these splice-sites (see note below).

Note

- **Splice site loci:** check for the conservation of mouse L1 splice-site **(SD**: splice donor, **SA**: splice acceptor, **BG_**: sites found in L1 inserted within an intron of the *beige* gene, **S_**: splice-site on the sense strand of L1 (without this, denotes the splice site on the antisense strand of L1), '**-**': represents nucleotide position backward from ORF1 start site, '**+**' : represents nucleotide position from ORF1 start site. The number followed the '+' or '-' represents the nucleotide position. [3]
- **Amino acid residues changes:** check for the intactness of amino acid residues on the ORF1 and ORF2. For example, **REKG235** in ORF1 refer to checking of the amino acid residues starting at 235 on the ORF1 start codon whether they match the sequence 'R-E-K-G', respectively. **N14** in ORF2 refer to checking the residue 'N' at the position 14 on the ORF2 start codon.

References

1. Penzkofer T, Dandekar T, Zemojtel T (2004) L1Base: from functional annotation to prediction of active LINE-1 elements. Nucl Acids Res 33: D498–D500.
2. Goodier JL, Ostertag EM, Du K, Kazazian HH Jr (2001) A novel active L1 retrotransposon subfamily in the mouse. Genome Res 11(10):1677-1685.
3. Zemojtel T, Penzkofer T, Schultz J, Dandekar T, Badge R, Vingron M (2007) Exonization of active mouse L1s: a driver of transcriptome evolution? BMC Genomics 8: 392.
4. Moran JV, Holmes SE, Naas TP, DeBerardinis RJ, Boeke JD and Kazazian HH Jr (1996) High frequency retrotransposition in cultured mammalian cells. Cell 87:917-927.
5. Feng Q, Moran JV, Kazazian HH Jr and Boeke JD (1996) Human L1 retrotransposon encodes a conserved endonuclease required for retrotransposition. Cell 87:905-916.
6. Lutz SM, Vincent BJ, Kazazian HH Jr, Batzer MA and Moran JV (2003) Allelic heterogeneity in LINE-1 retrotransposition activity. Am J Hum Genet 73:1431-1437.
7. Boeke JD (1997) LINEs and Alus--the polyA connection. Nat Genet 16:6-7.
