# Supplementary material for: Roles of Intragenic and Intergenic L1s in Mouse and Human
Source: PLoS One. 2014 Nov 19;9(11):e113434. doi: 10.1371/journal.pone.0113434 (PMC4237456; doi:10.1371/journal.pone.0113434)
Supplement: Table S2 — List of human L1 characteristics. (PDF) [file pone.0113434.s002.pdf]

Table 2.1 Mantel-Haenszel chi-square tests for human L1 characteristics

Mantel-Haenszel test is calculated by performing chi-square tests on different L1 strata (grouped by human subfamilies, L1PA and L1M). The total number of intragenic vs. intergenic L1s are shown in each column header. The Mantel-Haenszel correction results of each human L1 characteristic are shown in the last column, namely MH Odds Ratio, MH sum chisq, MH p-value, and MH 95% CI, respectively.

**L1PA subfamily**					**L1M subfamily**					**Mantel-Haenszel (MH) chi-square test**	
**Total number of intragenic L1s = 2332**<br>**Total number of intergenic L1s = 8336**					**Total number of intragenic L1s = 173**<br>**Total number of intergenic L1s = 796**						
**Overall**											
**ORF StartStop**											
Case	Intragenic L1s		Intergenic L1s		Case	Intragenic L1s		Intergenic L1s			
	Observed	Expected	Observed	Expected		Observed	Expected	Observed	Expected		
ORF1 Conserved	281	289.2	1042	1033.8	ORF1 Conserved	11	9.82	44	45.18	MH Odds Ratio	0.97
Other	2051	2042.8	7294	7302.2	Other	162	163.18	752	750.82	MH sum chisq	0.24
Odds Ratio = 0.96, 95% CI = 0.83 - 1.10					Odds Ratio = 1.16, 95% CI = 0.59 - 2.30					MH p-value	6.24E-01
Sum chisq = 0.34, p-value = 5.60E-01					Sum chisq = 0.18, p-value = 6.69E-01					MH 95% CI	0.84-1.11
ORF2 Conserved	475	498.18	1804	1780.82	ORF2 Conserved	9	11.25	54	51.75	MH Odds Ratio	0.92
Other	1857	1833.82	6532	6555.18	Other	164	161.75	742	744.25	MH sum chisq	2.05
Odds Ratio = 0.93, 95% CI = 0.83 - 1.04					Odds Ratio = 0.75, 95% CI = 0.36 - 1.56					MH p-value	1.52E-01
Sum chisq = 1.76, p-value = 1.85E-01					Sum chisq = 0.58, p-value = 4.44E-01					MH 95% CI	0.82-1.03
Conserved	1147	1035.28	3589	3700.72						MH Odds Ratio	1.28
Other	1185	1296.72	4747	4635.28						MH sum chisq	27.75
Odds Ratio = 1.28, 95% CI = 1.17 - 1.40										MH p-value	1.38E-07
Sum chisq = 27.75, p-value = 1.38E-07										MH 95% CI	1.17-1.40
Mutated	429	509.33	1901	1820.67	Mutated	153	150.68	691	693.32	MH Odds Ratio	0.78
Other	1903	1822.67	6435	6515.33	Other	20	22.32	105	102.68	MH sum chisq	18.61
Odds Ratio = 0.76, 95% CI = 0.68 - 0.86					Odds Ratio = 1.16, 95% CI = 0.70 - 1.93					MH p-value	1.61E-05
Sum chisq = 20.75, p-value = 5.24E-06					Sum chisq = 0.34, p-value = 5.62E-01					MH 95% CI	0.70-0.87
**find TSDs**											
Case	Intragenic L1s		Intergenic L1s		Case	Intragenic L1s		Intergenic L1s			
	Observed	Expected	Observed	Expected		Observed	Expected	Observed	Expected		
have tsds	1466	1419.14	5026	5072.86	have tsds	68	67.84	312	312.16	MH Odds Ratio	1.11
Other	866	912.86	3310	3263.14	Other	105	105.16	484	483.84	MH sum chisq	4.72
Odds Ratio = 1.11, 95% CI = 1.01 - 1.23					Odds Ratio = 1.00, 95% CI = 0.72 - 1.41					MH p-value	2.97E-02
Sum chisq = 5.06, p-value = 2.45E-02					Sum chisq = 0.00, p-value = 9.78E-01					MH 95% CI	1.01-1.21
no tsd	866	912.86	3310	3263.14	no tsd	105	105.16	484	483.84	MH Odds Ratio	0.90
Other	1466	1419.14	5026	5072.86	Other	68	67.84	312	312.16	MH sum chisq	4.72
Odds Ratio = 0.90, 95% CI = 0.82 - 0.99					Odds Ratio = 1.00, 95% CI = 0.71 - 1.39					MH p-value	2.97E-02
Sum chisq = 5.06, p-value = 2.45E-02					Sum chisq = 0.00, p-value = 9.78E-01					MH 95% CI	0.82-0.99
**CPG Islands**											
Case	Intragenic L1s		Intergenic L1s		Case	Intragenic L1s		Intergenic L1s			
	Observed	Expected	Observed	Expected		Observed	Expected	Observed	Expected		
0 island	1961	2013.28	7249	7196.72	0 island	170	168.36	773	774.64	MH Odds Ratio	0.80
Other	371	318.72	1087	1139.28	Other	3	4.64	23	21.36	MH sum chisq	11.72
Odds Ratio = 0.79, 95% CI = 0.70 - 0.90					Odds Ratio = 1.69, 95% CI = 0.50 - 5.68					MH p-value	6.17E-04
Sum chisq = 12.71, p-value = 3.63E-04					Sum chisq = 0.35, p-value = 5.53E-01					MH 95% CI	0.71-0.91
>=1 island(s)	371	318.72	1087	1139.28	>=1 island(s)	3	4.64	23	21.36	MH Odds Ratio	1.25
Other	1961	2013.28	7249	7196.72	Other	170	168.36	773	774.64	MH sum chisq	11.72
Odds Ratio = 1.26, 95% CI = 1.11 - 1.43					Odds Ratio = 0.59, 95% CI = 0.18 - 2.00					MH p-value	6.17E-04
Sum chisq = 12.71, p-value = 3.63E-04					Sum chisq = 0.35, p-value = 5.53E-01					MH 95% CI	1.10-1.42
**5' UTR**											
**Ta1-nd/d**											
Case	Intragenic L1s		Intergenic L1s		Case	Intragenic L1s		Intergenic L1s			
	Observed	Expected	Observed	Expected		Observed	Expected	Observed	Expected		
Ta1-d	208	247.45	924	884.55	Ta1-d	27	29.82	140	137.18	MH Odds Ratio	0.79
Other	2124	2084.55	7412	7451.45	Other	146	143.18	656	658.82	MH sum chisq	9.25
Odds Ratio = 0.79, 95% CI = 0.67 - 0.92					Odds Ratio = 0.87, 95% CI = 0.55 - 1.36					MH p-value	2.35E-03
Sum chisq = 9.01, p-value = 2.69E-03					Sum chisq = 0.39, p-value = 5.32E-01					MH 95% CI	0.68-0.92
Ta1-nd	1225	1162.5	4093	4155.5	Ta1-nd	33	27.49	121	126.51	MH Odds Ratio	1.15
Other	1107	1169.5	4243	4180.5	Other	140	145.51	675	669.49	MH sum chisq	9.75
Odds Ratio = 1.15, 95% CI = 1.05 - 1.26					Odds Ratio = 1.31, 95% CI = 0.86 - 2.01					MH p-value	1.80E-03
Sum chisq = 8.57, p-value = 3.41E-03					Sum chisq = 1.60, p-value = 2.07E-01					MH 95% CI	1.05-1.26
non canonical	899	922.04	3319	3295.96	non canonical	113	115.69	535	532.31	MH Odds Ratio	0.95
Other	1433	1409.96	5017	5040.04	Other	60	57.31	261	263.69	MH sum chisq	1.42
Odds Ratio = 0.95, 95% CI = 0.86 - 1.04					Odds Ratio = 0.92, 95% CI = 0.65 - 1.30					MH p-value	2.34E-01
Sum chisq = 1.22, p-value = 2.70E-01					Sum chisq = 0.23, p-value = 6.32E-01					MH 95% CI	0.86-1.04
**Runx3 Site**											
Case	Intragenic L1s		Intergenic L1s		Case	Intragenic L1s		Intergenic L1s			
	Observed	Expected	Observed	Expected		Observed	Expected	Observed	Expected		
Conserved	585	548.68	1925	1961.32						MH Odds Ratio	1.12
Other	1747	1783.32	6411	6374.68						MH sum chisq	4.02
Odds Ratio = 1.12, 95% CI = 1.00 - 1.24										MH p-value	4.49E-02
Sum chisq = 4.02, p-value = 4.49E-02										MH 95% CI	1.00-1.24
Mutated	1747	1783.32	6411	6374.68	Mutated	173	172.11	791	791.89	MH Odds Ratio	0.90
Other	585	548.68	1925	1961.32	Other	0	0.89	5	4.11	MH sum chisq	3.82
Odds Ratio = 0.90, 95% CI = 0.81 - 1.00					Odds Ratio = -, 95% CI = -					MH p-value	5.07E-02
Sum chisq = 4.02, p-value = 4.49E-02					Sum chisq = 0.21, p-value = 6.46E-01					MH 95% CI	-
**Runx3 ASP**											
Case	Intragenic L1s		Intergenic L1s		Case	Intragenic L1s		Intergenic L1s			
	Observed	Expected	Observed	Expected		Observed	Expected	Observed	Expected		
Conserved	713	664.76	2328	2376.24	Conserved	1	1.07	5	4.93	MH Odds Ratio	1.14
Other	1619	1667.24	6008	5959.76	Other	172	171.93	791	791.07	MH sum chisq	6.23
Odds Ratio = 1.14, 95% CI = 1.03 - 1.26					Odds Ratio = 0.92, 95% CI = 0.11 - 7.92					MH p-value	1.25E-02
Sum chisq = 6.27, p-value = 1.23E-02					Sum chisq = 0.21, p-value = 6.47E-01					MH 95% CI	1.03-1.26
Mutated	1619	1667.24	6008	5959.76	Mutated	172	171.93	791	791.07	MH Odds Ratio	0.88
Other	713	664.76	2328	2376.24	Other	1	1.07	5	4.93	MH sum chisq	6.23
Odds Ratio = 0.88, 95% CI = 0.80 - 0.97					Odds Ratio = 1.09, 95% CI = 0.13 - 9.37					MH p-value	1.25E-02
Sum chisq = 6.27, p-value = 1.23E-02					Sum chisq = 0.21, p-value = 6.47E-01					MH 95% CI	0.80-0.97
**SRY Site 1**											
Case	Intragenic L1s		Intergenic L1s		Case	Intragenic L1s		Intergenic L1s			
	Observed	Expected	Observed	Expected		Observed	Expected	Observed	Expected		
Conserved	1501	1428.1	5032	5104.9	Conserved	15	16.25	76	74.75	MH Odds Ratio	1.18
Other	831	903.9	3304	3231.1	Other	158	156.75	720	721.25	MH sum chisq	11.54
Odds Ratio = 1.19, 95% CI = 1.08 - 1.30					Odds Ratio = 0.90, 95% CI = 0.50 - 1.61					MH p-value	6.79E-04
Sum chisq = 12.29, p-value = 4.56E-04					Sum chisq = 0.13, p-value = 7.20E-01					MH 95% CI	1.07-1.29
Mutated	831	903.9	3304	3231.1	Mutated	158	156.75	720	721.25	MH Odds Ratio	0.85
Other	1501	1428.1	5032	5104.9	Other	15	16.25	76	74.75	MH sum chisq	11.54
Odds Ratio = 0.84, 95% CI = 0.77 - 0.93					Odds Ratio = 1.11, 95% CI = 0.62 - 1.99					MH p-value	6.79E-04
Sum chisq = 12.29, p-value = 4.56E-04					Sum chisq = 0.13, p-value = 7.20E-01					MH 95% CI	0.77-0.93
**SRY Site 2**											
Case	Intragenic L1s		Intergenic L1s		Case	Intragenic L1s		Intergenic L1s			
	Observed	Expected	Observed	Expected		Observed	Expected	Observed	Expected		
Conserved	919	887.94	3143	3174.06	Conserved	28	25.53	115	117.47	MH Odds Ratio	1.08
Other	1413	1444.06	5193	5161.94	Other	145	147.47	681	678.53	MH sum chisq	2.51
Odds Ratio = 1.07, 95% CI = 0.98 - 1.18					Odds Ratio = 1.14, 95% CI = 0.73 - 1.79					MH p-value	1.13E-01
Sum chisq = 2.24, p-value = 1.34E-01					Sum chisq = 0.34, p-value = 5.59E-01					MH 95% CI	0.98-1.18
Mutated	1413	1444.06	5193	5161.94	Mutated	145	147.47	681	678.53	MH Odds Ratio	0.93
Other	919	887.94	3143	3174.06	Other	28	25.53	115	117.47	MH sum chisq	2.51
Odds Ratio = 0.93, 95% CI = 0.85 - 1.02					Odds Ratio = 0.87, 95% CI = 0.56 - 1.37					MH p-value	1.13E-01
Sum chisq = 2.24, p-value = 1.34E-01					Sum chisq = 0.34, p-value = 5.59E-01					MH 95% CI	0.85-1.02

YY1 BoxA+BoxA

Case	Intragenic L1s		Intergenic L1s		Case	Intragenic L1s		Intergenic L1s			
	Observed	Expected	Observed	Expected		Observed	Expected	Observed	Expected		
Conserved	502	451.19	1562	1612.81						MH Odds Ratio	1.19
Other	1830	1880.81	6774	6723.19						MH sum chisq	9.08
Odds Ratio = 1.19, 95% CI = 1.06 - 1.33										MH p-value	2.58E-03
Sum chisq = 9.08, p-value = 2.58E-03										MH 95% CI	1.06-1.33
Mutated	1830	1880.81	6774	6723.19	Mutated	173	172.82	795	795.18	MH Odds Ratio	0.84
Other	502	451.19	1562	1612.81	Other	0	0.18	1	0.82	MH sum chisq	9.01
Odds Ratio = 0.84, 95% CI = 0.75 - 0.94					Odds Ratio = -, 95% CI = -					MH p-value	2.69E-03
Sum chisq = 9.08, p-value = 2.58E-03					Sum chisq = 0.71, p-value = 4.01E-01					MH 95% CI	-

TF nkx-2.5

Case	Intragenic L1s		Intergenic L1s		Case	Intragenic L1s		Intergenic L1s			
	Observed	Expected	Observed	Expected		Observed	Expected	Observed	Expected		
Conserved	1566	1454.33	5087	5198.67	Conserved	13	12.68	58	58.32	MH Odds Ratio	1.30
Other	766	877.67	3249	3137.33	Other	160	160.32	738	737.68	MH sum chisq	28.67
Odds Ratio = 1.31, 95% CI = 1.19 - 1.44					Odds Ratio = 1.03, 95% CI = 0.55 - 1.93					MH p-value	8.57E-08
Sum chisq = 29.16, p-value = 6.68E-08					Sum chisq = 0.01, p-value = 9.17E-01					MH 95% CI	1.18 -1.43
Mutated	766	877.67	3249	3137.33	Mutated	160	160.32	738	737.68	MH Odds Ratio	0.77
Other	1566	1454.33	5087	5198.67	Other	13	12.68	58	58.32	MH sum chisq	28.67
Odds Ratio = 0.77, 95% CI = 0.70 - 0.84					Odds Ratio = 0.97, 95% CI = 0.52 - 1.81					MH p-value	8.57E-08
Sum chisq = 29.16, p-value = 6.68E-08					Sum chisq = 0.01, p-value = 9.17E-01					MH 95% CI	0.70-0.85

TF nkx-2.5B

Case	Intragenic L1s		Intergenic L1s		Case	Intragenic L1s		Intergenic L1s			
	Observed	Expected	Observed	Expected		Observed	Expected	Observed	Expected		
Conserved	203	175.75	601	628.25	Conserved	5	4.11	18	18.89	MH Odds Ratio	1.23
Other	2129	2156.25	7735	7707.75	Other	168	168.89	778	777.11	MH sum chisq	6.08
Odds Ratio = 1.23, 95% CI = 1.04 - 1.45					Odds Ratio = 1.29, 95% CI = 0.47 - 3.51					MH p-value	1.37E-02
Sum chisq = 5.85, p-value = 1.56E-02					Sum chisq = 0.05, p-value = 8.28E-01					MH 95% CI	1.04-1.45
Mutated	2129	2156.25	7735	7707.75	Mutated	168	168.89	778	777.11	MH Odds Ratio	0.81
Other	203	175.75	601	628.25	Other	5	4.11	18	18.89	MH sum chisq	6.08
Odds Ratio = 0.81, 95% CI = 0.69 - 0.96					Odds Ratio = 0.78, 95% CI = 0.28 - 2.12					MH p-value	1.37E-02
Sum chisq = 5.85, p-value = 1.56E-02					Sum chisq = 0.05, p-value = 8.28E-01					MH 95% CI	0.69-0.96

ORF1

ORF1 Conserved

Case	Intragenic L1s		Intergenic L1s		Case	Intragenic L1s		Intergenic L1s			
	Observed	Expected	Observed	Expected		Observed	Expected	Observed	Expected		
Conserved	2331	2331.78	8336	8335.22	Conserved	167	171.93	796	791.07	MH Odds Ratio	0.00
Other	1	0.22	0	0.78	Other	6	1.07	0	4.93	MH sum chisq	31.16
Odds Ratio = 0.00, 95% CI = -					Odds Ratio = 0.00, 95% CI = -					MH p-value	2.37E-08
Sum chisq = 0.46, p-value = 4.96E-01					Sum chisq = 22.43, p-value = 2.18E-06					MH 95% CI	-

REKG235

Case	Intragenic L1s		Intergenic L1s		Case	Intragenic L1s		Intergenic L1s			
	Observed	Expected	Observed	Expected		Observed	Expected	Observed	Expected		
Conserved	1656	1564.28	5500	5591.72	Conserved	9	12.32	60	56.68	MH Odds Ratio	1.25
Other	676	767.72	2836	2744.28	Other	164	160.68	736	739.32	MH sum chisq	18.97
Odds Ratio = 1.26, 95% CI = 1.14 - 1.40					Odds Ratio = 0.67, 95% CI = 0.33 - 1.38					MH p-value	1.32E-05
Sum chisq = 20.90, p-value = 4.83E-06					Sum chisq = 1.17, p-value = 2.79E-01					MH 95% CI	1.13-1.38
Mutated	675	764.87	2824	2734.13	Mutated	158	151.04	688	694.96	MH Odds Ratio	0.82
Other	1657	1567.13	5512	5601.87	Other	15	21.96	108	101.04	MH sum chisq	16.47
Odds Ratio = 0.80, 95% CI = 0.72 - 0.88					Odds Ratio = 1.65, 95% CI = 0.94 - 2.92					MH p-value	4.95E-05
Sum chisq = 20.11, p-value = 7.31E-06					Sum chisq = 3.08, p-value = 7.95E-02					MH 95% CI	0.74-0.90

ARR260

Case	Intragenic L1s		Intergenic L1s		Case	Intragenic L1s		Intergenic L1s			
	Observed	Expected	Observed	Expected		Observed	Expected	Observed	Expected		
Conserved	1927	1809.11	6349	6466.89	Conserved	73	64.63	289	297.37	MH Odds Ratio	1.47
Other	405	522.89	1987	1869.11	Other	100	108.37	507	498.63	MH sum chisq	45.51
Odds Ratio = 1.49, 95% CI = 1.32 - 1.68					Odds Ratio = 1.28, 95% CI = 0.92 - 1.79					MH p-value	1.52E-11
Sum chisq = 43.84, p-value = 3.56E-11					Sum chisq = 2.11, p-value = 1.47E-01					MH 95% CI	1.31-1.64
Mutated	404	520.04	1975	1858.96	Mutated	94	98.73	459	454.27	MH Odds Ratio	0.69
Other	1928	1811.96	6361	6477.04	Other	79	74.27	337	341.73	MH sum chisq	41.60
Odds Ratio = 0.67, 95% CI = 0.60 - 0.76					Odds Ratio = 0.87, 95% CI = 0.63 - 1.22					MH p-value	1.12E-10
Sum chisq = 42.65, p-value = 6.55E-11					Sum chisq = 0.64, p-value = 4.23E-01					MH 95% CI	0.62-0.78

YPAKLS282

Case	Intragenic L1s		Intergenic L1s		Case	Intragenic L1s		Intergenic L1s			
	Observed	Expected	Observed	Expected		Observed	Expected	Observed	Expected		
Conserved	1561	1441.43	5033	5152.57	Conserved	13	13.75	64	63.25	MH Odds Ratio	1.32
Other	771	890.57	3303	3183.43	Other	160	159.25	732	732.75	MH sum chisq	32.04
Odds Ratio = 1.33, 95% CI = 1.21 - 1.46					Odds Ratio = 0.93, 95% CI = 0.50 - 1.73					MH p-value	1.51E-08
Sum chisq = 33.24, p-value = 8.16E-09					Sum chisq = 0.05, p-value = 8.17E-01					MH 95% CI	1.20-1.45
Mutated	770	887.73	3291	3173.27	Mutated	154	149.61	684	688.39	MH Odds Ratio	0.77
Other	1562	1444.27	5045	5162.73	Other	19	23.39	112	107.61	MH sum chisq	28.78
Odds Ratio = 0.76, 95% CI = 0.69 - 0.83					Odds Ratio = 1.33, 95% CI = 0.79 - 2.23					MH p-value	8.09E-08
Sum chisq = 32.26, p-value = 1.35E-08					Sum chisq = 1.16, p-value = 2.82E-01					MH 95% CI	0.70-0.85

ORF2

ORF2 Conserved

Case	Intragenic L1s		Intergenic L1s		Case	Intragenic L1s		Intergenic L1s			
	Observed	Expected	Observed	Expected		Observed	Expected	Observed	Expected		
Conserved	2332	2332	8336	8336	Conserved	173	173	796	796	MH Odds Ratio	-
Other	0	0	0	0	Other	0	0	0	0	MH sum chisq	-
Odds Ratio = -, 95% CI = -					Odds Ratio = -, 95% CI = -					MH p-value	-
Sum chisq = -, p-value = -					Sum chisq = -, p-value = -					MH 95% CI	-

Ta0/Ta1 SSVs

Case	Intragenic L1s		Intergenic L1s		Case	Intragenic L1s		Intergenic L1s			
	Observed	Expected	Observed	Expected		Observed	Expected	Observed	Expected		
L1PA5	1078	984.13	3424	3517.87	L1PA5	12	12.5	58	57.5	MH Odds Ratio	1.23
Other	1254	1347.87	4912	4818.13	Other	161	160.5	738	738.5	MH sum chisq	19.20
Odds Ratio = 1.23, 95% CI = 1.12 - 1.35					Odds Ratio = 0.95, 95% CI = 0.50 - 1.81					MH p-value	1.18E-05
Sum chisq = 19.83, p-value = 8.48E-06					Sum chisq = 0.03, p-value = 8.72E-01					MH 95% CI	1.12-1.34
Ta-0/L1PA2	650	628.91	2227	2248.09	Ta-0/L1PA2	13	12.68	58	58.32	MH Odds Ratio	1.06
Other	1682	1703.09	6109	6087.91	Other	160	160.32	738	737.68	MH sum chisq	1.24
Odds Ratio = 1.06, 95% CI = 0.96 - 1.17					Odds Ratio = 1.03, 95% CI = 0.55 - 1.93					MH p-value	2.65E-01
Sum chisq = 1.24, p-value = 2.66E-01					Sum chisq = 0.01, p-value = 9.17E-01					MH 95% CI	0.96-1.17
Ta-1	56	49.62	171	177.38	Ta-1	11	13.21	63	60.79	MH Odds Ratio	1.09
Other	2276	2282.38	8165	8158.62	Other	162	159.79	733	735.21	MH sum chisq	0.36
Odds Ratio = 1.17, 95% CI = 0.87 - 1.59					Odds Ratio = 0.79, 95% CI = 0.41 - 1.53					MH p-value	5.47E-01
Sum chisq = 1.07, p-value = 3.00E-01					Sum chisq = 0.49, p-value = 4.85E-01					MH 95% CI	0.82-1.44
non canonical	548	669.35	2514	2392.65	non canonical	137	134.62	617	619.38	MH Odds Ratio	0.73
Other	1784	1662.65	5822	5943.35	Other	36	38.38	179	176.62	MH sum chisq	35.61
Odds Ratio = 0.71, 95% CI = 0.64 - 0.79					Odds Ratio = 1.10, 95% CI = 0.74 - 1.65					MH p-value	2.41E-09
Sum chisq = 39.49, p-value = 3.30E-10					Sum chisq = 0.23, p-value = 6.30E-01					MH 95% CI	0.66-0.81

N14

Case	Intragenic L1s		Intergenic L1s		Case	Intragenic L1s		Intergenic L1s			
	Observed	Expected	Observed	Expected		Observed	Expected	Observed	Expected		
Conserved	2169	2151.22	7672	7689.78	Conserved	134	137.65	637	633.35	MH Odds Ratio	1.10
Other	163	180.78	664	646.22	Other	39	35.35	159	162.65	MH sum chisq	#REF!
Odds Ratio = 1.15, 95% CI = 0.96 - 1.38					Odds Ratio = 0.86, 95% CI = 0.58 - 1.28					MH p-value	#REF!
Sum chisq = 2.43, p-value = 1.19E-01					Sum chisq = 0.58, p-value = 4.48E-01					MH 95% CI	0.93-1.29
Mutated	163	180.78	664	646.22	Mutated	39	35.17	158	161.83	MH Odds Ratio	0.91
Other	2169	2151.22	7672	7689.78	Other	134	137.83	638	634.17	MH sum chisq	#REF!
Odds Ratio = 0.87, 95% CI = 0.73 - 1.04					Odds Ratio = 1.18, 95% CI = 0.79 - 1.75					MH p-value	#REF!

Sum chisq = 2.43, p-value = 1.19E-01					Sum chisq = 0.64, p-value = 4.25E-01					MH 95% CI	0.77-1.07

E43

Case	Intragenic L1s		Intergenic L1s		Case	Intragenic L1s		Intergenic L1s			
	Observed	Expected	Observed	Expected		Observed	Expected	Observed	Expected		
Conserved	2163	2135.26	7605	7632.74	Conserved	118	122.3	567	562.7	MH Odds Ratio	1.15
Other	169	196.74	731	703.26	Other	55	50.7	229	233.3	MH sum chisq	#REF!
Odds Ratio = 1.23, 95% CI = 1.03 - 1.46					Odds Ratio = 0.87, 95% CI = 0.61 - 1.24					MH p-value	#REF!
Sum chisq = 5.47, p-value = 1.94E-02					Sum chisq = 0.63, p-value = 4.29E-01					MH 95% CI	0.99-1.35
Mutated	169	196.74	731	703.26	Mutated	55	50.53	228	232.47	MH Odds Ratio	0.87
Other	2163	2135.26	7605	7632.74	Other	118	122.47	568	563.53	MH sum chisq	#REF!
Odds Ratio = 0.81, 95% CI = 0.68 - 0.97					Odds Ratio = 1.16, 95% CI = 0.81 - 1.66					MH p-value	#REF!
Sum chisq = 5.47, p-value = 1.94E-02					Sum chisq = 0.68, p-value = 4.09E-01					MH 95% CI	0.74-1.02

Y115

Case	Intragenic L1s		Intergenic L1s		Case	Intragenic L1s		Intergenic L1s			
	Observed	Expected	Observed	Expected		Observed	Expected	Observed	Expected		
Conserved	2131	2104.22	7495	7521.78	Conserved	121	128.72	600	592.28	MH Odds Ratio	1.11
Other	201	227.78	841	814.22	Other	52	44.28	196	203.72	MH sum chisq	#REF!
Odds Ratio = 1.19, 95% CI = 1.01 - 1.40					Odds Ratio = 0.76, 95% CI = 0.53 - 1.09					MH p-value	#REF!
Sum chisq = 4.47, p-value = 3.46E-02					Sum chisq = 2.20, p-value = 1.38E-01					MH 95% CI	0.96-1.29
Mutated	201	227.78	841	814.22	Mutated	52	44.1	195	202.9	MH Odds Ratio	0.90
Other	2131	2104.22	7495	7521.78	Other	121	128.9	601	593.1	MH sum chisq	#REF!
Odds Ratio = 0.84, 95% CI = 0.72 - 0.99					Odds Ratio = 1.32, 95% CI = 0.92 - 1.90					MH p-value	#REF!
Sum chisq = 4.47, p-value = 3.46E-02					Sum chisq = 2.31, p-value = 1.28E-01					MH 95% CI	0.78-1.05

D145

Case	Intragenic L1s		Intergenic L1s		Case	Intragenic L1s		Intergenic L1s			
	Observed	Expected	Observed	Expected		Observed	Expected	Observed	Expected		
Conserved	2106	2050.23	7273	7328.77	Conserved	119	117.48	539	540.52	MH Odds Ratio	1.31
Other	226	281.77	1063	1007.23	Other	54	55.52	257	255.48	MH sum chisq	#REF!
Odds Ratio = 1.36, 95% CI = 1.17 - 1.58					Odds Ratio = 1.05, 95% CI = 0.74 - 1.50					MH p-value	#REF!
Sum chisq = 16.07, p-value = 6.11E-05					Sum chisq = 0.08, p-value = 7.84E-01					MH 95% CI	1.14-1.51
Mutated	226	281.77	1063	1007.23	Mutated	54	55.35	256	254.65	MH Odds Ratio	0.76
Other	2106	2050.23	7273	7328.77	Other	119	117.65	540	541.35	MH sum chisq	#REF!
Odds Ratio = 0.73, 95% CI = 0.63 - 0.85					Odds Ratio = 0.96, 95% CI = 0.67 - 1.36					MH p-value	#REF!
Sum chisq = 16.07, p-value = 6.11E-05					Sum chisq = 0.06, p-value = 8.09E-01					MH 95% CI	0.66-0.88

N147

Case	Intragenic L1s		Intergenic L1s		Case	Intragenic L1s		Intergenic L1s			
	Observed	Expected	Observed	Expected		Observed	Expected	Observed	Expected		
Conserved	2177	2135.92	7594	7635.08	Conserved	131	131.4	605	604.6	MH Odds Ratio	1.30
Other	155	196.08	742	700.92	Other	42	41.6	191	191.4	MH sum chisq	#REF!
Odds Ratio = 1.37, 95% CI = 1.15 - 1.64					Odds Ratio = 0.98, 95% CI = 0.67 - 1.45					MH p-value	#REF!
Sum chisq = 12.03, p-value = 5.25E-04					Sum chisq = 0.01, p-value = 9.37E-01					MH 95% CI	1.10-1.53
Mutated	155	196.08	742	700.92	Mutated	42	41.42	190	190.58	MH Odds Ratio	0.77
Other	2177	2135.92	7594	7635.08	Other	131	131.58	606	605.42	MH sum chisq	#REF!
Odds Ratio = 0.73, 95% CI = 0.61 - 0.87					Odds Ratio = 1.02, 95% CI = 0.70 - 1.50					MH p-value	#REF!
Sum chisq = 12.03, p-value = 5.25E-04					Sum chisq = 0.01, p-value = 9.09E-01					MH 95% CI	0.66-0.91

T192

Case	Intragenic L1s		Intergenic L1s		Case	Intragenic L1s		Intergenic L1s			
	Observed	Expected	Observed	Expected		Observed	Expected	Observed	Expected		
Conserved	2149	2113.62	7520	7555.38	Conserved	114	121.05	564	556.95	MH Odds Ratio	1.17
Other	183	218.38	816	780.62	Other	59	51.95	232	239.05	MH sum chisq	#REF!
Odds Ratio = 1.27, 95% CI = 1.08 - 1.51					Odds Ratio = 0.79, 95% CI = 0.56 - 1.13					MH p-value	#REF!
Sum chisq = 8.09, p-value = 4.44E-03					Sum chisq = 1.66, p-value = 1.97E-01					MH 95% CI	1.01-1.37
Mutated	183	218.38	816	780.62	Mutated	59	51.78	231	238.22	MH Odds Ratio	0.85
Other	2149	2113.62	7520	7555.38	Other	114	121.22	565	557.78	MH sum chisq	#REF!
Odds Ratio = 0.78, 95% CI = 0.66 - 0.93					Odds Ratio = 1.27, 95% CI = 0.89 - 1.80					MH p-value	#REF!
Sum chisq = 8.09, p-value = 4.44E-03					Sum chisq = 1.75, p-value = 1.86E-01					MH 95% CI	0.73-0.99

D205

Case	Intragenic L1s		Intergenic L1s		Case	Intragenic L1s		Intergenic L1s			
	Observed	Expected	Observed	Expected		Observed	Expected	Observed	Expected		
Conserved	2092	2021.15	7154	7224.85	Conserved	116	110.33	502	507.67	MH Odds Ratio	1.40
Other	240	310.85	1182	1111.15	Other	57	62.67	294	288.33	MH sum chisq	#REF!
Odds Ratio = 1.44, 95% CI = 1.24 - 1.67					Odds Ratio = 1.19, 95% CI = 0.84 - 1.69					MH p-value	#REF!
Sum chisq = 23.84, p-value = 1.05E-06					Sum chisq = 0.98, p-value = 3.23E-01					MH 95% CI	1.22-1.61
Mutated	240	310.85	1182	1111.15	Mutated	57	62.49	293	287.51	MH Odds Ratio	0.71
Other	2092	2021.15	7154	7224.85	Other	116	110.51	503	508.49	MH sum chisq	#REF!
Odds Ratio = 0.69, 95% CI = 0.60 - 0.80					Odds Ratio = 0.84, 95% CI = 0.60 - 1.19					MH p-value	#REF!
Sum chisq = 23.84, p-value = 1.05E-06					Sum chisq = 0.92, p-value = 3.38E-01					MH 95% CI	0.62-0.82

SDH228

Case	Intragenic L1s		Intergenic L1s		Case	Intragenic L1s		Intergenic L1s			
	Observed	Expected	Observed	Expected		Observed	Expected	Observed	Expected		
Conserved	1873	1793.59	6332	6411.41	Conserved	68	64.45	293	296.55	MH Odds Ratio	1.27
Other	459	538.41	2004	1924.59	Other	105	108.55	503	499.45	MH sum chisq	#REF!
Odds Ratio = 1.29, 95% CI = 1.15 - 1.45					Odds Ratio = 1.11, 95% CI = 0.79 - 1.56					MH p-value	#REF!
Sum chisq = 19.49, p-value = 1.01E-05					Sum chisq = 0.38, p-value = 5.38E-01					MH 95% CI	1.14-1.42
Mutated	459	538.41	2004	1924.59	Mutated	105	108.37	502	498.63	MH Odds Ratio	0.79
Other	1873	1793.59	6332	6411.41	Other	68	64.63	294	297.37	MH sum chisq	#REF!
Odds Ratio = 0.77, 95% CI = 0.69 - 0.87					Odds Ratio = 0.90, 95% CI = 0.65 - 1.27					MH p-value	#REF!
Sum chisq = 19.49, p-value = 1.01E-05					Sum chisq = 0.34, p-value = 5.59E-01					MH 95% CI	0.71-0.88

R363

Case	Intragenic L1s		Intergenic L1s		Case	Intragenic L1s		Intergenic L1s			
	Observed	Expected	Observed	Expected		Observed	Expected	Observed	Expected		
Conserved	1880	1789.88	6308	6398.12	Conserved	21	24.82	118	114.18	MH Odds Ratio	1.30
Other	452	542.12	2028	1937.88	Other	152	148.18	678	681.82	MH sum chisq	#REF!
Odds Ratio = 1.34, 95% CI = 1.19 - 1.50					Odds Ratio = 0.79, 95% CI = 0.48 - 1.30					MH p-value	#REF!
Sum chisq = 24.98, p-value = 5.79E-07					Sum chisq = 0.83, p-value = 3.61E-01					MH 95% CI	1.16-1.46
Mutated	452	542.12	2028	1937.88	Mutated	152	148.01	677	680.99	MH Odds Ratio	0.77
Other	1880	1789.88	6308	6398.12	Other	21	24.99	119	115.01	MH sum chisq	#REF!
Odds Ratio = 0.75, 95% CI = 0.67 - 0.84					Odds Ratio = 1.27, 95% CI = 0.77 - 2.09					MH p-value	#REF!
Sum chisq = 24.98, p-value = 5.79E-07					Sum chisq = 0.91, p-value = 3.41E-01					MH 95% CI	0.69-0.86

FADD700

Case	Intragenic L1s		Intergenic L1s		Case	Intragenic L1s		Intergenic L1s			
	Observed	Expected	Observed	Expected		Observed	Expected	Observed	Expected		
Conserved	1695	1626.15	5744	5812.85	Conserved	45	42.13	191	193.87	MH Odds Ratio	1.19
Other	637	705.85	2592	2523.15	Other	128	130.87	605	602.13	MH sum chisq	#REF!
Odds Ratio = 1.20, 95% CI = 1.08 - 1.33					Odds Ratio = 1.11, 95% CI = 0.76 - 1.62					MH p-value	#REF!
Sum chisq = 12.33, p-value = 4.47E-04					Sum chisq = 0.31, p-value = 5.75E-01					MH 95% CI	1.08-1.32
Mutated	637	705.85	2592	2523.15	Mutated	128	130.69	604	601.31	MH Odds Ratio	0.84
Other	1695	1626.15	5744	5812.85	Other	45	42.31	192	194.69	MH sum chisq	#REF!
Odds Ratio = 0.83, 95% CI = 0.75 - 0.92					Odds Ratio = 0.90, 95% CI = 0.62 - 1.32					MH p-value	#REF!
Sum chisq = 12.33, p-value = 4.47E-04					Sum chisq = 0.28, p-value = 6.00E-01					MH 95% CI	0.76-0.92

HMKK1091

Case	Intragenic L1s		Intergenic L1s		Case	Intragenic L1s		Intergenic L1s			
	Observed	Expected	Observed	Expected		Observed	Expected	Observed	Expected		
Conserved	1569	1465.48	5135	5238.52	Conserved	6	6.43	30	29.57	MH Odds Ratio	1.28
Other	763	866.52	3201	3097.48	Other	167	166.57	766	766.43	MH sum chisq	#REF!
Odds Ratio = 1.28, 95% CI = 1.16 - 1.41					Odds Ratio = 0.92, 95% CI = 0.38 - 2.24					MH p-value	#REF!
Sum chisq = 25.19, p-value = 5.21E-07					Sum chisq = 0.04, p-value = 8.50E-01					MH 95% CI	1.16-1.41
Mutated	763	866.52	3201	3097.48	Mutated	167	166.39	765	765.61	MH Odds Ratio	0.78
Other	1569	1465.48	5135	5238.52	Other	6	6.61	31	30.39	MH sum chisq	#REF!
Odds Ratio = 0.78, 95% CI = 0.71 - 0.86					Odds Ratio = 1.13, 95% CI = 0.46 - 2.75					MH p-value	#REF!

Sum chisq = 25.19, p-value = 5.21E-07					Sum chisq = 0.07, p-value = 7.91E-01					MH 95% CI	0.71-0.86

SSS1096

Case	Intragenic L1s		Intergenic L1s		Case	Intragenic L1s		Intergenic L1s			
	Observed	Expected	Observed	Expected		Observed	Expected	Observed	Expected		
Conserved	1404	1294.32	4517	4626.68	Mutated	173	170.14	780	782.86	MH Odds Ratio	1.29
Other	928	1037.68	3819	3709.32	Other	0	2.86	16	13.14	MH sum chisq	#REF!
Odds Ratio = 1.28, 95% CI = 1.17 - 1.40					Odds Ratio = -, 95% CI = -					MH p-value	#REF!
Sum chisq = 26.73, p-value = 2.34E-07					Sum chisq = 2.41, p-value = 1.21E-01					MH 95% CI	-
Mutated	928	1037.68	3819	3709.32						MH Odds Ratio	0.78
Other	1404	1294.32	4517	4626.68						MH sum chisq	26.73
Odds Ratio = 0.78, 95% CI = 0.71 - 0.86										MH p-value	2.34E-07
Sum chisq = 26.73, p-value = 2.34E-07										MH 95% CI	0.71-0.86

I1220

Case	Intragenic L1s		Intergenic L1s		Case	Intragenic L1s		Intergenic L1s			
	Observed	Expected	Observed	Expected		Observed	Expected	Observed	Expected		
Conserved	1875	1807.15	6392	6459.85	Conserved	22	27.14	130	124.86	MH Odds Ratio	1.21
Other	457	524.85	1944	1876.15	Other	151	145.86	666	671.14	MH sum chisq	#REF!
Odds Ratio = 1.25, 95% CI = 1.11 - 1.40					Odds Ratio = 0.75, 95% CI = 0.46 - 1.21					MH p-value	#REF!
Sum chisq = 14.49, p-value = 1.41E-04					Sum chisq = 1.40, p-value = 2.36E-01					MH 95% CI	1.09-1.36
Mutated	457	524.85	1944	1876.15	Mutated	151	145.68	665	670.32	MH Odds Ratio	0.82
Other	1875	1807.15	6392	6459.85	Other	22	27.32	131	125.68	MH sum chisq	#REF!
Odds Ratio = 0.80, 95% CI = 0.71 - 0.90					Odds Ratio = 1.35, 95% CI = 0.83 - 2.20					MH p-value	#REF!
Sum chisq = 14.49, p-value = 1.41E-04					Sum chisq = 1.50, p-value = 2.21E-01					MH 95% CI	0.74-0.89

S1259

Case	Intragenic L1s		Intergenic L1s		Case	Intragenic L1s		Intergenic L1s			
	Observed	Expected	Observed	Expected		Observed	Expected	Observed	Expected		
Conserved	2011	1953.83	6927	6984.17	Conserved	89	90.34	417	415.66	MH Odds Ratio	1.23
Other	321	378.17	1409	1351.83	Other	84	82.66	379	380.34	MH sum chisq	#REF!
Odds Ratio = 1.27, 95% CI = 1.12 - 1.45					Odds Ratio = 0.96, 95% CI = 0.69 - 1.34					MH p-value	#REF!
Sum chisq = 13.20, p-value = 2.79E-04					Sum chisq = 0.05, p-value = 8.22E-01					MH 95% CI	1.09-1.39
Mutated	321	378.17	1409	1351.83	Mutated	84	82.48	378	379.52	MH Odds Ratio	0.81
Other	2011	1953.83	6927	6984.17	Other	89	90.52	418	416.48	MH sum chisq	#REF!
Odds Ratio = 0.78, 95% CI = 0.69 - 0.89					Odds Ratio = 1.04, 95% CI = 0.75 - 1.45					MH p-value	#REF!
Sum chisq = 13.20, p-value = 2.79E-04					Sum chisq = 0.06, p-value = 7.99E-01					MH 95% CI	0.72-0.92

3' UTR

Ta SSVs

Case	Intragenic L1s		Intergenic L1s		Case	Intragenic L1s		Intergenic L1s			
	Observed	Expected	Observed	Expected		Observed	Expected	Observed	Expected		
AAGA	129	137.28	499	490.72	AAGA	2	2.14	10	9.86	MH Odds Ratio	0.92
Other	2203	2194.72	7837	7845.28	Other	171	170.86	786	786.14	MH sum chisq	#REF!
Odds Ratio = 0.92, 95% CI = 0.75 - 1.12					Odds Ratio = 0.92, 95% CI = 0.20 - 4.23					MH p-value	#REF!
Sum chisq = 0.68, p-value = 4.10E-01					Sum chisq = 0.07, p-value = 7.86E-01					MH 95% CI	0.75-1.12
ACAG	57	44.81	148	160.19	ACAG	2	1.43	6	6.57	MH Odds Ratio	1.39
Other	2275	2287.19	8188	8175.81	Other	171	171.57	790	789.43	MH sum chisq	#REF!
Odds Ratio = 1.39, 95% CI = 1.02 - 1.89					Odds Ratio = 1.54, 95% CI = 0.31 - 7.70					MH p-value	#REF!
Sum chisq = 4.32, p-value = 3.76E-02					Sum chisq = 0.00, p-value = 9.47E-01					MH 95% CI	1.03-1.88
ACGA	21	15.96	52	57.04						MH Odds Ratio	1.45
Other	2311	2316.04	8284	8278.96						MH sum chisq	2.05
Odds Ratio = 1.45, 95% CI = 0.87 - 2.41										MH p-value	1.52E-01
Sum chisq = 2.05, p-value = 1.52E-01										MH 95% CI	0.87-2.41
ACGG	32	29.29	102	104.71						MH Odds Ratio	1.12
Other	2300	2302.71	8234	8231.29						MH sum chisq	0.32
Odds Ratio = 1.12, 95% CI = 0.75 - 1.68										MH p-value	5.69E-01
Sum chisq = 0.32, p-value = 5.69E-01										MH 95% CI	0.75-1.68
GAGA	1223	1115.94	3882	3989.06	GAGA	5	3.57	15	16.43	MH Odds Ratio	1.27
Other	1109	1216.06	4454	4346.94	Other	168	169.43	781	779.57	MH sum chisq	#REF!
Odds Ratio = 1.27, 95% CI = 1.15 - 1.39					Odds Ratio = 1.55, 95% CI = 0.56 - 4.32					MH p-value	#REF!
Sum chisq = 25.21, p-value = 5.15E-07					Sum chisq = 0.30, p-value = 5.83E-01					MH 95% CI	1.16-1.39
GAGG	10	12.68	48	45.32	GAGG	4	2.14	8	9.86	MH Odds Ratio	0.93
Other	2322	2319.32	8288	8290.68	Other	169	170.86	788	786.14	MH sum chisq	#REF!
Odds Ratio = 0.74, 95% CI = 0.38 - 1.47					Odds Ratio = 2.33, 95% CI = 0.69 - 7.83					MH p-value	#REF!
Sum chisq = 0.73, p-value = 3.93E-01					Sum chisq = 1.06, p-value = 3.03E-01					MH 95% CI	0.50-1.73
GCGA	6	6.78	25	24.22						MH Odds Ratio	0.86
Other	2326	2325.22	8311	8311.78						MH sum chisq	0.11
Odds Ratio = 0.86, 95% CI = 0.35 - 2.09										MH p-value	7.35E-01
Sum chisq = 0.11, p-value = 7.35E-01										MH 95% CI	0.35-2.09
Unclassified	854	969.26	3580	3464.74	Unclassified	160	162.82	752	749.18	MH Odds Ratio	0.77
Other	1478	1362.74	4756	4871.26	Other	13	10.18	44	46.82	MH sum chisq	#REF!
Odds Ratio = 0.77, 95% CI = 0.70 - 0.84					Odds Ratio = 0.72, 95% CI = 0.38 - 1.37					MH p-value	#REF!
Sum chisq = 30.02, p-value = 4.28E-08					Sum chisq = 1.01, p-value = 3.14E-01					MH 95% CI	0.70-0.84

PolyA Signal

Case	Intragenic L1s		Intergenic L1s		Case	Intragenic L1s		Intergenic L1s			
	Observed	Expected	Observed	Expected		Observed	Expected	Observed	Expected		
Conserved	794	771.87	2737	2759.13	Conserved	22	22.32	103	102.68	MH Odds Ratio	1.05
Other	1538	1560.13	5599	5576.87	Other	151	150.68	693	693.32	MH sum chisq	1.13
Odds Ratio = 1.06, 95% CI = 0.96 - 1.16					Odds Ratio = 0.98, 95% CI = 0.60 - 1.60					MH p-value	2.87E-01
Sum chisq = 1.21, p-value = 2.71E-01					Sum chisq = 0.01, p-value = 9.37E-01					MH 95% CI	0.96-1.16
Mutated	1538	1560.13	5599	5576.87	Mutated	151	150.68	693	693.32	MH Odds Ratio	0.95
Other	794	771.87	2737	2759.13	Other	22	22.32	103	102.68	MH sum chisq	1.13
Odds Ratio = 0.95, 95% CI = 0.86 - 1.04					Odds Ratio = 1.02, 95% CI = 0.62 - 1.67					MH p-value	2.87E-01
Sum chisq = 1.21, p-value = 2.71E-01					Sum chisq = 0.01, p-value = 9.37E-01					MH 95% CI	0.86-1.04

Table 2.2 Student's *t-tests* of non-categorical human L1 characteristics.

Student's t-test of non-categorical human L1 characteristics. The total number of L1 sequences containing tested L1 characteristic (N) including the mean, SD, minimum, and maximum values of such characteristic were shown for compared L1 groups.

**L1PA subfamily**						**L1M subfamily**						**All L1 family**					
**Total number of intragenic L1s = 2332**						**Total number of intragenic L1s = 173**						**Total number of intragenic L1s = 2505**					
**Total number of intergenic L1s = 8336**						**Total number of intragenic L1s = 796**						**Total number of intragenic L1s = 9132**					
**Overall**																	
**Length**																	
Independent samples T-test.						Independent samples T-test.						Independent samples T-test.					
Name	N	Mean	SD	Min	Max	Name	N	Mean	SD	Min	Max	Name	N	Mean	SD	Min	Max
Intragenic L1s	2332	7438.36	490.69	6001	9190	Intragenic L1s	173	7124.51	933.43	6007	9328	Intragenic L1s	2505	7416.68	538.82	6001	9328
Intergenic L1s	8336	7408.45	534.52	6001	10570	Intergenic L1s	796	7174.45	976.07	6001	9501	Intergenic L1s	9132	7388.06	590.01	6001	10570
t-statistic = 2.43, p-value = 1.51E-02						t-statistic = -0.61, p-value = 5.39E-01						t-statistic = 2.19, p-value = 2.85E-02					
**find TSDs**																	
Independent samples T-test.						Independent samples T-test.						Independent samples T-test.					
Name	N	Mean	SD	Min	Max	Name	N	Mean	SD	Min	Max	Name	N	Mean	SD	Min	Max
Intragenic L1s	2332	14.4	32.06	0	325	Intragenic L1s	173	14.14	44.17	0	297	Intragenic L1s	2505	14.38	33.03	0	325
Intergenic L1s	8336	12.91	33.36	0	496	Intergenic L1s	796	8.26	23.61	0	288	Intergenic L1s	9132	12.51	32.65	0	496
t-statistic = 1.91, p-value = 5.57E-02						t-statistic = 2.47, p-value = 1.36E-02						t-statistic = 2.53, p-value = 1.13E-02					
**G-C Content**																	
Independent samples T-test.						Independent samples T-test.						Independent samples T-test.					
Name	N	Mean	SD	Min	Max	Name	N	Mean	SD	Min	Max	Name	N	Mean	SD	Min	Max
Intragenic L1s	2332	40.25	1.59	30.65	45.06	Intragenic L1s	173	36.05	2.68	30.05	42.99	Intragenic L1s	2505	39.96	2	30.05	45.06
Intergenic L1s	8336	39.86	1.76	28.53	45.58	Intergenic L1s	796	35.91	2.69	28.92	46.92	Intergenic L1s	9132	39.51	2.17	28.53	46.92
t-statistic = 9.59, p-value = 1.04E-21						t-statistic = 0.63, p-value = 5.29E-01						t-statistic = 9.20, p-value = 4.29E-20					
**ORF1&2 %A**																	
Independent samples T-test.						Independent samples T-test.						Independent samples T-test.					
Name	N	Mean	SD	Min	Max	Name	N	Mean	SD	Min	Max	Name	N	Mean	SD	Min	Max
Intragenic L1s	2331	0.41	0.01	0.38	0.47	Intragenic L1s	167	0.44	0.01	0.41	0.47	Intragenic L1s	2498	0.42	0.01	0.38	0.47
Intergenic L1s	8324	0.42	0.01	0.38	0.48	Intergenic L1s	747	0.44	0.01	0.4	0.48	Intergenic L1s	9071	0.42	0.01	0.38	0.48
t-statistic = -8.03, p-value = 1.11E-15						t-statistic = -1.09, p-value = 2.75E-01						t-statistic = -8.10, p-value = 6.30E-16					
**ORF1&2 %T**																	
Independent samples T-test.						Independent samples T-test.						Independent samples T-test.					
Name	N	Mean	SD	Min	Max	Name	N	Mean	SD	Min	Max	Name	N	Mean	SD	Min	Max
Intragenic L1s	2331	0.2	0.01	0.19	0.27	Intragenic L1s	167	0.23	0.01	0.19	0.28	Intragenic L1s	2498	0.2	0.01	0.19	0.28
Intergenic L1s	8324	0.2	0.01	0.19	0.28	Intergenic L1s	747	0.23	0.01	0.19	0.27	Intergenic L1s	9071	0.21	0.01	0.19	0.28
t-statistic = -11.14, p-value = 1.14E-28						t-statistic = -1.19, p-value = 2.34E-01						t-statistic = -10.12, p-value = 5.65E-24					
**IntactnessScore**																	
Independent samples T-test.						Independent samples T-test.						Independent samples T-test.					
Name	N	Mean	SD	Min	Max	Name	N	Mean	SD	Min	Max	Name	N	Mean	SD	Min	Max
Intragenic L1s	2332	16.88	4.42	2	24	Intragenic L1s	173	7.43	2.42	2	17	Intragenic L1s	2505	16.22	4.93	2	24
Intergenic L1s	8336	16.04	4.71	2	24	Intergenic L1s	796	7.58	2.4	0	19	Intergenic L1s	9132	15.3	5.15	0	24
t-statistic = 7.67, p-value = 1.83E-14						t-statistic = -0.74, p-value = 4.61E-01						t-statistic = 8.01, p-value = 1.29E-15					
**ORF1**																	
**ORF1 gaps**																	
Independent samples T-test.						Independent samples T-test.						Independent samples T-test.					
Name	N	Mean	SD	Min	Max	Name	N	Mean	SD	Min	Max	Name	N	Mean	SD	Min	Max
Intragenic L1s	2332	2.03	4.66	0	99	Intragenic L1s	173	7.71	6.42	0	50	Intragenic L1s	2505	2.42	5.01	0	99
Intergenic L1s	8336	2.27	4.67	0	100	Intergenic L1s	796	7.23	6.32	0	63	Intergenic L1s	9132	2.7	5.03	0	100
t-statistic = -2.19, p-value = 2.86E-02						t-statistic = 0.90, p-value = 3.70E-01						t-statistic = -2.47, p-value = 1.37E-02					
**ORF1 frameshifts**																	
Independent samples T-test.						Independent samples T-test.						Independent samples T-test.					
Name	N	Mean	SD	Min	Max	Name	N	Mean	SD	Min	Max	Name	N	Mean	SD	Min	Max
Intragenic L1s	2332	1.54	1.52	0	8	Intragenic L1s	173	3.15	1.58	0	8	Intragenic L1s	2505	1.66	1.58	0	8
Intergenic L1s	8336	1.72	1.6	0	9	Intergenic L1s	796	3	1.77	0	8	Intergenic L1s	9132	1.83	1.65	0	9
t-statistic = -4.65, p-value = 3.31E-06						t-statistic = 1.00, p-value = 3.16E-01						t-statistic = -4.70, p-value = 2.63E-06					
**ORF1 stops**																	
Independent samples T-test.						Independent samples T-test.						Independent samples T-test.					
Name	N	Mean	SD	Min	Max	Name	N	Mean	SD	Min	Max	Name	N	Mean	SD	Min	Max
Intragenic L1s	2332	2.92	2.76	0	25	Intragenic L1s	173	7.97	4.78	0	24	Intragenic L1s	2505	3.27	3.21	0	25
Intergenic L1s	8336	3.46	3.08	0	24	Intergenic L1s	796	7.67	4.46	0	27	Intergenic L1s	9132	3.83	3.43	0	27
t-statistic = -7.71, p-value = 1.41E-14						t-statistic = 0.77, p-value = 4.39E-01						t-statistic = -7.36, p-value = 1.97E-13					
**ORF1 %A**																	
Independent samples T-test.						Independent samples T-test.						Independent samples T-test.					
Name	N	Mean	SD	Min	Max	Name	N	Mean	SD	Min	Max	Name	N	Mean	SD	Min	Max
Intragenic L1s	2332	0.41	0.01	0.36	0.5	Intragenic L1s	173	0.44	0.02	0.39	0.49	Intragenic L1s	2505	0.41	0.02	0.36	0.5
Intergenic L1s	8336	0.41	0.01	0.33	0.51	Intergenic L1s	796	0.44	0.02	0.35	0.5	Intergenic L1s	9132	0.41	0.02	0.33	0.51
t-statistic = -7.72, p-value = 1.24E-14						t-statistic = 0.48, p-value = 6.30E-01						t-statistic = -7.39, p-value = 1.56E-13					
**ORF1 %T**																	
Independent samples T-test.						Independent samples T-test.						Independent samples T-test.					
Name	N	Mean	SD	Min	Max	Name	N	Mean	SD	Min	Max	Name	N	Mean	SD	Min	Max
Intragenic L1s	2332	0.18	0.01	0.16	0.26	Intragenic L1s	173	0.21	0.02	0.17	0.27	Intragenic L1s	2505	0.19	0.02	0.16	0.27
Intergenic L1s	8336	0.19	0.02	0.15	0.28	Intergenic L1s	796	0.22	0.02	0.17	0.29	Intergenic L1s	9132	0.19	0.02	0.15	0.29
t-statistic = -8.96, p-value = 3.85E-19						t-statistic = -2.00, p-value = 4.55E-02						t-statistic = -9.45, p-value = 3.88E-21					
**ORF1 CAI**																	
Independent samples T-test.						Independent samples T-test.						Independent samples T-test.					
Name	N	Mean	SD	Min	Max	Name	N	Mean	SD	Min	Max	Name	N	Mean	SD	Min	Max
Intragenic L1s	2332	0.66	0.02	0.56	0.71	Intragenic L1s	173	0.63	0.02	0.56	0.71	Intragenic L1s	2505	0.66	0.02	0.56	0.71
Intergenic L1s	8336	0.66	0.02	0.55	0.77	Intergenic L1s	796	0.63	0.02	0.57	0.72	Intergenic L1s	9132	0.66	0.02	0.55	0.77
t-statistic = 4.34, p-value = 1.47E-05						t-statistic = -0.19, p-value = 8.47E-01						t-statistic = 4.88, p-value = 1.08E-06					
**ORF2**																	
**ORF2 gaps**																	
Independent samples T-test.						Independent samples T-test.						Independent samples T-test.					
Name	N	Mean	SD	Min	Max	Name	N	Mean	SD	Min	Max	Name	N	Mean	SD	Min	Max
Intragenic L1s	2332	10.08	22.13	0	443	Intragenic L1s	173	38.38	38.7	0	291	Intragenic L1s	2505	12.03	24.7	0	443
Intergenic L1s	8336	11.96	27.77	0	527	Intergenic L1s	795	34.7	34	0	377	Intergenic L1s	9131	13.94	29.08	0	527
t-statistic = -3.02, p-value = 2.55E-03						t-statistic = 1.26, p-value = 2.10E-01						t-statistic = -3.00, p-value = 2.69E-03					
**ORF2 frameshifts**																	
Independent samples T-test.						Independent samples T-test.						Independent samples T-test.					
Name	N	Mean	SD	Min	Max	Name	N	Mean	SD	Min	Max	Name	N	Mean	SD	Min	Max
Intragenic L1s	2332	7.42	5.75	0	31	Intragenic L1s	173	18.05	5.74	3	30	Intragenic L1s	2505	8.16	6.35	0	31
Intergenic L1s	8336	8.04	6.07	0	33	Intergenic L1s	795	18.17	6.07	0	34	Intergenic L1s	9131	8.93	6.71	0	34
t-statistic = -4.41, p-value = 1.03E-05						t-statistic = -0.24, p-value = 8.08E-01						t-statistic = -5.14, p-value = 2.83E-07					

ORF2 stops

Independent samples T-test.						Independent samples T-test.						Independent samples T-test.					
Name	N	Mean	SD	Min	Max	Name	N	Mean	SD	Min	Max	Name	N	Mean	SD	Min	Max
Intragenic L1s	2332	7.1	7.19	0	59	Intragenic L1s	173	27.39	11.48	1	72	Intragenic L1s	2505	8.5	9.15	0	72
Intergenic L1s	8336	8.68	8.29	0	71	Intergenic L1s	795	27.9	11.55	0	72	Intergenic L1s	9131	10.36	10.18	0	72
t-statistic = -8.41, p-value = 4.64E-17						t-statistic = -0.53, p-value = 5.93E-01						t-statistic = -8.27, p-value = 1.45E-16					

ORF2 %A

Independent samples T-test.						Independent samples T-test.						Independent samples T-test.					
Name	N	Mean	SD	Min	Max	Name	N	Mean	SD	Min	Max	Name	N	Mean	SD	Min	Max
Intragenic L1s	2332	0.41	0.01	0.37	0.48	Intragenic L1s	173	0.44	0.01	0.4	0.47	Intragenic L1s	2505	0.42	0.01	0.37	0.48
Intergenic L1s	8336	0.42	0.01	0.37	0.48	Intergenic L1s	796	0.44	0.01	0.31	0.48	Intergenic L1s	9132	0.42	0.01	0.31	0.48
t-statistic = -7.77, p-value = 8.31E-15						t-statistic = -0.70, p-value = 4.85E-01						t-statistic = -8.01, p-value = 1.30E-15					

ORF2 %T

Independent samples T-test.						Independent samples T-test.						Independent samples T-test.					
Name	N	Mean	SD	Min	Max	Name	N	Mean	SD	Min	Max	Name	N	Mean	SD	Min	Max
Intragenic L1s	2332	0.21	0.01	0.19	0.27	Intragenic L1s	173	0.23	0.02	0.2	0.28	Intragenic L1s	2505	0.21	0.01	0.19	0.28
Intergenic L1s	8336	0.21	0.01	0.19	0.29	Intergenic L1s	796	0.23	0.01	0.2	0.29	Intergenic L1s	9132	0.21	0.01	0.19	0.29
t-statistic = -11.53, p-value = 1.46E-30						t-statistic = -1.82, p-value = 6.90E-02						t-statistic = -10.57, p-value = 5.46E-26					

ORF2 CAI

Independent samples T-test.						Independent samples T-test.						Independent samples T-test.					
Name	N	Mean	SD	Min	Max	Name	N	Mean	SD	Min	Max	Name	N	Mean	SD	Min	Max
Intragenic L1s	2332	0.63	0.01	0.58	0.67	Intragenic L1s	173	0.61	0.01	0.56	0.64	Intragenic L1s	2505	0.63	0.01	0.56	0.67
Intergenic L1s	8336	0.63	0.01	0.57	0.67	Intergenic L1s	796	0.61	0.01	0.57	0.67	Intergenic L1s	9132	0.63	0.01	0.57	0.67
t-statistic = 5.69, p-value = 1.29E-08						t-statistic = 0.88, p-value = 3.76E-01						t-statistic = 6.36, p-value = 2.11E-10					

Definitions of human L1 characteristics

Human L1 sequences were downloaded from L1Base [1]. These elements were annotated with important features for L1 activities. We group them according to where these features can be found, namely, 5' UTR, ORF1, ORF2 and 3' UTR. We put the overall features, e.g., G-C content and cannot be placed according to a specific location on L1 in a group called "Overall". Detailed information on finding of each feature can be found from the cited references. The measurement outputs are in two forms, categorical (e.g., conserved/mutated, L1M/L1PA for chi-square test) and non-categorical (e.g., %A, %T for student's *t*-test).

Overall

- ORF StartStop: check the presence of valid methionine start and stop codons in both ORF1 and ORF2 in the form of (M*, M*). This feature is reported as conserved, ORF1 conserved, ORF2 conserved, or mutated.
- Find TSDs [2,3]: search for target-site-duplications (TSD) flanking the L1 element. These TSDs, which span more than 10 nucleotides (nt), will be considered as valid TSDs. This feature is reported as the number of valid TSDs.
- CpG Islands [4]: count the number of annotated CpG islands. This feature is reported as the number of CpG islands.
- G-C Content [4]: calculate the percentage of G-C of the L1 element in a 50nt window. This feature is reported as G-C percentage.
- Intactness Score: calculate the overall score of features. Every intact feature (conserved) awards one point. This feature is reported as the total points earned.

5' UTR

- Ta1-nd/d [5]: check for the presence of Ta1 subfamily where "nd" is no deletion and "d" is for deletion of Ta1 located in 5' UTR.
- Runx3 Site, Runx3 ASP [6]: check for the presence of the intact RUNX3 and RUNX3 Anti-Sense-Promoter (ASP) binding motifs, respectively.
- SRY Site 1, SRY Site 2 [7]: check for the presence of the intact first and second SRY (sex-determining region Y) binding motifs, respectively.
- YY1 BoxA+BoxA [8,9,10]: check for the presence of an intact YY1 binding motif.
- TF nkx-2.5, TF nkx-2.5B [11]: check for the presence of the first transcription factor Nkx-2.5 and second Nkx-2.5B sites, respectively.

ORF1

- ORF1 conserved: check for the conservation of amino acid sequence of ORF1. If the final score passes the significance cutoff value, it will be reported as "conserved", otherwise mutated.
- REKG235, ARR260, YPAKLS282 [12]: these features are short amino acid sequences at different locations. It is said to be conserved if an amino acid sequence matches otherwise

mutated. For example, check the amino acids position 235 to 238 if they match REKG (REKG235), see note below.

- ORF1 gaps, ORF1 frameshifts, ORF1 stops: count the number of gaps, frameshifts and stop codons (TAA, TAG, TGA) in ORF1, respectively.
- ORF1 %A, ORF1 %T [13], ORF1 CAI [14]: calculate nucleotide percentages of A (%A), T (%T), and the Codon Adaptation Index (CAI) of ORF1, respectively.

ORF2

- ORF2 conserved: check for the conservation of amino acid sequence of ORF2. If the final score passes the significance cutoff value, it will be reported as "conserved", otherwise mutated.
- Ta0/Ta1 SSVs [5,15,16]: determine the shared sequence variant (SSV) subfamily of L1s from Ta0-1 locus including Ta-0/L1PA2, Ta-1, and L1PA5.
- L1M/L1PA Discrimination [17]: check if ORF2 contains either mammalian L1 (L1M) or primate L1 (L1PA).
- N14, E43, Y115, D145, N147, T192, D205, SDH228 [18], R363 [19], FADD700 [18], HMKK1091, SSS1096 [12], I1220, S1259 [19]: check for the conservation of amino acid residues at these particular loci (see note below)
- ORF2 gaps, ORF2 frameshifts, ORF2 stops: count the number of gaps, frameshifts and stop codons (TAA, TAG, TGA) in ORF2.
- ORF2 %A, ORF2 %T [13], ORF2 CAI [14]: calculate nucleotide percentages of A (%A), T (%T), and the Codon Adaptation Index (CAI) of ORF2, respectively.
- ORF1&2 %A, ORF1&2 %T [13]: calculate %A and %T for ORF1&2, respectively.

3' UTR

- Ta SSVs [5,15,16,20]: determine Ta families, including GAGA (L1PA2-L1PA5), GAGG or GCGA (intermediate L1), ACGA or ACGG (preTa L1), and ACAG (Ta Element).
- Poly A Signal [21]: test for conservation of poly A patterns. The consensus 'AATAAA' or 'AATTAAA' are considered as the two valid patterns.

Note

- **Amino acid residues changes:** check for the intactness of amino acid residues on the ORF1 and ORF2. For example, **REKG235** in ORF1 refer to checking of the amino acid residues starting at 235 on the ORF1 start codon whether they match the sequence 'R-E-K-G', respectively. **N14** in ORF2 refer to checking the residue 'N' at the position 14 on the ORF2 start codon.

References

1. Penzkofer T, Dandekar T, Zemojtel T (2004) L1Base: from functional annotation to prediction of active LINE-1 elements. Nucl Acids Res 33: D498–D500.
2. Szak ST, Pickeral OK, Landsman D, Boeke JD (2003) Identifying related L1 retrotransposons by analyzing 3' transduced sequences. Genome Biol 4(5):R30.
3. Szak ST, Pickeral OK, Makalowski W, Boguski MS, Landsman D, Boeke JD. (2002) Molecular archeology of L1 insertions in the human genome. Genome Biol 3(10):research0052.
4. Furano AV (2000) The biological properties and evolutionary dynamics of mammalian LINE-1 retrotransposons. Prog Nucleic Acid Res Mol Biol 64:255-294.
5. Boissinot S, Chevret P, and Furano AV (2000) L1 (LINE-1) retrotransposon evolution and amplification in recent human history. Mol Biol Evol 17(6):915-928.
6. Yang N, Zhang L, Zhang Y and Kazazian HH Jr (2003) An important role for RUNX3 in human L1 transcription and retrotransposition. Nucl Acids Res 31:4929-4940.
7. Tchenio T, Casella JF and Heidmann T (2000) Members of the SRY family regulate the human LINE retrotransposons. Nucl Acids Res 28:411-415.
8. Kurose K, Hata K, Hattori M and Sakaki Y (1995) RNA polymerase III dependence of the human L1 promoter and possible participation of the RNA polymerase II factor YY1 in the RNA polymerase III transcription system. Nucl Acids Res 23:3704-3709.
9. Becker KG, Swergold GD, Ozato K and Thayer RE (1993) Binding of the ubiquitous nuclear transcription factor YY1 to a *cis* regulatory sequence in the human LINE-1 transposable element. Hum Mol Genet 2:1697-1702.
10. Hyde-DeRuyscher RP, Jennings E and Shenk T (1995) DNA binding sites for the transcriptional activator/repressor YY1. Nucl Acids Res 23:4457-4465.
11. Durocher D, Chen CY, Ardati A, Schwartz RJ and Nemer M (1996) The atrial natriuretic factor promoter is a downstream target for Nkx-2.5 in the myocardium. Mol Cell Biol 16: 4648-4655.
12. Moran JV, Holmes SE, Naas TP, DeBerardinis RJ, Boeke JD and Kazazian HH Jr (1996) High frequency retrotransposition in cultured mammalian cells. Cell 87:917-927.
13. Han JS and Boeke JD (2004) A highly active synthetic mammalian retrotransposon. Nature 429:314-318.
14. Sharp PM and Li WH (1987) The codon Adaptation Index--a measure of directional synonymous codon usage bias, and its potential applications. Nucl Acids Res 15:1281-1295.
15. Salem AH, Myers JS, Otieno AC, Watkins WS, Jorde LB and Batzer MA (2003) LINE-1 preTa elements in the human genome. J Mol Biol 326:1127-1146.
16. Boissinot S, Entezam A, Young L, Munson PJ and Furano AV (2004) The insertional history of an active family of L1 retrotransposons in humans. Genome Res 14:1221-1231.
17. Smit AF, Toth G, Riggs AD and Jurka J (1995) Ancestral, mammalian-wide subfamilies of LINE-1 repetitive sequences. J Mol Biol 246:401-417.

18. Feng Q, Moran JV, Kazazian HH Jr and Boeke JD (1996) Human L1 retrotransposon encodes a conserved endonuclease required for retrotransposition. Cell 87:905-916.
19. Lutz SM, Vincent BJ, Kazazian HH Jr, Batzer MA and Moran JV (2003) Allelic heterogeneity in LINE-1 retrotransposition activity. Am J Hum Genet 73:1431-1437.
20. Ovchinnikov I, Rubin A and Swergold GD (2002) Tracing the LINEs of human evolution. PNAS 99:10522-10527.
21. Boeke JD (1997) LINEs and Alus--the polyA connection. Nat Genet 16:6-7.
