# Supplementary material for: Roles of Intragenic and Intergenic L1s in Mouse and Human
Source: PLoS One. 2014 Nov 19;9(11):e113434. doi: 10.1371/journal.pone.0113434 (PMC4237456; doi:10.1371/journal.pone.0113434)
Supplement: Table S3 — Molecular functions of 14 orthologous down-regulated genes during early embryogenesis in human and mouse. (PDF) [file pone.0113434.s003.pdf]

Table 3. Molecular functions of 14 orthologous down-regulated genes during early embryogenesis in human and mouse

Mouse gene	Mouse GeneID	Mouse gene function	Human gene	Human GeneID	Human gene function	Function from GeneCards
Kcnq1	16535	calmodulin binding, delayed rectifier potassium channel activity, ion channel activity, outward rectifier potassium channel activity, potassium channel activity, voltage-gated ion channel activity, voltage-gated potassium channel activity	KCNQ1	3784	calmodulin binding, delayed rectifier potassium channel activity, outward rectifier potassium channel activity, voltage-gated potassium channel activity	Probably important in cardiac repolarization. Associates with KCNE1 (MinK) to form the I(Ks) cardiac potassium current. Elicits a rapidly activating, potassium-selective outward current. Muscarinic agonist oxotremorine-M strongly suppresses KCNQ1/KCNE1 current in CHO cells in which cloned KCNQ1/KCNE1 channels were coexpressed with M1 muscarinic receptors. May associate also with KCNE3 (MiRP2) to form the potassium channel that is important for cyclic AMP-stimulated intestinal secretion of chloride ions, which is reduced in cystic fibrosis and pathologically stimulated in cholera and other forms of secretory diarrhea.
Rad51l1	19363	ATP binding, DNA binding, DNA-dependent ATPase activity, nucleoside-triphosphatase activity, nucleotide binding	RAD51B	5890	ATP binding, DNA binding, DNA-dependent ATPase activity, protein binding	Involved in the homologous recombination repair (HRR) pathway of double-stranded DNA breaks arising during DNA replication or induced by DNA-damaging agents. May promote the assembly of presynaptic RAD51 nucleoprotein filaments. The RAD51B-RAD51C dimer exhibits single-stranded DNA-dependent ATPase activity. The BCDX2 complex binds single-stranded DNA, single-stranded gaps in duplex DNA and specifically to nicks in duplex DNA.
Rabgap1l	29809	GTPase activator activity, Rab GTPase activator activity, Rab GTPase binding	RABGAP1L	9910	Rab GTPase activator activity, Rab GTPase binding	
Fut8	53618	SH3 domain binding, alpha-(1->6)-fucosyltransferase activity, glycoprotein 6-alpha-L-fucosyltransferase activity, transferase activity, transferase activity, transferring glycosyl groups	FUT8	2530	SH3 domain binding, glycoprotein 6-alpha-L-fucosyltransferase activity	Catalyzes the addition of fucose in alpha 1-6 linkage to the first GlcNAc residue, next to the peptide chains in N-glycans.
Pde3a	54611	3',5'-cyclic-AMP phosphodiesterase activity, 3',5'-cyclic-AMP phosphodiesterase activity, 3',5'-cyclic-nucleotide phosphodiesterase activity, cAMP binding, cGMP-inhibited cyclic-nucleotide phosphodiesterase activity, catalytic activity, hydrolase activity, metal ion binding, phosphoric diester hydrolase activity	PDE3A	5139	3',5'-cyclic-AMP phosphodiesterase activity, cAMP binding, cGMP-inhibited cyclic-nucleotide phosphodiesterase activity, metal ion binding	Cyclic nucleotide phosphodiesterase with a dual-specificity for the second messengers cAMP and cGMP, which are key regulators of many important physiological processes (By similarity).
Lmbr1	56873	-	LMBR1	64327	-	Putative membrane receptor.
Vav3	57257	Rac guanyl-nucleotide exchange factor activity, Rho guanyl-nucleotide exchange factor activity, epidermal growth factor receptor binding, guanyl-nucleotide exchange factor activity, metal ion binding, phospholipid binding, protein binding	VAV3	10451	GTPase activator activity, Rac guanyl-nucleotide exchange factor activity, SH3/SH2 adaptor activity, epidermal growth factor receptor binding, metal ion binding, phospholipid binding, protein binding	Exchange factor for GTP-binding proteins RhoA, RhoG and, to a lesser extent, Rac1. Binds physically to the nucleotide-free states of those GTPases. Plays an important role in angiogenesis. Its recruitment by phosphorylated EPHA2 is critical for EFNA1-induced RAC1 GTPase activation and vascular endothelial cell migration and assembly (By similarity). May be important for integrin-mediated signaling, at least in some cell types. In osteoclasts, along with SYK tyrosine kinase, required for signaling through integrin alpha-v/beta-1 (ITAGV-ITGB1), a crucial event for osteoclast proper cytoskeleton organization and function. This signaling pathway involves RAC1, but not RHO, activation. Necessary for proper wound healing. In the course of wound healing, required for the phagocytotic cup formation preceding macrophage phagocytosis of apoptotic neutrophils. Responsible for integrin beta-2 (ITGB2)-mediated macrophage adhesion and, to a lesser extent, contributes to beta-3 (ITGB3)-mediated adhesion. Does not affect integrin beta-1 (ITGB1)-mediated adhesion (By similarity).
Rsrc1	66880	protein binding	RSRC1	51319	protein binding	Plays a role in pre-mRNA splicing. Involved in both constitutive and alternative pre-mRNA splicing. May have a role in the recognition of the 3' splice site during the second step of splicing.
Ccdc132	73288	molecular_function	CCDC132	55610	-	-
Tusc3	80286	magnesium ion transmembrane transporter activity	TUSC3	7991	dolichyl-diphosphooligosaccharide-protein glycotransferase activity, magnesium ion transmembrane transporter activity	Magnesium transporter. May be involved in N-glycosylation through its association with N-oligosaccharyl transferase.
Hivep1	110521	DNA binding, DNA binding, HMG box domain binding, metal ion binding, nucleic acid binding, protein binding, zinc ion binding	HIVEP1	3096	DNA binding, protein binding, zinc ion binding	This protein specifically binds to the DNA sequence 5'-GGGACTTTCC-3' which is found in the enhancer elements of numerous viral promoters such as those of SV40, CMV, or HIV-1. In addition, related sequences are found in the enhancer elements of a number of cellular promoters, including those of the class I MHC, interleukin-2 receptor, and interferon-beta genes. It may act in T-cell activation. Involved in activating HIV-1 gene expression. Isoform 2 and isoform 3 also bind to the IPCS (IRF1 and p53 common sequence) DNA sequence in the promoter region of interferon regulatory factor 1 and p53 genes and are involved in transcription regulation of these genes. Isoform 2 does not activate HIV-1 gene expression. Isoform 2 and isoform 3 may be involved in apoptosis.
Rims2	116838	Rab GTPase binding, ion channel binding, metal ion binding, protein binding, protein domain specific binding, protein heterodimerization activity	RIMS2	9699	Rab GTPase binding, metal ion binding, protein binding	Rab effector involved in exocytosis. May act as scaffold protein.
Tox	252838	DNA binding	TOX	9760	DNA binding	May play a role in regulating T-cell development (By similarity).
Cntn4	269784	-	CNTN4	152330	-	Contactins mediate cell surface interactions during nervous system development. Has some neurite outgrowth-promoting activity. May be involved in synaptogenesis.
